# Afrotropical flea beetle genera: a key to their identification, updated catalogue and biogeographical analysis (Coleoptera, Chrysomelidae, Galerucinae, Alticini)

**DOI:** 10.3897/zookeys.253.3414

**Published:** 2012-12-20

**Authors:** Maurizio Biondi, Paola D’Alessandro

**Affiliations:** 1 Department of Health, Life and Environmental Sciences, University of L’Aquila, 67100 Coppito-L’Aquila, Italy

**Keywords:** Taxonomy, Afrotropical region, Chrysomelidae, Galerucinae, Alticini, flea beetle genera, identification key, catalogue, synonymies, new combinations, status promotus

## Abstract

A revision of the Alticini genera from the Afrotropical region is reported. The paper includes the following for the flea beetle fauna occurring in Sub-Saharan Africa and Madagascar: a key to their identification; habitus photos of all the genera; microscope and scanning electron micrographs of many diagnostic morphological characters; and an updated annotated catalogue with biogeographical notes that include new distributional data. The following new synonymies are proposed: *Aphthona* Chevrolat, 1836 = *Ethiopia* Scherer, 1972 **syn. n.**; *Sanckia* Duvivier, 1891 = *Eugonotes* Jacoby, 1897 **syn. n.**; *Eurylegna* Weise, 1910a = *Eurylegniella* Scherer, 1972 **syn. n.**; *Kimongona* Bechyné, 1959a = *Mesocrepis* Scherer, 1963 **syn. n.**; *Diphaulacosoma* Jacoby, 1892a = *Neoderina* Bechyné, 1952 **syn. n.**; *Sesquiphaera* Bechyné, 1958a = *Paropsiderma* Bechyné, 1958a **syn. n.**; *Podagrica* Chevrolat, 1836 = *Podagricina* Csiki in Heikertinger and Csiki 1940 **syn. n.**; *Amphimela* Chapuis, 1875 = *Sphaerophysa* Baly, 1876a **syn. n.** The following new combinations are proposed: *Blepharida insignis* Brancsik, 1897 = *Xanthophysca insignis* (Brancsik, 1897) **comb. n.**; *Blepharida multiguttata* Duvivier, 1891 = *Xanthophysca multiguttata* (Duvivier, 1891) **comb. n.**; *Hemipyxis balyana* (Csiki in Heikertinger and Csiki 1940) = *Pseudadorium balyanum* (Csiki in Heikertinger and Csiki, 1940) **comb. n.**; *Hemipyxis brevicornis* (Jacoby, 1892a) = *Pseudadorium brevicornis* (Jacoby, 1892a) **comb. n.**; *Hemipyxis cyanea* (Weise, 1910b) = *Pseudadorium cyaneum* (Weise, 1910b) **comb. n.**; *Hemipyxis gynandromorpha* Bechyné, 1958c = *Pseudadorium gynandromorphum* (Bechyné, 1958c) **comb. n.**; *Hemipyxis latiuscula* Bechyné, 1958c = *Pseudadorium latiusculum* (Bechyné, 1958c) **comb. n.**; *Hemipyxis soror* (Weise, 1910b) = *Pseudadorium soror* (Weise, 1910b) **comb. n.** The genera *Buphonella* Jacoby, 1903aand *Halticopsis* Fairmaire, 1883a are transferred to the tribe Galerucini; the genus *Biodontocnema* Biondi, 2000 **stat. prom.** is considered to be valid and reinstated at generic level. Finally, a zoogeographical analysis of the flea beetle fauna in the Afrotropical region is provided.

## Introduction

The Chrysomelidae is one of the largest phytophagous insect families and includes approximately 37,000 to 40,000 species (Jolivet and Verma 2002). The monophyletic tribe Alticini is closely related to the tribe Galerucini, both contained within in the subfamily Galerucinae (Bouchard et al. 2011). The relationship between these two tribes, often considered as different subfamilies, is an area of active research regarding Chrysomelidae phylogeny (Duckett et al. 2004; Gómez-Zurita et al. 2007; Ge et al. 2011, 2012). In our paper, Alticini and Galerucini are considered to be separate suprageneric taxa because of the metafemoral spring in Alticini, as well as specific structures of the spermatheca, median lobe of aedeagus and hind wing venation of these two groups (cf. Furth and Suzuki, 1994, 1998). In our opinion, some of the recently established groupings, based on DNA sequences, still need further in-depth analysis because they are phylogenetically and biogeographically incomplete (cf. Ge et al. 2011, 2012). The Alticini is a tribe composed of minute to medium sized beetles, whose enlarged hind femora and renowned jumping ability have earned them the common name of ‘flea beetles’. They are highly specialised phytophagous insects. Both the adult and larval stages feed on stems, leaves or roots, and rarely on flowers, in almost all the higher plant families (Konstantinov and Vandenberg 1996). The tribe Alticini includes 4,000 to 8,000 species, grouped in approximately 500 genera. This taxon has a world-wide distribution, but occurs mainly in the tropical regions of South America, Africa and Asia (Konstantinov and Vandenberg 1996; Santiago-Blay 2004; Biondi and D’Alessandro 2010a).

**Figure 1.**
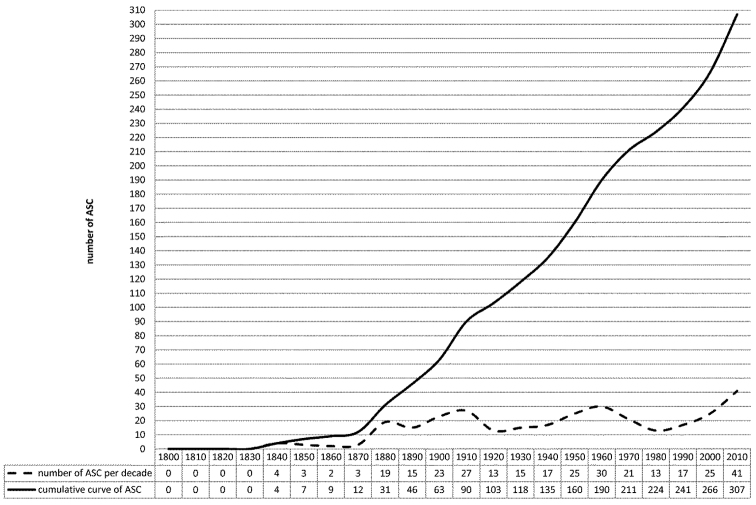
Chronology of publications on the Afrotropical flea beetle fauna. ASC: Afrotropical Scientific Contributions (update from Biondi and D’Alessandro 2010a).

We recently published an annotated catalogue of the Afrotropical flea beetle genera, based largely on data from the literature (Biondi and D’Alessandro 2010a). Subsequent to a deeper and more extensive examination of type material, and the study of new Afrotropical flea beetle material preserved in the institutions listed below, it was possible to compile an updated catalogue that contains new synonymies, new combinations, new genera and new distribution records. Even so, many details concerning the composition of the Afrotropical flea beetle fauna remain incomplete (Biondi and D’Alessandro 2010a). The discrepancy in the number of morphogenera, and morphospecies in particular, preserved in public and private collections of African entomological material, and those that have been officially described, highlights this shortcoming. Current scientific literature includes over 300 research papers dedicated as a whole, or in part, to Sub-Saharan and Madagascan Alticini. These publications include contributions on taxonomy, faunistics and ecology (Biondi and D’Alessandro 2010a). The chronological trend in the number of publications over time is summarized in Fig. 1, the first appearing as early as 1830. However, the first significant contribution on the Afrotropical (including Madagascar) flea beetle fauna was made by the English coleopterist, Joseph Sugar Baly (1816−1890). Subsequently, in the twenty years following Baly’s death (1890−1910), there were three respected entomologists working on this fauna: Léon Fairmaire (1820−1906), a French specialist on Coleoptera and Hemiptera; Julius Weise (1844−1925), a German coleopterist that, during his life, published a large number of scientific papers, not only on Chrysomelidae but also on Coccinellidae, Curculionoidea and others; and Martin Jacoby (1842−1907), a German musician and coleopterist, who published 150 articles on leaf beetles after moving to London.

A decrease in the number of publications on the Afrotropical flea beetle fauna followed, until a revival in 1930−1940, initiated by the English coleopterist Gilbert Ernest Bryant (1878−1965) and the French chrysomelid specialist Victor Laboissière (1875−1942). Jan Bechyné (1920−1973) and Gerhard Scherer (1929-2012), specialists on the Alticinae, then published many monographs (see References) on the flea beetle fauna of Sub-Saharan Africa and, to a lesser extent, Madagascar. They described many new genera and species between 1950 and 1970. More recently, contributions on the Afrotropical flea beetle fauna were published by Gerhard Scherer, Maurizio Biondi, Paola D’Alessandro, Manfred Döberl, Serge Doguet, and Elizabeth Grobbelaar (see References).

## Materials and methods

The catalogue is arranged alphabetically by generic names. Names in bold refer to flea beetle genera primarily occurring in the Afrotropical region; those in square brackets refer to: synonymies; genera incorrectly reported in the Afrotropical region; in some cases genera transferred to Galerucini; or genus-group names that are unavailable. The rules of the International Commission on Zoological Nomenclature (1999) are adhered to the Fourth Edition of the International Code of Zoological Nomenclature.

In addition to the author and date of publication, each genus-group name is accompanied by: a) synonymies, exclusively those for the Afrotropical region; b) bibliographic references, including the original description, other important taxonomic contributions, and distribution data; c) type species, including the method of species assignment; d) geographic distribution in the Afrotropical region (cf. Graf and Cummings 2007) and other zoogeographical regions (cf. Sclater 1858); e) ecological remarks, mainly host-plants and/or habitat preferences in the Afrotropical region; f) notes, including the number of Afrotropical species and significant taxonomic information.

Specimens were examined and dissected using WILD MZ12.5 and LEICA M205C binocular microscopes. Photomicrographs were taken using a Leica DFC500 camera and the Auto-Montage Pro 2006 software (license number: 15224*syn2459*153a2112_maurizio_266836). Scanning electron micrographs were taken using a PHILIPS SEM XL30 CP and HITACHI TM-1000. Morphometric measures were taken using the image analysis software Image-Pro Insight 8.0 (license number: 03080000-5385).

The type material examined during this study is preserved in the following institutions: BAQ: collection of M. Biondi, University of L’Aquila, Italy; BMNH: The Natural History Museum, London, United Kingdom; ISNB: Institut Royal des Sciences Naturelles de Belgique, Brussels, Belgium; MNHN: Muséum National d’Histoire Naturelle, Paris, France; MCSN: Museo Civico di Storia Naturale ‘Giacomo Doria’, Genova, Italy; MRAC: Musée Royal de l’Afrique Centrale, Tervuren, Belgium; MUAF: Mendel University of Agriculture and Forestry, Brno, Czech Republic; MZHF: Finnish Museum of Natural History, University of Helsinki, Finland; MZLU: Lund University, Sweden; NHMB: Naturhistorisches Museum, Basel, Switzerland; NHRS: Naturhistoriska Riksmuseet, Stockholm, Sweden; NMPC: Entomologické Oddělení Národního Muzea, Praha-Kunratice, Czech Republic; SANC: South African National Collection, ARC-Plant Protection Research Institute, Pretoria, South Africa; SMNS: Staatliches Museum für Naturkunde, Stuttgart, Germany; TMSA: Ditsong: National Museum of Natural History (formerly Transvaal Museum), Pretoria, South Africa; ZMHB: Museum für Naturkunde der Humboldt-Universität, Berlin, Germany; ZSM: Zoologische Staatssammlung, Munich, Germany.

Abbreviations.Morphology - LAN: length of antennae; LB: total length of body; LE: length of elytra; LHT: length of hind tibia; LHTS: length of hind tibial spur; LP: length of pronotum; WE: width of elytra; WP: width of pronotum. Regions - AFR: Afrotropical; AUR: Australian; CAF: Central Afrotropical; EAF: Eastern Afrotropical; ORR: Oriental; MAD: Madagascar; MAS: Mascarene Islands; NAR: Nearctic; NTR: Neotropical; PAR: Palaearctic; SAF: Southern Afrotropical; SEY: Seychelles Islands; SSA: Sub-Saharan Africa; WAF: Western Afrotropical. (?) record to be confirmed; (!) new record; (i) introduced.

## Key to the identification of afrotropical flea beetle genera

A new key for the identification of the Afrotropical flea beetle genera is proposed. In comparison with the key previously proposed by Scherer (1961), our key includes all the known flea beetle genera occurring in Sub-Saharan Africa, Madagascar, Seychelles and the Mascarene Islands. The first key below identifies eight different generic groups, labelled with an uppercase letter (group A, group B etc.). Our generic groups are, on the whole, similar to the seven numbered groups (group 1, group 2 etc.) proposed by Scherer (1961). For widespread genera, we have primarily concentrated on the morphological and chromatic variability displayed by those species of the genus in question, known to occur in the Afrotropical region.

### Key to groups

**Table:** 

1	Antennae with 10 antennomeres	Group A
–	Antennae with 11 antennomeres.	2
2	Apical tarsomere of metatarsus distinctly swollen (Figs 200, 237)	Group B
–	Apical tarsomere of metatarsus not swollen (Figs 144, 149, 166, 169)	3
3	Dorsal margin of middle and hind tibiae with distinct ciliate dentate emargination, acute or subrounded apically (Figs 144, 148, 154, 163, 258)	Group C
–	Dorsal margin of middle and hind tibiae without distinct ciliate dentate emargination	4
4	Pronotum with a distinct but poorly defined median depression near each lateral margin with surface more strongly punctate (Fig. 135)	Group D
–	Pronotum uniformly or sparsely punctate, but never with more strongly punctate median depression near lateral margin	5
5	Pronotum with ante-basal transverse sulcus (Figs 120, 121, 126, 206, 209, 212, 225, 246)	Group E
–	Pronotum without ante-basal transverse sulcus	6
6	Pronotal base with two short sublateral longitudinal striae (Figs 85–86) [sometimes also with two distinct longitudinal grooves on anterior pronotal margin (Figs 217–218)]	Group F
–	Pronotal base without short sublateral longitudinal striae	7
7	Pronotum with distinct sublateral mesal callosity bounded by more or less deeply impressed diagonal sulcus laterally (Figs 133, 153)	Group G
–	Pronotum without sublateral callosity	Group H

### Group A

**Table:** 

1	Elytral punctation arranged in regular rows (Fig. 91). Metatarsus preapically inserted on tibia (Fig. 248). Procoxal cavities closed posteriorly	*Psylliodes* Berthold, 1827 (Fig. 91)
–	Elytral punctation confused (Fig. 32). Metatarsus apically inserted on tibia. Procoxal cavities open posteriorly	*Decaria* Weise, 1895 (Fig. 32)

### Group B

**Table:** 

1	Elytral punctation arranged in regular rows (Fig. 109). Procoxal cavities closed posteriorly	*Zomba* Bryant, 1922 (Fig. 109)
–	Elytral punctation confused. Procoxal cavities open posteriorly	2
2	Elytral epipleura vertically orientated in distal 2/3s, not visible in lateral view (Fig. 238)	3
–	Elytral epipleura horizontally or obliquely downward orientated in distal 2/3s, visible in lateral view (Figs 184, 229, 233)	4
3	Pronotum with anterior angles distinctly produced towards anterior and distinctly thickened; posterior angles rounded (Fig. 236). Hind tibiae often curved towards inside (Fig. 237). First metatarsomere wide and subtriangular (Fig. 237). Apical tarsomere of metatarsus moderately swollen (Fig. 237)	*Physomandroya* Bechyné, 1959 (Fig. 83)
–	Pronotum with anterior angles not thickened, dentiform apically, indistinctly produced forwards anterior; posterior angles dentiform apically (Fig. 199). Hind tibiae straight (Fig. 200). First metatarsomere narrow, subcylindrical (Fig. 200). Apical tarsomere of metatarsus distinctly swollen (Fig. 200)	*Hyphasis* Harold, 1877 (Fig. 54)
4	Vertex of head distinctly and densely punctate (Fig. 239). Frontal tubercles wide, subtriangular, well defined, and closely associated lengthwise (Fig. 239)	*Physonychis* Clark, 1860 (Fig. 84)
–	Vertex of head smooth or indistinctly and sparsely punctate (Figs 234–235). Frontal tubercles small, generally poorly defined (Figs 234–235)	5
5	Frons distinctly sharp-edged distally in lateral view or clearly produced anteriorly, forming a smooth wide surface, often with evident laminate extensions (Figs 234–235)	*Physoma* Clark, 1863 (Fig. 82)
–	Frons arcuate distally in lateral view, never with eversions or laminate extensions	6
6	Pronotum distinctly rounded laterally, generally at least 2x wider than long (WP/LP > 2.00); pronotal base depressed, sometimes with distinct transverse ante-basal sulcus, more distinctly impressed near posterior angles of pronotum (Fig. 78)	*Philopona* Weise, 1903 (Fig. 78)
–	Pronotum almost straight or very slightly rounded laterally, less than 2x wider than long (WP/LP ≤ 2.00); pronotal base generally not depressed, never with distinct transverse ante-basal sulcus (Figs 183, 232)	7
7	Elytral epipleura obliquely downward orientated in apical 2/3s, easily visible in lateral view (Fig. 184). Elytra subparallel or widening slightly laterally towards posterior, distinctly bordered and finely channeled laterally (Fig. 44). Pronotum generally more transverse (WP/LP > 1.90), indistinctly bordered laterally, with not uniformly distributed punctation (Fig. 183). Dorsal integument always with strongly impressed punctation (Figs 44, 183, 184). Elytra pale brown with longitudinal black stripes (Fig. 44). Body smaller (generally LB ≤ 7.50 mm)	*Eutornus* Clark, 1860 (Fig. 44)
–	Elytral epipleura horinzontally orientated in apical 2/3s, little visible in lateral view (Fig. 233). Elytra widening distinctly laterally towards posterior, narrowly bordered laterally (Fig. 81). Pronotum generally less transverse (WP/LP ≤ 1.90), finely bordered laterally, with uniformly distributed punctation (Fig. 232). Dorsal integument generally from finely to moderately punctate (Figs 81, 233). Elytra unicolor, never with longitudinal black stripes (Fig. 81). Body larger (generally LB > 7.50 mm)	*Physodactyla* Chapuis, 1875 (Fig. 81)

## Group C

**Table:** 

1	First metatarsomere distinctly shorter than second (Fig. 267). Elytral punctation arranged in 11 rows (+1 long sutural row), partially irregular only in sutural area (Fig. 102). Prothorax subcylindrical. Eyes roundish, distinctly protuberant (Fig. 266)	*Terpnochlorus* Fairmaire, 1904 (Fig. 102)
–	First metatarsomere as long as second or longer (Figs 117, 121, 124, 163). Elytral punctation confused or arranged in 9 single or double rows (+1 short sutural row). Prothorax distinctly depressed dorsally. Eyes generally subelliptical, not distinctly protuberant (Figs 146, 150, 158)	2
2	First metatarsomere narrow, subcylindrical or subrectangular (Figs 144, 154, 258). Claws simple or subappendiculate (Figs 144, 154). Body smaller (generally LB < 4.00 mm)	3
–	First metatarsomere wide, subtriangular (Figs 148, 149). Claws generally appendiculate (Fig. 162). Body larger (generally LB ≥ 4.00 mm)	7
3	Hind tibiae with dorsal margin distinctly bidentate (Figs. 144, 258)	4
–	Hind tibiae with dorsal margin unidentate (Fig. 154) [in *Chaetocnema schlaeflii* (Stierlin, 1866) and *Chaetocnema major* (Jacquelin du Val, 1852), the dorsal tibial margin may appear bidentate, but *Seychellaltica* is easily distinguishable from these two species mainly by having the frontal sulci very short, not visible around eyes (Fig. 252), and the first pro- and mesotarsomeres distinctly asymmetrical (Fig. 254); while *Biodontocnema* is easily distinguishable by having a wider socket on the hind tibia with inner margin dentiform (Figs 142–144); weakly developed metasternum (Fig. 145); antennomeres 6–10 distinctly shorter, as long as wide (Figs 22); and first metatarsomere that is laterally compressed (Fig. 142); in addition, *Biodontocnema* has shorter antennae that do not reach half of the elytral length and distinctly smaller size (LB > 3.00 mm; LB < 2.50 mm) (Fig. 22)]	5
4	Frontal sulci very short, not visible around eyes (Fig. 257). Antennae longer, reaching half of elytral length (Fig. 97). Antennomeres 6–10 distinctly longer than wide (Fig. 97). Body elongate, with pronotum distinctly transverse (generally WP/LP > 1.57); humeral calli distinct (Fig. 97). Ratio of metasternal width/metasternal length ≤ 2.50 (Fig. 260). First metatarsomere at least twice as long as second and third together, not laterally compressed (Fig. 258). First pro- and mesotarsomeres distinctly asymmetrical (Fig. 259). Hind tibial socket narrow without dentiform inner margin (Fig. 258)	*Seychellaltica* Biondi, 2002 (Fig. 97)
–	Frontal sulci elongate, distinctly visible around eyes (Fig. 141). Antennae shorter, only reaching base of elytra (Fig. 22). Antennomeres 6–10 about as long as wide. Body oval, with pronotum slightly transverse (generally WP/LP ≤ 1.57); humeral calli absent (Fig. 22). Ratio of metasternal width/metasternal length > 2.50 (Fig. 145). First metatarsomere shorter than second and third together, and laterally compressed (Figs 142–144). First pro- and mesotarsomeres symmetrical. Hind tibial socket wide with distinctly dentiform inner margin (Figs 142–143)	*Biodontocnema* Biondi, 2000 stat. prom. (Fig. 22)
5	Interocular space with at least a distinct transverse carina (Fig. 150). Distal margin of frons distinctly incised medially (Fig. 150). Interantennal space at least twice as wide as length of first antennomere (Fig. 150)	*Carcharodis* Weise, 1910 (Fig. 25)
–	Interocular space without transverse carinae. Distal margin of frons medially not incised. Interantennal space less than twice as wide as of first antennomere length (Fig. 159)	6
6	Prosternum distinctly convex anteriorly (Fig. 160), most of mouthparts fitting into this convexity when head is in resting position, except labrum and mandibles, which act as a ‘cover’ (Fig. 159); maxillae and labium sunken (Fig. 159). Pronotal punctation variable, displaying punctures of different sizes (Fig. 158)	*Collartaltica* Bechyné, 1959 (Fig. 31)
–	Prosternum moderately convex anteriorly, mouthparts do not fit into this convexity when head is in resting position; maxillae and labium exposed and not sunken. Pronotal punctation uniform	*Chaetocnema* Stephens, 1831 (Fig. 28)
7	Procoxal cavities closed posteriorly. Antennomere 4 about as long as antennomere 3, or shorter (Figs 147, 273, 274). Elytral punctation generally arranged in regular rows, more rarely partially in irregular double rows or confused (Figs 23, 107). Antennae shorter, not reaching half elytral length (Figs 23, 107)	8
–	Procoxal cavities open posteriorly. Antennomere 4 at least double length of antennomere 3 (Figs 161, 240, 241). Elytral punctation always confused, densely and uniformly impressed (Figs 33, 87). Antennae longer, reaching half elytral length (Figs 33, 87)	9
8	Pronotum with distinct apical, median or basal impressions, always with two sublateral series of large and deeply impressed punctures from anterior margin to middle of pronotum (subgenus *Blepharidina* Bechyné) (Fig. 146); pronotal punctation not uniformly distributed (Fig. 146). Hind tibiae broadly channeled dorsally (Fig. 148). Frontal sulci deeply impressed (subgenus *Blepharidina* Bechyné) (Fig. 147). Eyes generally very elongate longitudinally (Fig. 147). Elytral punctation from moderately to distinctly impressed, generally arranged in regular rows (Figs 23, 148) (in Afrotropical region only *Blepharida geminata* Bryant, 1944 shows elytral punctation arranged in regular partially double rows). Posterior margin of hind femora indistinctly or moderately emarginated	*Blepharida* Chevrolat, 1836 (Fig. 23)
–	Pronotum without distinct impressions; pronotal punctation uniformly distributed (Figs 107, 273). Hind tibiae indistinctly channeled dorsally (Fig. 275). Frontal sulci moderately impressed (Fig. 274). Eyes less elongate longitudinally, sometimes roundish (Fig. 274). Elytral punctation moderately impressed, arranged in double partially regular rows (Figs 107, 275) or mostly confused. Posterior margin of hind femora often distinctly emarginated (Fig. 275)	*Xanthophysca* Fairmaire, 1901 (Fig. 107)
9	Antennae filiform with antennomeres 4–10 filiform or slightly enlarged (Fig. 161)	*Diamphidia* Gerstaecker, 1855 (Fig. 33)
–	Antennae with antennomeres 4–10 pectinate or flabellate in male and serrate in female (Figs 240–241)	*Polyclada* Chevrolat, 1836 (Fig. 87)

## Group D

**Table:** 

1	Pronotum with two very short but distinct longitudinal sublateral impressions basally; transverse sulcus absent ante-basally (Fig. 103). Elytral punctation confused, finely but distinctly and densely impressed, mainly in basal half (Fig. 103). Body larger (LB > 3.90 mm), subsphaerical (Fig. 103)	*Toxaria* Weise, 1903 (Fig. 103)
–	Pronotum with transverse sulcus ante-basally, sometimes very slightly impressed. Elytral punctation generally very slightly or indistinctly impressed (Fig. 101), sometimes subseriate (Fig. 18). Body smaller (LB ≤ 3.90 mm), oval or elliptical (Fig. 18, 101)	2
2	Pronotum comparatively smaller (LE/LP > 2.80), slightly rounded laterally; transverse sulcus always distinctly impressed ante-basally, bounded by two distinct longitudinal striae laterally (Fig. 101)	*Stuckenbergiana* Scherer, 1963 (Fig. 101)
–	Pronotum comparatively larger (LE/LP ≤ 2.80), more distinctly rounded laterally; transverse sulcus very slightly impressed ante-basally, not bounded laterally and sometimes only visible medially (Fig. 18)	*Bechuana* Scherer, 1970 (Fig. 18)

## Group E

**Table:** 

1	Dorsal integument distinctly pubescent (Fig. 40, 41)	2
–	Dorsal integument apparently glabrous. Elytra sometimes very sparsely pubescent in *Lampedona* and *Lypnea*	3
2	Pronotum as wide as elytra basally, subparallel or convergent towards anterior laterally; sulcus more or less deeply impressed ante-basally, bounded by two short longitudinal striae laterally (Fig. 40). Frontal tubercles very small, elongate and narrow (Fig. 180). Antennomere 4 distinctly shorter than antennomeres 2–3 together. Body less elongate (LB/WE < 2.00) (Fig. 41). Elytra not modified apically in male	*Epitrix* Foudras, 1860 (Fig. 40)
–	Pronotum narrower than elytra basally, divergent towards anterior laterally; transverse sulcus finely impressed ante-basally, not bounded by longitudinal striae laterally (Fig. 41). Frontal tubercles larger, subrectangular or subtriangular, often elongate towards upper ocular margin (Fig. 181). Antennomere 4 as long as antennomeres 2–3 together. Body more elongate (LB/WE ≥ 2.00) (Fig. 41). Elytra generally with modified structures apically in male (Fig. 41)	*Eriotica* Harold, 1877 (Fig. 41)
3	Hind tibia with two apical spurs (Fig. 216)	*Myrcina* Chapuis, 1875 (Fig. 69)
–	Hind tibia with only one apical spur	4
4	Pronotum with transverse sulcus bounded laterally by two short longitudinal striae ante-basally or touching basal margin (Figs 126, 170, 209, 225, 246)	5
–	Pronotum with transverse sulcus not bounded by longitudinal striae laterally and ante-basally; sulcus interrupted laterally, or touching lateral margin or posterior angles (Figs 120, 121, 212)	20
5	Antennomere 3 distinctly longer than antennomere 1 (Figs 12, 73). Frons distinctly elongate (Figs 127, 222, 223). Genae about 1.5x length of vertical ocular diameter (Figs 127, 223). Antennae comparatively elongate (LB/LAN < 1.20) (Figs 12, 73)	6
–	Antennomere 3 shorter than antennomere 1 (Fig. 210). Frons short. Genae distinctly shorter than vertical ocular diameter. Antennae comparatively short (LB/LAN ≥ 1.20)	7
6	Elytral punctation arranged in 9 regular rows, partially confused in sutural area; interstriae distinctly convex on disc, and subcarinate laterally (Figs 73, 222). Anterior angles of pronotum not bevelled, distinctly dentiform apically (Fig. 221). Antennae extraordinarily elongate in male (LB/LAN < 0.90), with antennomeres 3–6 distinctly enlarged; antennae in female not enlarged and little shorter than the body (Fig. 73). Body elongate and slightly convex (Figs 73, 222)	*Ntaolaltica* Biondi & D’Alessandro, in press (Fig. 73)
–	Elytral punctation entirely confused (Fig. 12). Anterior angles of pronotum bevelled, moderately dentiform apically (Fig. 126). Antennae filiform in both sexes (LB/LAN ≥ 0.90), without enlarged antennomeres or only moderately enlarged only in male (Fig. 12). Body thickset and distinctly convex (Fig. 12)	*Antanemora* Bechyné, 1964 (Fig. 12)
7	Pronotum with ante-basal transverse sulcus not bounded by longitudinal striae laterally, and touching basal margin (Figs 170, 246)	8
–	Pronotum with ante-basal transverse sulcus bounded by two short longitudinal striae laterally (Fig. 225)	13
8	Elytral punctation arranged in regular, or sometimes partially irregular, single or double rows	9
–	Elytral punctation entirely confused	10
9	Antennae in female robust, generally with antennomere 2 as long as antennomere 3; antennomeres 9–11 in male distinctly enlarged, about as wide as long (Fig. 90). Elytral punctation indistinctly impressed, sometimes hardly visible amongst interstrial punctuation (Fig. 247). Pronotum distinctly rounded and widely bordered laterally (Fig. 246)	*Pseudophygasia* Biondi & D’Alessandro, in press (Fig. 90)
–	Antennae filiform with antennomere 2 distinctly shorter than antennomere 3; antennomeres 9–11 elongate (Fig. 210). Elytral punctation distinctly impressed (Fig. 64). Pronotum slightly rounded and finely bordered laterally (Fig. 209)	*Lypnea* Baly, 1876a (Fig. 64)
10	Pronotum distinctly arcuate laterally, distinctly narrower basally; posterior angles distinctly laterally produced at apex (Fig. 170). Frontal tubercles rounded, distinctly raised (Figs 170–171). Antennae more robust, often with distinctly enlarged distal antennomeres (Fig. 171)	*Diphaulacosoma* Jacoby, 1892a (Fig. 36)
–	Pronotum moderately rounded, slightly narrower basally; posterior angles very indistinctly produced laterally, finely dentiform at apex. Frontal tubercles generally subtriangular, flat or slightly raised. Antennae filiform, never with distinctly enlarged antennomeres	11
11	Frontal tubercles extended distally between antennae, forming two parallel, acute apically longitudinal carinae which surround clypeus distally (Fig. 207). Body smaller (generally LB < 2.60 mm). Pronotum with distinctly impressed transverse sulcus ante-basally (Fig. 61)	*Lepialtica* Scherer, 1962 (Fig. 61)
–	Frontal tubercles not extended distally between antennae; frontal carina simple. Body larger (generally LB ≥ 2.60 mm). Pronotum with indistinctly impressed transverse sulcus ante-basally	12
12	Elytra widely bordered laterally (Fig. 77). Frontal carina narrow and acute apically (Fig. 228). Interantennal space narrow, about as wide as 1/3 of length of first antennomere (Fig. 228)	*Perichilona* Weise, 1919 (Fig. 77)
–	Elytra narrowly bordered laterally (Fig. 42). Frontal carina wider and rounded apically (Fig. 182). Interantennal space wider, about as wide as length of first antennomere (Fig. 182)	*Eurylegna* Weise, 1910 (Fig. 42)
	[= *Eurylegniella* Scherer, 1972 syn. n. (Fig. 43)]
13	Elytral punctation arranged in simple or double rows, sometimes partially irregular	14
–	Elytral punctation entirely confused	17
14	Elytral punctation arranged in double partially irregular rows (Fig. 118). Elytra generally with a distinct longitudinal carina laterally (Fig. 118)	*Afrocrepis* Bechyné, 1954 (Fig. 4)
–	Elytral punctation arranged in single regular rows. Elytra never with longitudinal carinae laterally	15
15	Anterior tibiae distinctly enlarged distally, with a deep longitudinal depression in distal half (Fig. 188). Interocular space distinctly punctate (Fig. 187)	*Guinerestia* Scherer, 1959 (Fig. 47)
–	Anterior tibiae normally shaped. Interocular space impunctate or very sparsely punctate (Figs 119, 205)	16
16	Procoxal cavities open posteriorly. Pronotal sulcus curved towards base medially, bounded by two short longitudinal striae laterally (Figs 58, 59). Elytra with distinct basal calli (Figs 58, 59). Frontal carina narrow, acute apically (Fig. 205). Frontal tubercles next to each other (Fig. 205)	*Kimongona* Bechyné, 1959 (Fig. 58)
	[= *Mesocrepis* Scherer, 1963 syn. n. (Fig. 59)]
–	Procoxal cavities closed posteriorly. Pronotal sulcus straight, bounded by two longer longitudinal striae laterally (Fig. 5). Elytra without basal calli (Fig. 5). Frontal carina wide, subrounded apically (Fig. 119). Frontal tubercles distant from each other (Fig. 119)	*Afrorestia* Bechyné, 1959 (Fig. 5)
17	Antennae comparatively short (LB/LAN ≥ 2.20), with antennomeres 6–11 as long as wide (Fig. 10)	*Anaxerta* Fairmaire, 1902 (Fig. 10)
–	Antennae comparatively long (LB/LAN < 2.20), with antennomeres 6–11 distinctly longer than wide	18
18	Procoxal cavities closed posteriorly. Pronotum moderately rounded laterally; posterior angles not dentiform apically (Fig. 70). Antennae often with alternate groups of black and yellowish antennomeres (Fig. 70)	*Neodera* Duvivier, 1891 (Fig. 70)
–	Procoxal cavities open posteriorly. Pronotum distinctly rounded laterally; posterior angles distinctly dentiform apically. Antennae never with alternate groups of black and yellowish antennomeres	19
19	Antennomere 2 about as long as antennomere 3 or slightly shorter (Fig. 225). Frontal tubercles indistinctly defined posteriorly, and slightly raised (Fig. 226) . Pronotum narrowly bordered laterally Fig. 225)	*Orthocrepis* Weise, 1888 (Fig. 75)
–	Antennomere 2 distinctly shorter than antennomere 3 (Fig. 230). Frontal tubercles distinctly defined posteriorly, and distinctly raised (Fig. 230). Pronotum widely bordered laterally (Fig. 79)	*Phygasia* Chevrolat, 1836 (Fig. 79)
20	Elytral punctation entirely confused [see also *Guilielmia* at couplet 24]	21
–	Elytral punctation arranged in simple or double rows, more or less irregular only in *Guilielmia*	23
21	Procoxal cavities closed posteriorly (157). Anterior and middle femora enlarged, particularly in male (Fig. 157)	*Chirodica* Germar, 1834 (see also Group H couplet 28) (Fig. 30)
–	Procoxal cavities open posteriorly. Anterior and middle femora not enlarged	22
22	Pronotum with distinctly impressed transverse sulcus ante-basally, often touching lateral margins of pronotum (Fig. 121); anterior angles generally rounded apically. Interantennal space narrower than length of first antennomere (Fig. 121). Frontal carina narrow and acute (Fig. 121). Pronotal punctation generally indistinctly impressed (Fig. 121). Dorsal integument usually blue or green, always with distinct metallic luster (Fig. 7)	*Altica* Geoffroy, 1762 (Fig. 7)
–	Pronotum with slightly impressed transverse sulcus ante-basally, never touching lateral margins of pronotum (Fig. 1); anterior angles obliquely bevelled apically . Interantennal space distinctly wider than length of first antennomere (Fig. 120). Frontal carina wide and flat. Pronotal punctation distinctly impressed (Fig. 120). Dorsal integument brownish with partially blackened elytra, without metallic luster (Fig. 6)	*Alocypha* Weise, 1911 (Fig. 6)
23	Humeral calli absent. Ante-basal pronotal sulcus barely visible (Figs 26, 46)	24
–	Humeral calli distinct. Ante-basal pronotal sulcus always distinctly impressed, medially at least (Figs 172, 206, 212)	25
24	Elytral punctation arranged in 9 regular rows. Dorsal integument from yellowish to brownish	*Celisaltica* Biondi, 2001a (Fig. 26)
–	Elytral punctation more or less irregular. Dorsal integument dark	*Guilielmia* Weise, 1924 (Fig. 46)
25	Antennomere 2 as long as antennomere 1 and at least twice as long as antennomere 3 (Fig. 172). Procoxal cavities closed posteriorly	*Djallonia* Bechyné, 1955 (Fig. 37)
–	Antennomere 2 distinctly shorter than antennomere 1 and about as long as antennomere 3. Procoxal cavities open posteriorly	26
26	Antennomere 1 at least 2.5 times longer than antennomere 2. Pronotal base not, or very slightly, sinuous (Fig. 206). Body larger (LB ≥ 4.50 mm). Elytra comparatively elongate (LE/WE > 1.60) (Fig. 60)	*Lampedona* Weise, 1907 (Fig. 60)
–	Antennomere 1 about 1.5 times longer than antennomere 2. Pronotal base distinctly sinuous (Figs 204, 212). Body smaller (LB < 4.50 mm). Elytra comparatively short (LE/WE ≤ 1.60) (Figs 57, 66)	27
27	Frontal tubercles distinctly defined posteriorly. Pronotum with distinctly impressed transverse sulcus ante-basally, touching lateral margins of pronotum; posterior angles distinctly dentiform apically (Fig. 212). Dorsal integument with distinctly impressed punctation (Fig. 66). First metatarsomere longer than second and third together. Elytra with prominent basal calli (Fig. 66)	*Manobia* Jacoby, 1885 (Fig. 66)
–	Frontal tubercles not distinctly defined posteriorly. Pronotum with a moderately or indistinctly impressed transverse sulcus ante-basally, not touching the lateral margins of pronotum; posterior angles subrounded apically, never distinctly dentiform (Fig. 204). Dorsal integument with slightly or moderately impressed punctation (Fig. 57). First metatarsomere shorter than second and third together. Elytra with basal calli barely visible (Fig. 57).	*Kenialtica* Bechyné, 1960 (Fig. 57)

## Group F

**Table:** 

1	Anterior margin of pronotum without distinct longitudinal impressions, with very short incisions at most. Pronotum slightly narrower than elytra basally. Body generally less convex and more elongate (Figs 85–86)	*Podagrica* Chevrolat, 1836 (Fig. 85)
	[= *Podagricina* Csiki in Heikertinger and Csiki 1940 syn. n. (Fig. 86)]
–	Anterior margin of pronotum with two longitudinal groove-like impressions, often deeply impressed (Figs 217–218) and sometimes reaching middle of pronotum. Pronotum as wide as elytra basally (Figs 71, 217, 218). Body thickset and distinctly convex (Fig. 71).	*Nisotra* Baly, 1864 (Fig. 71)

## Group G

**Table:** 

1	Body more elongate, less convex, with comparatively elongate elytra (LE/WE < 1.25) (Fig. 16). Elytral margin in dorsal view well visible in its all length. Pronotum with straight sublateral sulci, basally obliquely slanted and distally never touching the lateral margin of pronotum (Fig. 133).	*Argopistoides* Jacoby, 1892b (Fig. 16)
–	Body subsphaerical, distinctly convex, with comparatively short elytra (LE/WE ≥ 1.25) (Fig. 27). Elytral margin in dorsal view generally not visible or visible only basally. Pronotum with sinuous sublateral sulci, basally starting sub-parallel to lateral margin of pronotum and distally sometimes touching it (Fig. 153)	*Chabria* Jacoby, 1887 (Fig. 27)

## Group H

**Table:** 

1	Apical spur of hind tibiae distinctly serrate (Fig. 252, 253, 277)	2
–	Apical spur of hind tibiae differently shaped but never serrate	3
2	Elytral punctation distinctly impressed, arranged in regular rows (Fig. 251). First metatarsomere longer than 2/3s length of hind tibia and dorsally or preapically inserted on hind tibia (Fig. 252). Apical spur of hind tibiae distinctly elongate (LHT/LHTS ≤ 8.00) (Figs 252–253)	*Serraphula* Jacoby, 1897 (Fig. 94)
–	Elytral punctation slightly impressed, poorly visible and only partially arranged in regular rows (Fig. 276). First metatarsomere at most as long as half of hind tibial length and apically inserted on hind tibia (Fig. 276). Apical spur of hind tibiae very short (LHT/LHTS > 8.00) (Figs 276–277)	*Yemenaltica* Scherer, 1985 (Fig. 108)
3	Metatarsus dorsally inserted at about half of tibial length (Fig. 213)	*Metroserrapha* Bechyné, 1958a (Fig. 67)
–	Metatarsus apically inserted on tibia	4
4	Apical spur of hind tibiae distally bifid or trifid (Figs 179, 270)	5
–	Apical spur of hind tibiae simple [in *Trachytetra* hind apical spur thickset, subtruncate, sometimes apparently bifid (Fig. 269); but this genus is easily distinguishable from *Dunbrodya* and *Dibolia* by having distinctly defined frontal tubercles; and frons that is elongate distally in lateral view]	8
5	Apical spur of hind tibiae obtusely pointed apically (Fig. 270). Antennae about as long as body (Fig. 105). Habitus similar to *Longitarsus *	*Tritonaphthona* Bechyné, 1960 (Fig. 105)
–	Apical spur of hind tibiae bifid apically (Fig. 179). Antennae generally short, at most reaching half of elytra	6
6	First metatarsomere about 1/3 of length of hind tibia (Fig. 178). Pronotum less transverse, lateral margins converging slightly towards anterior (WP/LP ≤ 1.70) (Fig. 176). Frons with distinct longitudinal carina medially (Fig. 177). Body elongate and slightly convex (Fig. 39). Habitus similar to *Aphthona *	*Dunbrodya* Jacoby, 1906 (Fig. 39)
–	First metatarsomere about 1/5 of length of hind tibia (Fig. 166). Pronotum distinctly transverse, laterally margins converging distinctly towards anterior (WP/LP > 1.70) (Fig. 164). Frons without or with very short longitudinal carina medially (Figs 165, 227). Body thickset and distinctly convex (Figs 34, 76)	7
7	Eyes small, widely separated dorsally, interocular distance at least 3 times wider than length of second antennomere (Fig. 165)	*Dibolia* Latreille, 1829 (Fig. 34)
–	Eyes very large, closely associated dorsally; interocular space as wide as length of second antennomere (Fig. 227)	*Paradibolia* Baly, 1875 (Fig. 76)
8	Elytra distinctly reduced, obliquely truncate apically (Fig. 262). Pronotum subtrapezoidal, with maximum width at anterior angles; lateral margins straight (Fig. 261)	*Sjostedtinia* Weise, 1910 (Fig. 98)
–	Elytra not reduced, generally rounded apically. Pronotum subrectangular or subtrapezoidal with maximum width generally at middle or basally; lateral margins more or less rounded	9
9	Elytral surface distinctly and uniformly pubescent	10
–	Elytral surface apparently glabrous, rarely very sparsely setose towards apex	14
10	First metatarsomere about half length of tibia (Fig. 250). Elytral punctation arranged in regular rows (Figs 92–93)	*Sanckia* Duvivier, 1891 (Fig. 92)
	[= *Eugonotes* Jacoby, 1897 syn. n. (Fig. 93)]
–	First metatarsomere distinctly shorter than half length of tibia. Elytral punctation confused	11
11	Pronotum pubescent	*Hespera* Weise, 1889 (Fig. 51)
–	Pronotum glabrous	12
12	Elytral punctation confused, densely and finely impressed (Figs 53, 198). Elytral surface with short dense pubescence (Fig. 198). Frontal tubercles not defined posteriorly. Antennae short, not reaching middle of elytra (Fig. 53). First metatarsomere shorter than length of second and third together (Fig. 198). Elytral margins widely bordered laterally (Fig. 198)	*Homichloda* Weise, 1902 (Fig. 53)
–	Elytral punctation arranged in regular or partially irregular rows, distinctly impressed (Figs 49, 52). Elytral surface with longer sparse pubescence (Fig. 191). Frontal tubercles well defined posteriorly (Figs 190, 196, 197). Antennae longer, reaching beyond middle of elytra. First metatarsomere longer than second and third together (Fig. 191). Elytral margins narrowly bordered laterally (Fig. 191)	13
13	Pronotal punctation distinctly impressed on surface with distinct transverse and longitudinal carinae, sulci and/or protuberances (Figs 196–197). Frontal tubercles medially separated by deep longitudinal sulcus (Figs 196–197)	*Hildebrandtina* Weise, 1910 (Fig. 52)
–	Pronotal punctation indistinctly impressed on surface without distinct impressions or protuberances Fig. 189). Frontal tubercles medially separated by fine longitudinal sulcus (Fig. 190)	*Halticotropis* Fairmaire, 1886 (Fig. 49)
14	Body slightly elongate, often subsphaerical (LB/WE < 1.70). Pronotum more transverse (generally WP/LP > 1.80)	15
–	Body distinctly elongate, never subsphaerical (LB/WE ≥ 1.70). Pronotum less transverse (generally WP/LP ≤ 1.70 )	24
15	Pronotum with anterior setigerous pore near middle of lateral margin (Fig. 201). Antennomere 3 as long as, or longer than, antennomeres 4–5 together (Fig. 202)	*Jacobyana* Maulik, 1926 (Fig. 55)
–	Pronotum with anterior setigerous pores near anterior angles. Antennomere 3 distinctly shorter than antennomeres 4–5 together	16
16	Body smaller (LB < 1.60 mm). Elytral punctation with scutellar stria long, reaching apical declivity of elytra (Fig. 138). Apical spur of hind tibia very small and slender	*Bezdekaltica* Döberl, 2012 (Fig. 20)
–	Body larger (LB ≥ 1.60 mm). Elytral punctation with scutellar stria short, not reaching middle of elytra. Apical spur of hind tibia robust	17
17	Pronotum with lateral margins diverging from base towards middle, then converging slightly towards anterior; maximum pronotal width in anterior third; anterior angles distinctly dentiform apically (Fig. 219). Pronotal punctation very distinctly impressed especially laterally (Fig. 219). Elytra with submarginal stria of distinctly and deeply impressed punctures laterally, delimiting wide and distinctly raised lateral band (Fig. 220)	*Notomela* Jacoby, 1899 (Fig. 72)
–	Pronotum with lateral margins converging distinctly towards anterior; maximum pronotal width at base; anterior angles not distinctly dentiform apically. Pronotal punctation from finely to moderately impressed. Elytra lacking submarginal stria with distinctly impressed punctures and distinctly raised lateral band	18
18	Frontal tubercles and frontal carina absent (Fig. 122). Interantennal space at least as wide as transverse ocular diameter (Fig. 122). Elytral interstriae always densely punctuated	*Amphimela* Chapuis, 1875 (Fig. 8)
	[= *Sphaerophysa* Baly, 1876b syn. n. (Fig. 9)]
–	Frontal tubercles distinctly defined. Frontal carina narrow, often raised. Interantennal space narrower than transverse ocular diameter. Elytral interstriae generally not densely punctuated	19
19	Pronotal base regularly rounded, not sinuate	20
–	Pronotal base bisinuate	22
20	Elytral punctation regularly striate. Scutellum not visible (Fig. 265). Body smaller (LB < 2.30 mm), distinctly convex (Fig. 100)	*Stegnaspea* Baly, 1877 (Fig. 100)
–	Elytral punctation confused and finely impressed. Scutellum distinctly visible. Body larger (LB ≥ 2.30 mm), moderately convex	21
21	Frontal carina not prolonged towards clypeus (Fig. 244). Frons distinctly raised distally in lateral view. Elytra with distinct basal calli (Fig. 89). Pronotum with anterior angles distinctly thickened, projecting distinctly towards anterior (Fig. 243). Body more convex (Fig. 89). Hind tibiae narrowly and less deeply channeled dorsally (Fig. 245)	*Pseudadorium* Fairmaire, 1885 (Fig. 89)
–	Frontal carina prolonged towards clypeus (Fig. 193). Frons not raised distally in lateral view. Elytra without distinct basal calli (Fig. 50). Pronotal anterior angles slightly or moderately thickened, not projecting towards anterior, but sometimes dentiform laterally (Fig. 192). Body less convex (Fig. 50). Hind tibiae broadly and more deeply channeled dorsally (Fig. 194)	*Hemipyxis* Chevrolat, 1836 (Fig. 50)
22	Hind tibiae with distinct preapical tooth on inside (Fig. 132). Hind femora at least as wide as length of hind tibia (Fig. 131). Eyes elongate, closely associated dorsally, separated by less than transverse ocular diameter (Fig. 130). First abdominal ventrite medially with two distinct longitudinal ridges forward convergent (Fig. 131)	*Argopistes* Motschulsky, 1860 (Fig. 15)
–	Hind tibiae lacking distinct preapical tooth on inside. Hind femora narrower than length of hind tibia. Eyes rounded, widely separated dorsally, separated by transverse ocular diameter at least. First abdominal ventrite without distinct longitudinal ridges	23
23	Pronotum wider (WP/LP ≥ 2.30), distinctly bisinuate basally; anterior angles projecting distinctly towards anterior; apically widely rounded and completely bordered by thickened margin (Figs 254–255). Elytral epipleura obliquely upward orientated, generally not visible in lateral view (Fig. 256). Elytral punctation confused, finely impressed (Figs 95–96). Body subsphaerical (Figs 95–96)	*Sesquiphaera* Bechyné, 1958 (Fig. 95)
	[= *Paropsiderma* Bechyné, 1958 syn. n. (Fig. 96)]
–	Pronotum narrower (WP/LP < 2.30), moderately bisinuate basally; anterior angles projecting slightly towards anterior; apically not completely bordered by thickened margin, limited to lateral edge of pronotum (Fig. 263). Elytral epipleura horizontally or slightly obliquely downward orientated, well visible in lateral view (Fig. 264). Elytral punctation often more or less seriate, punctures more distinctly impressed. Body generally short and oval, rarely subsphaerical (Fig. 99)	*Sphaeroderma* Stephens, 1831 (Fig. 99)
24	Apical spur of hind tibiae robust, often very short, generally absent on anterior and middle tibiae. Antennomere 2 about as long as antennomere 4 (only in some *Chirodica* antennomere 4 distinctly longer than antennomere 2 but anterior and middle femora enlarged and procoxal cavities closed posteriorly). Elytra apparently not pubescent. Body slightly elongate	25
–	Apical spur very slender and present on all tibiae; hind apical spur always elongate but sometimes very short on anterior and middle tibiae. Antennomere 2 much shorter than antennomere 4. Elytra or apical part of elytra very sparsely pubescent (Fig. 174). Body distinctly elongate. Habitus similar to Galerucini	37
25	First metatarsomere as long or longer than half tibial length (Figs 137, 140, 208)	26
–	First metatarsomere shorter than half tibial length	28
26	Elytral punctation confused	*Longitarsus* Berthold, 1827 (Fig. 62)
–	Elytral punctation regularly seriate	27
27	First metatarsomere about same length as tibia (Fig. 137). Interantennal space about as wide as transverse ocular diameter; frontal carina apically rounded, moderately raised (Fig. 136). Apical spur of hind tibiae long (LHT/LHTS ≤ 10.00) (Fig. 137). Dorsal integument from yellowish to pale brown (Fig. 19)	*Bechynella* Biondi & D’Alessandro, 2010b (Fig. 19)
–	First metatarsomere about half length of tibia (Fig. 140). Interantennal space distinctly narrower than transverse ocular diameter; frontal carina apically acute and raised (Fig. 139). Apical spur of hind tibiae shorter (LHT/LHTS > 10.00). Dorsal integument generally darker with more or less distinct metallic reflections (Figs 21, 140)	*Bikasha* Maulik, 1931 (also see couplet 34) (Fig. 21)
28	Procoxal cavities closed posteriorly (Fig. 157). Anterior and middle femora distinctly enlarged, particularly in male (Fig. 157). Pronotum sometimes with a very small transverse sulcus ante-basally. Second maxillary palpomere about as wide as first (Fig. 156)	*Chirodica* Germar, 1834 (also see Group E couplet 21) (Fig. 30)
–	Procoxal cavities open posteriorly. Anterior and middle femora not enlarged. Pronotum never with a transverse sulcus ante-basally. Second maxillary palpomere generally wider than first	29
29	Antennomeres 7–11 or 8–11 more enlarged than those remaining (Figs 2, 3, 111)	30
–	Antennomeres 7–11 similar in width to those remaining	31
30	Humeral calli absent (Fig. 3). Pronotum distinctly convex, very narrowly bordered laterally; anterior angles rounded or slightly bevelled; posterior angles rounded (Fig. 114). Metasternum shorter than length of mid-coxal cavity (Fig. 117). Middle and hind tibiae distinctly enlarged from base to apex, particularly in male; middle tibiae with distinct triangular hollow ventrally in male (Fig. 115)	*Afroaltica* Biondi & D’Alessandro, 2007 (Fig. 3)
–	Humeral calli distinct (Figs 2, 110). Pronotum moderately convex, distinctly bordered laterally; anterior angles distinctly dentiform and widely bevelled; posterior angles acute, often dentiform (Fig. 110). Metasternum longer than length of mid-coxal cavity (Fig. 112). Middle and hind tibiae moderately enlarged from base to apex; middle tibiae without distinct hollow ventrally in male	*Abrarius* Fairmaire, 1902 (Fig. 2)
31	Frontal carina narrow and distinctly raised with few large superficial punctures, and a narrow longitudinal groove distally between antennae (Fig. 242). Elytral and often also pronotal punctation, exceptionally dense and strongly impressed (Fig. 88)	*Pratima* Maulik, 1931 (Fig. 88)
–	Frontal carina wide, flat or convex but never grooved longitudinally. Elytral punctation from indistinctly to moderately impressed	32
32	Anterior angles of pronotum not obliquely bevelled (Fig. 214). Humeral calli absent (Figs 68, 215). Elytral apex subtruncate (Fig. 215)	*Montiaphthona* Scherer, 1961 (Fig. 68)
–	Anterior angles of pronotum obliquely bevelled. Humeral calli generally visible. Elytral apex generally rounded	33
33	Frontal tubercles absent or indistinctly defined (Fig. 231). Anterior angles of pronotum not widely and obliquely bevelled (Fig. 231). Elytral punctation confused. Body flatter and more elongate (Fig. 80)	*Phyllotreta* Chevrolat, 1836 (Fig. 80)
–	Frontal tubercles distinctly defined. Anterior angles of pronotum widely and obliquely bevelled. Elytral punctation sometimes partially regularly striate. Body more convex and less elongate	34
34	Apical spur of hind tibiae thickset, subtruncate apically, often apparently bifid (Fig. 269). Frons elongate distally in lateral view (Fig. 268). Elytral punctation confused	*Trachytetra* Sharp, 1886 (Fig. 104)
–	Apical spur of hind tibiae slender, acute apically, never apparently bifid. Frons short and regularly arcuate distally in lateral view. Elytral punctation confused, seriate or subseriate	35
35	Elytra with poorly defined but distinct basal calli (Fig. 140). Humeral calli bounded posteriorly by distinct, often deeply impressed, depression (Fig. 140). Frontal tubercles always well defined (Fig. 140). Elytral punctation seriate or subseriate, always distinctly impressed, and never confused (Fig. 140)	*Bikasha* Maulik, 1931 (see also couplet 27) (Fig. 21)
–	Elytra lacking basal calli. Humeral calli bounded posteriorly by more or less flat area. Frontal tubercles often not well defined. Elytral punctation generally confused, rarely seriate or subseriate; in this case punctation is slightly impressed and frontal tubercles are generally not distinct	36
36	Pronotum subtrapezoidal, straight or very slightly rounded and widely bordered laterally; anterior angles generally thickened, distinctly dentiform apically; posterior angles with distinct, laterally produced, tubercle at apex (Fig. 123). Pronotal base finely but distinctly sinuate (Fig. 123). Frontal tubercles elongate, V-shaped (Fig. 124). Elytra more widely bordered laterally, always with distinctly impressed punctation (Fig. 125). Dorsal integuments blue with distinct metallic reflections (Figs 11, 125). Spermatheca with coiled ductus	*Angulaphthona* Bechyné, 1960 (Fig. 11)
–	Pronotum subrectangular, more distinctly rounded and finely bordered laterally; anterior angles not thickened, not dentiform apically; posterior angles subrounded apically or slightly dentiform, without distinct tubercles (Fig. 128). Pronotal base generally not sinuate (Fig. 128). Frontal tubercles subtriangular or roundish, rarely elongate. Elytra narrowly bordered laterally, generally with slightly or moderately impressed punctation (Fig. 129). Dorsal integument varies in colour. Spermatheca with uncoiled ductus, very rarely coiled	*Aphthona* Chevrolat, 1836 (Fig. 13)
	[= *Ethiopia* Scherer, 1972 syn. n. (Fig. 14)]
37	Pronotum with distinct oblique or transverse sublateral impressions medially (Figs 17, 29, 74)	38
–	Pronotum without impressions	40
38	Antennomeres 3 and 4 about same length, each about three times longer than antennomere 2; antennomeres 6–11 shorter and subequal in length (Fig. 134)	*Bangalaltica* Bechyné, 1960 (Fig. 17)
–	Antennomere 3 very much shorter than antennomere 4 (Figs 155, 224)	39
39	Antennomere 1 as long as antennomeres 2–4 together (Fig. 155). Antennae in male with antennomeres 2–3 very strongly reduced and 6–7 distinctly enlarged (Fig. 155). Pronotum distinctly more transverse (WP/LP > 1.65) (Fig. 29)	*Chaillucola* Bechyné, 1968 (Fig. 29)
–	Antennomere 1 much shorter than antennomeres 2–4 together (Fig. 224). Antennae similar in both sexes. Pronotum distinctly less transverse (WP/LP ≤ 1.65)	*Nzerekorena* Bechyné, 1955 (Fig. 74)
40	First metatarsomere distinctly compressed laterally (Figs 169, 272)	41
–	First metatarsomere not compressed laterally	42
41	Antennomeres 4–11 subglobose; antennomere 3 about twice as long as antennomere 4 (Fig. 271). Hind femora without processes or projections	*Upembaltica* Bechyné, 1960 (Fig. 106)
–	Antennomeres 4–11 not subglobose; antennomere 3 as long as antennomere 4 (Fig. 167). Hind femora of male with a distinct subtriangular, dentiform process, situated medially on ventral side (Fig. 168)	*Dimonikaea* Bechyné, 1968 (Fig. 35)
42	Antennomere 4 about as long as antennomere 3, but considerably shorter than antennomere 5; antennomere 5 about as long as antennomeres 2–4 together (Fig. 203). Female unknown	*Kanonga* Bechyné, 1960 (Fig. 56)
–	Antennomere 4 longer than antennomere 3 or as long as antennomere 5	43
43	Antennomeres 8–10 very small, subglobose, each distinctly shorter than antennomeres 4–7 (Fig. 211)	*Malvernia* Jacoby, 1899 (Fig. 65)
–	Antennomeres differently shaped. In *Gabonia*, males often with antennae with distinctly modified segments, but never as in *Malvernia*	44
44	Metasternum shorter than length of mid-coxal cavity (Fig. 175). Legs very elongate, particularly hind femora and tibiae (Fig. 38). Antennae normally longer than body, particularly in male (Fig. 38). Elytra comparatively short (LE/LP ≤ 2.60); humeral calli absent (Fig. 38) . Wings vestigial	*Drakensbergianella* Biondi & D’Alessandro, 2003 (Fig. 38)
–	Metasternum at least 1.5x longer than length of mid-coxal cavity (Fig. 186). Legs not elongate (Figs 45, 63). Antennae very rarely longer than body (Figs 45, 63). Elytra comparatively elongate [LE/LP > 2.60 (normally > 3.00)]; humeral calli present. Wings well developed	*Luperomorpha* Weise, 1887 (Fig. 63)
	[*Gabonia* Jacoby, 1893 (see Notes on page 48) (Fig. 45)]

## Catalogue of afrotropical flea beetle genera

### Abrarius

Fairmaire, 1902

http://species-id.net/wiki/Abrarius

Figs 2 110–112 278

=Entymosina Weise, 1910 (synonymized by Bechyné 1958c)

#### References.

Fairmaire 1902: 261; Weise 1910b: 438; Bechyné 1947a: 44 (as *Entymosina*); 1958c: 9; Biondi and D’Alessandro 2010a: 402.

#### Type species.

*Abrarius*: *Abrarius cribrosus* Fairmaire, 1902: 261 (Madagascar: Plateau de l’Ankara), designation by monotypy; *Entymosina*: *Entymosina parvula* Weise, 1910b: 439 (Madagascar: Nossibé), by present designation.

#### Distribution.

Madagascar (Fig. 278).

#### Ecology.

No information.

#### Notes.

Endemic to Madagascar and comprises about ten known species. The Neotropical genus *Gioia* Bechyné (1955d: 77) is very similar to *Abrarius*, and may well be a synonym.

### Afroaltica

Biondi & D’Alessandro, 2007

http://species-id.net/wiki/Afroaltica

Figs 3 113 –117 279

#### References.

Biondi and D’Alessandro 2007: 99; 2010a: 402; D’Alessandro and Biondi 2011: 365.

#### Type species.

*Afroaltica subaptera* Biondi & D’Alessandro, 2007: 100 (Republic of South Africa, KwaZulu-Natal, Karkloof area), by original designation.

#### Distribution.

Republic of South Africa (KwaZulu-Natal, Limpopo, and Mpumalanga) (Fig. 279).

#### Ecology.

*Afroaltica subaptera* was collected in an open field on Poaceae (also known as Gramineae) (Biondi and D’Alessandro 2007).

#### Notes.

Two species have been described.

### 

**[*Afroalytus* Scherer, 1961]**

= *Manobia* Jacoby, 1885

### Afrocrepis

Bechyné, 1954b

http://species-id.net/wiki/Afrocrepis

Figs 4 118 280

#### References.

Bechyné 1954b: 680; Heikertinger 1925: 99 (as *Derocrepis* Weise, 1886); Biondi and D’Alessandro 2010a: 403.

#### Type species.

*Crepidodera carinipennis* Jacoby, 1903a: 12 (KwaZulu-Natal, Malvern), by original designation.

#### Distribution.

Madagascar (!) [Ambalamanankana (NHMB); Perinet (NHMB); Andohahele (BAQ)] and the Republic of South Africa (Fig. 280).

#### Ecology.

No information.

#### Notes.

Three species are known. *Crepidodera betiokyensis* Bechyné (1954a: 46) from Madagascar, erroneously attributed to this genus by Bechyné (1964: 152), was placed in *Afrorestia* Bechyné (cf. Scherer 1962b: 57; Biondi and D’Alessandro 2010a: 403).However, it is here confirmed that this genus does occur in Madagascar.

### Afrorestia

Bechyné, 1959b

http://species-id.net/wiki/Afrorestia

Figs 5 119 281

#### References.

Bechyné 1959b: 232; Biondi and D’Alessandro 2010a: 403.

#### Type species.

*Crepidodera laeviuscula* Csiki in Heikertinger and Csiki 1940, 1940: 297 (West Africa), by original designation.

#### Distribution.

Burundi, Democratic Republic of the Congo, Ethiopia, Madagascar, Republic of South Africa, Rwanda, Tanzania, Uganda, and Zimbabwe (Fig. 281).

#### Ecology.

Some species of this genus have been collected from plants in the family Apiaceae in South Africa (personal data).

#### Notes.

About twenty described species. *Crepidodera betiokyensis* Bechyné (1954a: 46) from Madagascar, erroneously attributed to *Afrocrepis* Bechyné by Bechyné (1964: 152), was transferred to this genus (cf. Scherer 1962b: 57; Biondi and D’Alessandro 2010a: 403). *Crepidodera sjostedti* Weise (1910a: 221) from Kilimanjaro was incorrectly attributed to *Asiorestia* Jacobson, 1925 (a genus that does not occur in the Afrotropical region) by Bechyné (1957: 181). This species was transferred to *Afrorestia* Bechyné by Biondi and D’Alessandro (2010a: 403).

### 

**[*Allomorpha* Jacoby, 1892b]**

= *Hespera* Weise, 1889

### Alocypha

Weise, 1911

http://species-id.net/wiki/Alocypha

Figs 6 120 282

#### References.

Weise 1911: 170; Biondi and D’Alessandro 2010a: 403.

#### Type species.

*Alocypha litura* Weise, 1911: 171 (East Africa: Lindi) [(= *Aphthona bimaculata* Jacoby, 1903a: 11) (KwaZulu-Natal)], designation by monotypy.

#### Distribution.

Botswana, Malawi, Mozambique, Namibia, Republic of South Africa (KwaZulu-Natal), Tanzania and Zambia (Fig. 282).

#### Ecology.

*Alocypha bimaculata* is a harmful pest of Sesame (*Sesamum indicum* L.) (Pedaliaceae) crops, particularly in Tanzania (Mponda et al. 1997).

#### Notes.

Only one species is known.

### Altica

Geoffroy, 1762

http://species-id.net/wiki/Altica

Figs 7 121 283

=Haltica Illiger, 1801 (unjustified emendation)
=Graptodera Chevrolat, 1836 (synonymized by Weise 1888)

#### References.

Geoffroy 1762: 244; Illiger 1801: 138; Chevrolat 1836: 388; Weise 1888: 825; Allard 1889a: 43 (as *Graptodera*); Bechyné 1954a: 43; 1955a: 209; 1960b: 77; Döberl 2008: 35; Biondi and D’Alessandro 2010a: 403.

#### Type species.

*Altica*: *Chrysomela oleracea* Linnaeus, 1758: 372 (Europe), by subsequent designation by Latreille (1810: 432); *Graptodera*: *Chrysomela oleracea* Linnaeus, 1758: 372 (Europe), by subsequent designation by Chevrolat (1845: 307).

#### Distribution.

All zoogeographical regions (Fig. 283).

#### Ecology.

Polyphagous. This genus has been found associated with herbaceous plants, shrubs and trees belonging to several plant families (cf. Jolivet and Hawkeswood 1995).

#### Notes.

About fifty species are known from Madagascar and Sub-Saharan Africa.

### Amphimela

Chapuis, 1875

http://species-id.net/wiki/Amphimela

Figs 8, 9 122 284

=Cercyonia Weise, 1901 (synonymized by Scherer 1961)
=Diboloides Jacoby, 1897 (synonymized by Scherer 1961)
=Dibolosoma Jacoby, 1897 (synonymized by Biondi and D’Alessandro 2010a)
=Halticella Jacoby, 1899b (name preoccupied by *Halticella*Stephens 1829: 36, Hymenoptera, Chalcidoidea)
=Halticorthaea Csiki in Heikertinger and Csiki 1940 (new name for *Halticella* Jacoby, 1899; synonymized by Scherer 1961)
=Halticova Fairmaire, 1898 (synonymized by Biondi and D’Alessandro 2010a)
=Sphaerophysa Baly, 1876 syn. n.

#### References.

Chapuis 1875: 34; Baly 1875: 27; 1876b: 582; Jacoby 1897: 553, 559; 1899b: 357; Fairmaire 1898: 428; Weise 1901: 303; Maulik 1929: 307 (as *Diboloides*); Csiki in Heikertinger and Csiki 1940: 418; Bechyné 1958c: 10; Scherer 1961: 252; Biondi and D’Alessandro 2010a: 403.

#### Type species.

*Amphimela*: *Amphimela mouhoti* Chapuis, 1875: 36 (Indonesia), designation by monotypy; *Cercyonia*: *Cercyonia variabilis* Weise, 1901: 303 (Tanzania: Bagamojo, Kunow), designation by monotypy; *Diboloides*: *Diboloides bicolor* Jacoby, 1897: 553 (Mashonaland), designation by monotypy; *Dibolosoma*: *Dibolosoma quadripustulatum* Jacoby, 1897: 560 (Madagascar: Diego-Suarez), designation by monotypy; *Halticella*: *Halticella flavopustulata* Jacoby, 1899b: 357 (Natal, Frere), designation by monotypy; *Halticova*: *Halticova rufoguttata* Fairmaire, 1898: 428 (Madagascar), designation by monotypy;* Sphaerophysa*: *Sphaerophysa clavicornis* Baly, 1876b: 582 (Madagascar), designation by monotypy.

#### Distribution.

Afrotropical (including Madagascar), Australian, Eastern Palaearctic and Oriental regions (Fig. 284).

#### Ecology.

*Amphimela bryanti* (Csiki in Heikertinger and Csiki 1940) on *Bersama* sp. (Melianthaceae) in Uganda (Bryant 1936, as *Cercyonia quadrinotata* Bryant 1936: 218); *Amphimela citri* (Bryant 1922: 474) cited as a harmful pest of citrus in West Africa (Bryant 1922a).

#### Notes.

About thirty-five species have been described in the Afrotropical region. Bechyné (1964: 161) established the synonymy between *Dibolosoma quadripustulatum* Jacoby, 1897 [as *4-punctata* (sic!)] and *Halticova rufoguttata* Fairmaire, 1898; while Weise (1910b: 496) established the synonymy between *Sphaerophysa* Baly and *Dibolosoma* Jacoby. Moreover, there are no important diagnostic characters distinguishing *Sphaerophysa* from *Amphimela*, this latter characterized by a wide variability in the Afrotropical region. Therefore, the following new synonymy is proposed: *Amphimela* Chapuis, 1875 = *Sphaerophysa* Baly, 1876b syn. n. Material examined: *Sphaerophysa clavicornis* Baly (det. J. Bechyné), “Madagascar, Tananarive”, 2 specimens (NMPC).

### Anaxerta

Fairmaire, 1902

http://species-id.net/wiki/Anaxerta

Figs 10 285

#### References.

Fairmaire 1902: 267; Biondi and D’Alessandro 2010a: 403.

#### Type species.

*Anaxerta castanea* Fairmaire, 1902: 268 (Madagascar: Ankarahitra), designation by monotypy.

#### Distribution.

Madagascar (Fig. 285).

#### Ecology.

No information.

#### Notes.

A single species has been described.

### Angulaphthona

Bechyné, 1960b

http://species-id.net/wiki/Angulaphthona

Figs 11 123–125 286

#### References.

Bechyné 1960b: 74; Scherer 1978: 265; Gruev 1981: 55; Medvedev 1996: 261.

#### Type species.

*Aphthona heteromorpha* Bechyné, 1955c: 62 (Madagascar: Bas Mangoky), by original designation.

#### Distribution.

Egypt, Tchad, Sudan, Somaliland, Sierra Leone, Nigeria, Democratic Republic of Congo, Uganda, Zambia (!) [50 km W Kasama (BAQ)], Malawi (!) [Dedza (BAQ)], Mozambique, Republic of South Africa (!) [KwaZulu-Natal: Durban (SANC)], Madagascar, and Arabian Peninsula (Saudi Arabia and North Yemen) (Fig. 286).

#### Ecology.

*Angulaphthona heteromorpha* collected on cotton plants, *Gossypium* sp. (Malvaceae) (Bechyné 1955c, as *Aphthona*).

#### Notes.

Five species are known from the Afrotropical region.

### Antanemora

Bechyné, 1964

http://species-id.net/wiki/Antanemora

Figs 12 126–127 287

Lactica Erichson, 1847 (pars)

#### References.

Bechyné 1950: 220 (as *Lactica*); 1964: 145; Bechyné and Bechyné 1975: 26; Biondi and D’Alessandro 2010a: 403.

#### Type species

**.***Lactica carbonaria* Bechyné, 1948a: 7 (Madagascar: Environs de Rogez; Ankazoabo), by original designation.

#### Distribution.

Madagascar (Fig. 287).

#### Ecology.

No information.

#### Notes.

There are about twenty known species (personal data).

### Aphthona

Chevrolat, 1836

http://species-id.net/wiki/Aphthona

Figs 13, 14 128–129 288

=Ethiopia Scherer, 1972 syn. n.
=Pseudeugonotes Jacoby, 1899 (synonymized by Biondi and D’Alessandro 2010a)

#### References.

Chevrolat 1836: 391; Jacoby 1899a: 531; Bechyné 1955c: 62; 1960b: 67, 74; Scherer 1972: 7; 1978: 265 (as *Ethiopia*); Biondi and D’Alessandro 2010a: 404, 407 (as *Ethiopia*).

#### Type species.

*Aphthona*: *Altica cyparissiae* Koch, 1803: 80 (Europe), by subsequent designation by Chûjô (1937: 119); *Ethiopia*: *Ethiopia tricolor* Scherer, 1972: 7 (Ethiopia: Agheresalam), by original designation; *Pseudeugonotes*: *Pseudeugonotes vannutellii* Jacoby, 1899a: 531 (Ethiopia: Sancurar-Amarr Burgi), designation by monotypy.

#### Distribution.

Afrotropical (including Madagascar), Australian, Nearctic, Oriental and Palaearctic regions (Fig. 288). All the species from the Neotropical region described as *Aphthona* should be attributed to different genera (cf. Konstantinov and Vandenberg 1996; Konstantinov 1998).

#### Ecology.

Genus mainly associated with Euphorbiaceae, but also with Geraniaceae, Cistaceae, Rosaceae, Linaceae, Iridaceae, Malvaceae and Lythraceae (cf. Bechyné 1955c; Jolivet and Hawkeswood 1995).

#### Notes.

About one hundred species are described from Madagascar and Sub-Saharan Africa. There are no important diagnostic characters distinguishing *Ethiopia* Scherer from *Aphthona*. Therefore, the following new synonymy is proposed: *Aphthona* Chevrolat, 1836 = *Ethiopia* Scherer, 1972 syn. n. Type material examined: *Ethiopia tricolor* Scherer, holotype ♂ and paratype ♀, “Ethiopia, Agheresalam, 7.6.1963, Linnavuori” (MZHF).

### Argopistes

Motschulsky, 1860

http://species-id.net/wiki/Argopistes

Figs 15 130–132 289

#### References.

Motschulsky 1860: 236; Weise 1895: 335; Bryant 1922a: 474; Biondi and D’Alessandro 2010a: 404.

#### Type species.

*Argopistes biplagiata* Motschulsky, 1860: 236 (Siberia), designation by monotypy.

#### Distribution.

Central, Eastern and Southern Africa, and Madagascar; Australian, Eastern Palaearctic, Nearctic, Northern Neotropical and Oriental regions (Fig. 289).

#### Ecology.

Many species of this genus are associated with Oleaceae in Sub-Saharan Africa, especially with Olive trees [*Olea europaea* var. *africana* (Mill.)], on which the larvae are leaf miners and adults defoliators (cf. Jolivet and Hawkeswood 1995; personal data).

#### Notes.

About fifteen species recorded from Madagascar and Sub-Saharan Africa (personal data).

### Argopistoides

Jacoby, 1892b

http://species-id.net/wiki/Argopistoides

Figs 16 133 290

=Torodera Weise, 1902a (synonymized by Medvedev 2009)

#### References.

Jacoby 1892b: 932; Weise 1902a: 163; Scherer 1987: 67 (as *Torodera*); Biondi 1994 (as *Torodera*): 437; Medvedev 2009: 40; Biondi and D’Alessandro 2010a: 404 (as *Torodera*).

#### Type species.

*Argopistoides septempunctata* Jacoby, 1892b: 932 [Burma (=Myanmar): Carin Chebà], designation by monotypy; *Torodera*: *Torodera octomaculata* Weise, 1902a: 164 (Kwai), by subsequent designation by Scherer (1987:67).

#### Distribution.

Republic of the Congo, Democratic Republic of the Congo, Guinea, Kenya, Rwanda (!) [Nyungwe National Park, Kamiranzovu (BAQ)], Republic of South Africa (KwaZulu-Natal), Sierra Leone, Sudan, Tanzania, Uganda, Zimbabwe, and Oriental region (Fig. 290).

#### Ecology.

Genus reported from Poaceae (also known as Gramineae) (*Oryza*) in Kenya [cf. Jolivet and Hawkeswood 1995 (as *Torodera*)].

#### Notes.

The Afrotropical region has four described species.

### 

**[*Argopus* Fischer, 1824]**

Not present in the Afrotropical region.

**References.** Fischer 1824: 184; Weise 1902a: 171; Laboissière 1941: 319.

**Notes.** *Argopus maculiceps* Boheman (1859: 200) was transferred to the genus *Toxaria* Weise by Laboissière (1941); *Argopus pusillus* Gerstaecker (1871: 85) was transferred to the genus *Sphaeroderma* Stephens by Weise (1902a).

**[*Argosomus* Wollaston, 1867]**

= *Sphaeroderma* Stephens, 1831

**[*Aridohespera* Selman, 1963]**

= *Eriotica* Harold, 1877a

**[*Asiorestia* Jacobson, 1925]**

Not present in the Afrotropical region.

**References.** Jacobson 1925: 274; Bechyné 1957: 181; 1959b: 233.

**Notes.** Bechyné (1957) attributed *Crepidodera sjostedti* Weise (1910a: 221) from Kilimanjaro to this genus. This species was recently transferred to *Afrorestia* Bechyné by Biondi and D’Alessandro (2010a: 404).

**[*Asphaera* Chevrolat, 1843]**

Not present in the Afrotropical region.

**References.** Chevrolat 1843: 227; Fairmaire 1886: 93; Jacoby 1892a: 573; Bechyné 1959c: 319.

**Notes.** Three species wrongly attributed to this Neotropical genus were transferred to the genera *Physomandroya* Bechyné and *Hemipyxis* Chevrolat by Bechyné (1959c).

### Bangalaltica

Bechyné, 1960a

http://species-id.net/wiki/Bangalaltica

Figs 17 134 291

#### References.

Bechyné 1960a: 9; Biondi and D’Alessandro 2003: 104; 2010a: 404.

#### Type species.

*Bangalaltica antennalis* Bechyné, 1960a: 9 (Democratic Republic of the Congo: Bangala; Lubutu-Kituri), designation by monotypy.

#### Distribution.

Democratic Republic of the Congo and Republic of the Congo (Fig. 291).

#### Ecology.

No information.

#### Notes.

One species is known.

### 

**[*Balanomorpha* Chevrolat, 1836]**

= *Mantura* Stephens, 1831

### Bechuana

Scherer, 1970

http://species-id.net/wiki/Bechuana

Figs 18 135 292

#### References.

Scherer 1970: 301; Biondi and D’Alessandro 2010a: 404.

#### Type species.

*Bechuana nigripes* Scherer, 1970: 302 (Free State: Boshof; North-West Province: Vryburg), by original designation.

#### Distribution.

Republic of South Africa (Eastern Cape Province, Free State, Gauteng, KwaZulu-Natal, North-West Province and Western Cape Provinces) (Fig. 292).

#### Ecology.

No information.

#### Notes.

There are two known species. *Ochrosis natalensis* Jacoby (1906: 17) was attributed to this genus by Biondi and D’Alessandro (2010a).

### Bechynella

Biondi & D’Alessandro, 2010

http://species-id.net/wiki/Bechynella

Figs 19 136–137 293

Serraphula Jacoby, 1897 (pars)

#### References.

Biondi and D’Alessandro 2010b: 28; Bechyné 1955b: 517 (as *Serraphula*); 1959a: 13 (as *Serraphula*).

#### Type species.

*Serraphula bohumilae* Bechyné, 1955b: 517 (French Guinea: Dalaba), by original designation.

#### Distribution.

Cameroon, Democratic Republic of the Congo, Guinea, Ivory Coast and Nigeria (Fig. 293).

#### Ecology.

No information.

#### Notes.

The three species attributed to this genus were described by Bechyné (1955b, 1959a) as *Serraphula* Jacoby (Biondi and D’Alessandro 2010b).

### Bezdekaltica

Döberl, 2012

http://species-id.net/wiki/Bezdekaltica

Figs 20 138 294

#### References.

Döberl 2012: 434.

#### Type species.

*Bezdekaltica socotrana*Döberl, 2012: 435 (Socotra Island), by original designation.

#### Distribution.

Yemen (Socotra Island) (Fig. 294).

#### Ecology.

Collected in November in *Dracaena* (Dracenaceae) Forest.

#### Notes.

Only one species has been described.

### Bikasha

Maulik, 1931

http://species-id.net/wiki/Bikasha

Figs 21 139 –140 295

#### References.

Maulik 1931: 256; Konstantinov and Prathapan 2008: 387; Biondi and D’Alessandro 2010a: 404.

#### Type species.

*Bikasha tenuipunctata* Maulik, 1931: 257 (Seychelles), by original designation.

#### Distribution.

Burundi (!) [Kibira National Park (BAQ)], Democratic Republic of the Congo (!) [Kivu, Mbingi (BAQ)], Kenya (!) [Mt. Kenya (BAQ); Kikuyu (BAQ)], Madagascar (!) [NW of Ranomafana (ZMHB); Andasibe (BAQ)], Malawi (!) [Dedza (BAQ)], Republic of South Africa (!) (KwaZulu-Natal, 17 km NE of Empangeni (MZLU)], Rwanda (!) [Nyungwe National Park, Pindura (BAQ)]; Seychelles, Sierra Leone (!) [Bumbuna (BAQ); S of Freetown (MZLU)], Eastern Palaearctic and Oriental regions (Fig. 295).

#### Ecology.

In Seychelles, *Bikasha tenuipunctata* Maulik and *Bikasha fortipunctata* Maulik (1931: 258) were collected in forest, and *Bikasha minor* Maulik (1931: 259) in wet coastal meadows.

#### Notes.

About ten species are known from the Afrotropical region (personal data).

### Biodontocnema

Biondi, 2000 stat. prom.

http://species-id.net/wiki/Biodontocnema

Figs 22 141–145 296

#### References.

Biondi 2000: 347; 2002b: 356; Biondi and D’Alessandro 2010a: 404; Konstantinov et al. 2011: 19.

#### Type species.

*Biodontocnema brunnea* Biondi, 2000: 348 (Namibia, Kaross), designation by monotypy.

#### Distribution.

Namibia (Fig. 296).

#### Ecology.

*Biodontocnema brunnea* is the only species in this genus, and it is associated with moist habitats (Biondi 2000).

#### Notes.

One species has been described. This genus was erroneously synonymized with *Chaetocnema* Stephens by Konstantinov et al. (2011). These authors did not examine material of *Biodontocnema* and based their synonymy only on the examination of some photos and figures. *Chaetocnema schlaeflii* (Stierlin 1866: 31) and *Chaetocnema major* (Jacquelin du Val 1852: 717), display an apparently bidentate dorsal tibial margin, which differs from the bidentate dorsal tibial margin of *Biodontocnema* (Fig. 144). These two species are easily distinguishable from this Namibian genus by having: hind tibial socket narrow with no dentiform inner margin [hind tibial socket wide with dentiform inner margin in *Biodontocnema* (Figs 142–143)]; ratio of metasternal width/metasternal length ≤ 2.50 [> 2.50 in *Biodontocnema* (Fig. 145)]; first metatarsomere not laterally compressed [laterally compressed in *Biodontocnema* (Fig. 142)]; antennomeres 6–10 distinctly longer than wide [antennomeres 6–10 about as long as wide in *Biodontocnema* (Figs 22)]; distinctly larger size (LB > 3.00 mm) ( LB < 2.5 mm in *Biodontocnema*); longer antennae that reach at least the middle of the elytra [antennae shorter reaching only base of elytra in *Biodontocnema* (Fig. 22)]; Moreover, *Biodontocnema* is phylogenetically more closely related to *Carcharodis* Weise than to *Chaetocnema* (Biondi 2002b). On the basis of these considerations, we regard *Biodontocnema* Biondi, 2000 as a valid genus.

### Blepharida

Chevrolat, 1836

http://species-id.net/wiki/Blepharida

Figs 23 146–149 297

= Blepharidella Weise, 1910a (synonymized by Scherer 1961)
= Blepharidula Weise, 1916 (an unnecessary new name for *Eutheca* Baly 1878)
= Calotheca Heyden, 1887(new name for *Eutheca* Baly, 1878; synonymized by Scherer 1961)
= Eutheca Baly, 1878c (name preoccupied by *Eutheca* Kiesenwetter in Erichson 1877: 155, Coleoptera: Anobiidae)
subgen. Blepharidina Bechyné, 1968

#### References.

Chevrolat 1836: 394; Baly 1878c: 204; Heyden 1887: 98; Duvivier 1891: 242; Weise 1910a: 220; 1916: 39; Laboissière 1942: 95; Bryant 1944a: 129; Scherer 1961: 252; Bechyné 1968: 1725; Furth 1998: 12; Furth and Lee 2000: 26; Becerra 2004: 116; Biondi and D’Alessandro 2010a: 404.

#### Type species.

*Blepharida*: *Chrysomela rhois* Forster, 1771: 21 (North America), by subsequent designation by Chevrolat (1842: 606); *Blepharidella*: *Blepharidella sjostedti* Weise, 1910a: 219 (Kilimanjaro, Kibonoto), by subsequent designation by Furth (1998: 12); *Blepharidina*: *Blepharida guttulata* Baly, 1881: 52 (Angola), by original designation; *Eutheca*: *Eutheca haroldi* Baly, 1878c: 205 (Lake Nyassa), designation by monotypy.

#### Distribution.

Afrotropical (excluding Madagascar), Nearctic, Neotropical and Southern Palaearctic (Egypt, Israel, and Saudi Arabia) regions (Fig. 297).

#### Ecology.

The Afrotropical species of *Blepharida* are generally associated with shrubs of *Rhus* (Anacardiaceae) (Furth and Young 1988: 496; Prathapan and Chaboo 2011: 97–105; personal data).

#### Notes.

Sub-Saharan Africa has about thirty-five described species. Two species from Madagascar previously attributed to this genus, *Blepharida multiguttata* Duvivier (1891: 242) and *Blepharida insignis* Brancsik (1897: 130), are transferred to *Xanthophysca* Fairmaire. The nominotypical subgenus of *Blepharida* is mainly widespread in Nearctic and Neotropical regions, whereas the subgenus *Blepharidina* Bechyné is restricted to the Afrotropical region (Furth 1998).

### 

**[*Blepharidina* Bechyné, 1968]**

subgenus of *Blepharida* Chevrolat, 1836

**[*Blepharidula* Weise, 1916]**

= *Blepharida* Chevrolat, 1836

**References.** Weise 1916: 39; Baly 1878c: 204; Heyden 1887: 98.

**Notes.** An unnecessary new name proposed by Weise (1916) for *Eutheca* Baly, 1878 (nec *Eutheca* Kiesenwetter in Erichson 1877: 155, Coleoptera: Anobiidae). Weise (1916) incorrectly regarded the previous replacement name for *Eutheca* (*Calotheca* Heyden, 1887) as unavailable because it was already in use for a plant genus of Poaceae (also known as Gramineae) (cf. Palisot de Beauvois 1812: 85).

**[*Brinckaltica* Bechyné, 1959b]**

= *Chaetocnema* Stephens, 1831

**[*Buphonella* Jacoby, 1903a]**

Fig. 24

The genus *Buphonella*(Fig. 24) is here transferred to the tribe Galerucini.

**References.** Jacoby 1903a: 37; Laboissière 1922: 178; Biondi and D’Alessandro 2010a: 405.

**Type species.***Apophylia murina* Gerstaecker, 1871: 83 (Zanzibar), by subsequent designation by Laboissière (1922: 179).

**Distribution.** Central, Eastern andSouthernAfrica.

**Ecology.** Associated with Poaceae (also known as Gramineae). *Buphonella murina* (Gerstaecker) and *Buphonella nigroviolacea* (Allard, 1889b: LXXI, LXXIV) can damage maize (cf. Drinkwater 1989: 315).

**Notes.** Four species have been described. Furth and Suzuki (1994: 123, Fig. 10) report a metafemoral spring for this genus, including a drawing, but re-examination of the specimens they examined revealed that they belong to the genus *Hespera* Weise. Dissection of a variety of *Buphonella* specimens did not reveal any structure comparable to a metafemoral spring either. We therefore transfer the genus *Buphonella* Jacoby to the tribe Galerucini.

**[*Calotheca* Heyden, 1887]**

= *Blepharida* Chevrolat, 1836

### Carcharodis

Weise, 1910b

http://species-id.net/wiki/Carcharodis

Figs 25 150 298

#### References.

Weise 1910b: 434; Bechyné 1954b: 683; Biondi 2002b: 356; Biondi and D’Alessandro 2010a: 405.

#### Type species.

*Chaetocnema rugiceps* Baly, 1877: 308 (Madagascar), by subsequent designation by Bechyné (1954b: 683).

#### Distribution.

Central and Southern Africa, and Madagascar (Fig. 298).

#### Ecology.

Species of this genus live in moist habitats and are probably associated with plants of the family Cyperaceae (personal data).

#### Notes.

There are seven known species.

### Celisaltica

Biondi, 2001a

http://species-id.net/wiki/Celisaltica

Figs 26 151 –152 299

#### References.

Biondi 2001a: 644; Biondi and D’Alessandro 2010a: 405.

#### Type species.

*Celisaltica ruwenzorica* Biondi, 2001a: 644 (Ruwenzori), by original designation.

#### Distribution.

Uganda (Fig. 299).

#### Ecology.

This is the only species in this genus. It lives at high altitudes (3200–4000 m) on the Ruwenzori Massif, and is associated with the *Ericetum* (Ericaceae) plant community (Biondi 2001a).

#### Notes.

One species.

### 

**[*Cercyonia* Weise, 1901]**

= *Amphimela* Chapuis, 1875

### Chabria

Jacoby, 1887

http://species-id.net/wiki/Chabria

Figs 27 153 300

#### References.

Jacoby 1887: 92; Biondi and D’Alessandro 2012a: 3.

#### Type species.

*Chabria nigroplagiata* Jacoby, 1887: 92 (Sri Lanka: Bogawantalawa), by subsequent designation by Maulik (1926: 312).

#### Distribution.

Cameroon (probably introduced); Madagascar, and Republic of South Africa (KwaZulu-Natal) (probably introduced), Australian and Oriental regions; (Fig. 300).

#### Ecology.

There is no information for the Afrotropical region. *Chabria* species were collected on *Melastoma* (Melastomataceae) in Malaysia (Jolivet and Hawkeswood 1995), in cacao plantations [*Theobroma cacao* L. (Malvaceae)] in Sulawesi (Indonesia) (Medvedev 2008) and in primary forest in Mindanao (Philippines) (Medvedev 2002).

#### Notes.

Four species are known from Afrotropical region, one from Sub-Saharan Africa, probably introduced, and three from Madagascar.

### Chaetocnema

Stephens, 1831

http://species-id.net/wiki/Chaetocnema

Figs 28 154 301

=Brinckaltica Bechyné, 1959b (synonymized by Scherer 1961)
=Exorhina Weise, 1886 (synonymized by Heikertinger and Csiki 1940)
=Plectroscelis Chevrolat, 1836 (synonymized by Weise 1886)

#### References.

Stephens 1831: 325; Chevrolat 1836: 393; Baly 1877: 166; Weise 1886: 750; Bryant 1928: 393; Heikertinger and Csiki 1940: 376; Laboissière 1942: 81; Bechyné 1959b: 236; 1960b: 91; Scherer 1961: 259; Biondi 2001b: 233; 2002a: 266; 2002b: 356; Biondi and D’Alessandro 2006: 720; 2010a: 405.

#### Type species.

*Chaetocnema*: *Chrysomela concinna* Marsham, 1802 (Europe), by subsequent designation by Westwood (1840: 42); *Exorhina*: *Haltica chlorophana* Duftschmid, 1825: 286 (Austria), by subsequent designation by Döberl (2010: 508); *Brinckaltica*: *Chaetocnema subaterrima* Jacoby, 1900: 254 (Natal), by original designation.

#### Distribution.

All zoogeographical regions (Fig. 301).

#### Ecology.

This genus is mainly associated with plants in the families Chenopodiaceae, Cyperaceae, Juncaceae, Poaceae (also known as Gramineae), and Polygonaceae (cf. Jolivet and Hawkeswood 1995). In the Afrotropical region some *Chaetocnema* are serious pests of rice (Biondi and D’Alessandro 2008a).

#### Notes.

Over one hundred species are known from Madagascar and Sub-Saharan Africa.

### Chaillucola

Bechyné, 1968

http://species-id.net/wiki/Chaillucola

Figs 29 155 302

#### References.

Bechyné 1968: 1713; Biondi and D’Alessandro 2003: 104; 2010a: 405.

#### Type species.

*Chaillucola formicicornis* Bechyné 1968: 1714 (Republic of Congo: Mbila), by original designation.

#### Distribution.

Cameroon (!) [N’Kongsamba (MCSN)] and Republic of Congo (Fig. 302).

#### Ecology.

No information.

#### Notes.

A single species has been described.

### 

**[*Chaloenus* Westwood, 1862]**

Not present in the Afrotropical region.

**References.** Westwood 1862: 216; Bryant 1927: 615; Bechyné 1955b: 543.

**Notes.** *Terpnochlorus perrieri* Fairmaire, 1904 (= *Chaloenus viridis* Bryant, 1927); this species was incorrectly attributed to the Oriental genus *Chaloenus*(Bechyné, 1955b).

### Chirodica

Germar, 1834

http://species-id.net/wiki/Chirodica

Figs 30 156–157 303

#### References.

Germar 1834: 2; Baly 1876a: 441; Scherer 1983: 173; Biondi 1998b: 17, 45; Biondi and D’Alessandro 2010a: 406.

#### Type species.

*Chirodica chalcoptera* Germar, 1834: 16 (Cape of Good Hope), designation by monotypy.

#### Distribution.

Namibia and the Republic of South Africa (Fig. 303).

#### Ecology.

This genus is strictly associated with the plant family Proteaceae (mainly *Protea* spp. and *Leucadendron* spp.) (Biondi 1998b).

#### Notes.

Eight species have been described.

### 

**[*Cladocera* Hope, 1840]**

= *Polyclada* Chevrolat, 1836

**[*Cladotelia* Kolbe, 1894]**

= *Polyclada* Chevrolat, 1836

### Collartaltica

Bechyné, 1959a

http://species-id.net/wiki/Collartaltica

Figs 31 158–160 304)

#### References.

Bechyné 1959a: 27; Biondi 2002b: 358; Biondi and D’Alessandro 2004: 286; 2010a: 406.

#### Type species.

*Collartaltica cryptostoma* Bechyné, 1959a: 27 (Democratic Republic of the Congo: Faradje, Tomati), designation by monotypy.

#### Distribution.

Central African Republic, Democratic Republic of the Congo, Kenya, Nigeria, Republic of South Africa, Sudan, Tanzania and Zimbabwe (Fig. 304).

#### Ecology.

This genus is associated with Poaceae (also known as Gramineae) in moist meadows and forest clearings (Biondi and D’Alessandro 2004).

#### Notes.

There are six known species.

### 

**[*Crepidodera* Chevrolat, 1836]**

Not present in the Afrotropical region.

**References.** Chevrolat 1836: 391.

**Notes.** Afrotropical species initially attributed to this genus (cf. Bryant 1927; Jacoby 1899b, 1903a, 1906, Weise 1902b, 1910a, 1924) have mostly been transferred to the genera *Afrorestia* Bechyné, *Afrocrepis* Bechyné and *Orthocrepis* Weise, 1888.

**[*Cyrtoma* Clark, 1863]**

Nomen nudum.

**Notes.** Clark (1863: 165) uses this genus-name, without any description, for an unpublished species from Madagascar [“apicale, *Clark*” synonymized with “4-maculata, *Chevr*.” and “Madecassae, *Chevr*.” (the latter two were also never published)].

### Decaria

Weise, 1895

http://species-id.net/wiki/Decaria

Figs 32 305

=Embolimus Weise, 1902

#### References.

Weise 1895: 344; 1902b: 303; Biondi and D’Alessandro 2003: 104; 2010a: 406.

#### Type species.

*Decaria*: *Decaria tricolor* Weise, 1895: 344 (Sierra Leone, Bang-Haas), designation by monotypy; *Embolimus*: *Embolimus pauli* Weise, 1902b: 304 (Kwai), designation by monotypy.

#### Distribution.

Afrotropical region (excluding Madagascar) (Fig. 305).

#### Ecology.

*Decaria* is associated mainly with plans from the genera *Heliotropium* (Boraginaceae), *Cola* (Sterculiaceae) and *Ocimum* (Lamiaceae) (cf. Jolivet and Hawkeswood 1995).

#### Notes.

About twenty species have been described.

### 

**[*Decarthrocera* Laboissière, 1937]**

**References.** Laboissière 1937: 27; Furth and Suzuki 1994: 131.

**Notes.** This genus was transferred to the subfamily Galerucinae (currently tribe Galerucini) by Furth and Suzuki (1994).

**[*Derocrepis* Weise, 1886]**

Not present in the Afrotropical region.

**References.** Weise 1886: 676, 686; Heikertinger 1925: 95.

**Notes.** Species initially attributed to this Palaearctic genus were transferred to the genera *Afrocrepis* Bechyné and *Afrorestia* Bechyné.

### Diamphidia

Gerstaecker, 1855

http://species-id.net/wiki/Diamphidia

Figs 33 161–163 306

#### References.

Gerstaecker 1855: 638; Baly 1861: 198; Laboissière 1942: 109; Biondi and D’Alessandro 2010a: 406.

#### Type species.

*Diamphidia femoralis* Gerstaecker, 1855: 638 (Sena and Port Natal), designation by monotypy.

#### Distribution.

Central, Eastern, and Southern-Eastern Africa (Fig. 306).

#### Ecology.

A genus associated with shrubs and trees of *Commiphora* (Burseraceae) (cf. Jolivet and Hawkeswood 1995; Chaboo et al. 2007).

#### Notes.

Seventeen species are known.

### Dibolia

Latreille, 1829

http://species-id.net/wiki/Dibolia

Figs 34 164–166 307

=Haltitarsus Berthold in Latreille 1827 (synonymy reported by Heikertinger and Csiki 1940)

#### References.

Latreille 1829: 155; Berthold in Latreille 1827: 401; Baly 1876b: 598; Weise 1926: 24 (as *Haltitarsus*); Heikertinger and Csiki 1940: 485; Bechyné 1960b: 101; Biondi and D’Alessandro 2010a: 406.

#### Type species.

*Dibolia*: *Haltica occultans* Koch, 1803: 22 (Europe), by subsequent designation by Chûjô (1936: 84).

#### Distribution.

Sub-Saharan Africa (absent in Madagascar); Nearctic, Neotropical and Palaearctic regions (Fig. 307).

#### Ecology.

A genus associated mainly with plants in the family Lamiaceae, but also with plants in the Scrophulariceae, Asteraceae and Apicaeae (cf. Jolivet and Hawkeswood 1995).

#### Notes.

About twenty species in Sub-Saharan Africa.

### 

**[*Diboloides* Jacoby, 1897]**

= *Amphimela* Chapuis, 1875

**[*Dibolosoma* Jacoby, 1897]**

= *Amphimela* Chapuis, 1875

### Dimonikaea

Bechyné, 1968

http://species-id.net/wiki/Dimonikaea

Figs 35 167–169 308

#### References.

Bechyné 1968: 1711; Biondi and D’Alessandro 2003: 104; 2010a: 406.

#### Type species.

*Dimonikaea descarpentriesi* Bechyné, 1968: 1712 (Republic of Congo: Dimonika), by original designation.

#### Distribution.

Republic of Congo and Gabon (!) [Makokou (NMHN)] (Fig. 308).

#### Ecology.

No information.

#### Notes.

A single species is known. Bechyné (1968) incorrectly included *Gabonia miraculosa* Scherer (1963: 652) in this genus (Biondi and D’Alessandro 2010a).

### Diphaulacosoma

Jacoby, 1892a

http://species-id.net/wiki/Diphaulacosoma

Figs 36 170–171 309

=Neoderina Bechyné, 1952 (subgenus of *Neodera* Duvivier, 1891) syn. n.

#### References.

Jacoby 1892a: 574; Bechyné 1952: 251; Biondi and D’Alessandro 2010a: 406.

#### Type species.

*Diphaulacosoma***:** *Diphaulacosoma laevipenne* Jacoby, 1892a: 574−575 (Madagascar), designation by monotypy; *Neoderina*: *Neodera (Neoderina) crassicornis* Bechyné, 1952: 251 (Madagascar, Ambohitsitondrona), designation by monotypy.

#### Distribution.

Madagascar (Fig. 309).

#### Ecology.

No information.

#### Notes.

Four species (personal data). No diagnostic characters distinguish *Neoderina* Bechyné from *Diphaulacosoma*. Therefore, the following new synonymy is proposed: *Diphaulacosoma* Jacoby, 1892a = *Neoderina* Bechyné, 1952 syn. n. Material examined: *Neodera (Neoderina) crassicornis* Bechyné, “Madagascar, Ambohitsitondrona, x-xii.47, Michel leg.”, “typus” ♂ and “cotypus” ♀ (MNHN).

### Djallonia

Bechyné, 1955b

http://species-id.net/wiki/Djallonia

Figs 37 172 310

#### References.

Bechyné 1955b: 534; Scherer 1962a: 57; Biondi and D’Alessandro 2010a: 406.

#### Type species.

*Djallonia maindra* Bechyné, 1955b: 534 (French Guinea: Dalaba), designation by monotypy.

#### Distribution.

Democratic Republic of the Congo and Guinea (Fig. 310).

#### Ecology.

No information.

#### Notes.

Only one species is known.

### Drakensbergianella

Biondi & D’Alessandro, 2003

http://species-id.net/wiki/Drakensbergianella

Figs 38 173–175 311

#### References.

Biondi and D’Alessandro 2003: 100; 2010a: 406.

#### Type species.

*Drakensbergianella rudebecki* Biondi and D’Alessandro, 2003: 100 (Republic of South Africa, Drakensberg), designation by monotypy.

**Distribution.** Republic of South Africa (Free State and KwaZulu-Natal) (Fig. 311).

#### Ecology.

*Drakensbergianella rudebecki* is the only species in this genus. It lives in alpine meadows (> 2,000 m) on the Drakensberg and was collected on the inflorescences of *Senecio* and *Helichrysum* (Asteraceae) (Biondi and D’Alessandro 2003).

#### Notes.

A single species has been described.

### Dunbrodya

Jacoby, 1906

http://species-id.net/wiki/Dunbrodya

Figs 39 176–179 312

#### References.

Jacoby 1906: 19; Biondi and D’Alessandro 2010a: 407.

#### Type species.

*Dunbrodya nitida* Jacoby, 1906: 20 (Cape Colony), designation by monotypy.

#### Distribution.

Ethiopia (!) [Sidamo (BAQ)] and the Republic of South Africa [Eastern (!): Kirkwood (SANC), Grahamstown (SANC), Uitenhage (BAQ); Northern (!): Port Nolloth (SANC); and Western Cape Provinces] (Fig. 312).

#### Ecology.

*Dunbrodya nitida* was collected on an *Asparagus* sp. (Asparagaceae) (Jacoby 1906).

#### Notes.

Two species are known (personal data).

### 

**[*Embolimus* Weise, 1902b]**

= *Decaria* Weise, 1895

**[*Entymosina* Weise, 1910b]**

= *Abrarius* Fairmaire, 1902

### Epitrix

Foudras, 1860

http://species-id.net/wiki/Epitrix

Figs 40 180 313

=Euplecnema Jacoby, 1906 (synonymized by Scherer 1963)

#### References.

Foudras 1860: 147; Jacoby 1906: 22; Scherer 1963: 672; Biondi and D’Alessandro 2010a: 407.

#### Type species.

*Epitrix*: *Epitrix atropae* Foudras, 1860: 55 (Europe), by subsequent designation by Maulik (1926: 130, 133); *Euplecnema*: *Euplecnema nigrita* Jacoby, 1906: 22 (Dunbrody, Cape Colony), designation by monotypy.

#### Distribution.

All zoogeographical regions (Fig. 313).

#### Ecology.

The genus *Epitrix* is mainly associated with plants in the family Solanaceae. Some species can be harmful to plants of economic importance (cf. Jolivet and Hawkeswood 1995).

#### Notes.

About a dozen species are known from Madagascar and Sub-Saharan Africa.

### 

**[*Eremiella* Weise, 1910a]**

= *Eurylegna* Weise, 1910a

### Eriotica

Harold, 1877a

http://species-id.net/wiki/Eriotica

Figs 41 181 314

=Aridohespera Selman, 1963 (synonymized by Selman 1968)
=Niphraea Baly, 1878a (synonymized by Harold 1878)

#### References.

Harold 1877a: 107; 1878: 206; Baly 1878a: 40; Ferreira 1963: 516; Selman 1963: 1156; 1968: 248; Biondi and D’Alessandro 2010a: 407.

#### Type species.

*Eriotica*: *Eriotica fuscipennis* Harold, 1877a: 107 (Nyassa), designation by monotypy; *Aridohespera*: *Aridohespera mateui* Selman, 1963: 1157 (Terr. N. Tchad, Ouedi Saala, Mortcha), by original designation. *Niphraea*: *Niphraea hirtipennis* Baly, 1878a (Lake Nyassa), designation by monotypy.

#### Distribution.

Ethiopia, Kenya (!) [S of Garissa (BAQ); NW of Garsen (BAQ); Taita, Mwatate (BAQ)], Malawi, Mozambique, Tanzania and Socotra Island (Yemen) (Fig. 314).

#### Ecology.

No information.

#### Notes.

Seven species are known.

### 

**[*Escaleriella* Weise, 1907a]**

= *Lypnea* Baly, 1876a

**[*Ethiopia* Scherer, 1972]**

= *Aphthona* Chevrolat, 1836

**[*Eugonotes* Jacoby, 1897]**

= *Sanckia* Duvivier, 1891

**[*Euplecnema* Jacoby, 1906]**

= *Epitrix* Foudras, 1860

### Eurylegna

Weise, 1910a

http://species-id.net/wiki/Eurylegna

Figs 42, 43 182 315

=Eremiella Weise, 1910a (synonymized by Scherer 1972)
=Eurylegniella Scherer, 1972 syn. n.

#### References.

Weise 1910a: 228; Scherer 1972: 10, 12; Biondi and D’Alessandro 2010a: 407.

#### Type species.

*Eurylegna*: *Eurylegna fulva* Weise, 1910a: 228 (Kilimanjaro), designation by monotypy; *Eremiella*: *Eremiella rubra* Weise, 1910a: 229 (Kilimanjaro in Kiboscho, 3.000 m), designation by monotypy; *Eurylegniella*: *Eurylegna guineensis* Bechyné, 1955b: 528 (French Guinea: Mount Gangan; Dalaba; Nzérékoré; Mount Nimba), by original designation.

#### Distribution.

Nigeria, Guinea, Ethiopia, Democratic Republic of the Congo, Uganda (!) [Budongo Forest, Sonso (BAQ)], Rwanda, and Malawi (!) [Dedza (BAQ)] (Fig. 315).

#### Ecology.

No information.

#### Notes.

Six species have been described. There are no important diagnostic characters distinguishing *Eurylegniella* Scherer from *Eurylegna*. The following synonymy is therefore proposed: *Eurylegna* Weise, 1910 = *Eurylegniella* Scherer, 1972 syn. n. Material examined: *Eurylegniella guineensis* (Bechyné) (det. G. Scherer), “Imperial College, Expdn. Ghana 1960, 24.8.60, Bobiri Forest, Kumasi, Ashanti”, 1 specimen (ZSM); “Congo Belge, P.N.G., Miss. H. De Saeger, Mt Embe, 20-iv-1952, H. De Saeger, 3347”, 1 specimen (MRAC).

### 

**[*Eurylegniella* Scherer, 1972]**

= *Eurylegna* Weise, 1910a

**[*Eutheca* Baly, 1878c]**

= *Blepharida* Chevrolat, 1836

### Eutornus

Clark, 1860

http://species-id.net/wiki/Eutornus

Figs 44 183–184 316

Oedionychus Berthold, 1827 (pars)

#### References.

Clark 1860: 64; Bechyné 1959c: Biondi and D’Alessandro 2010a: 407.

#### Type species.

*Oedionychus (Eutornus) africanus* Clark, 1860: 65, by original designation.

#### Distribution.

Madagascar and Sub-Saharan Africa (absent in the northern-eastern part of EAF) (Fig. 316).

#### Ecology.

No information.

#### Notes.

About eight species are known, one of which is from Madagascar.

### 

**[*Exorhina* Weise, 1886]**

= *Chaetocnema* Stephens, 1831

### Gabonia

Jacoby, 1893

http://species-id.net/wiki/Gabonia

Figs 45 185–186 317

=Jamesonia Jacoby, 1895 (unnecessary new name for *Gabonia* Jacoby; synonymized by Weise 1902a)
=Orneates Jacoby, 1899b (synonymized by Weise 1910a)
=Thrymnes Weise, 1895 (synonymized by Weise 1902a)

#### References.

Jacoby 1893: 101; 1895: 341; 1899b: 345; Weise 1895: 338; 1902a: 173; 1910a: 231; Bechyné 1955b: 489; Scherer 1962a: 21; Scherer and Boppré 1997: 10, 32; Biondi and D’Alessandro 2003: 105; 2010a: 407.

#### Type species.

*Gabonia*: *Gabonia unicostata* Jacoby, 1893: 101 (Gabon), designation by monotypy; *Orneates*: *Orneates nigritus* Jacoby, 1899b: 345 (Natal), designation by monotypy; *Thrymnes*: *Thrymnes custos* Weise, 1895: 339 (Ashante), by present designation.

#### Distribution.

Afrotropical region (excluding Madagascar) and Arabian Peninsula (?) (Fig. 317).

#### Ecology.

Polyphagous. This genus has been associated with several plant families (cf. Jolivet and Hawkeswood 1995; personal data).

#### Notes.

About one hundred and fifty species are known to occur in Sub-Saharan Africa. According to Biondi and D’Alessandro (2003), *Gabonia* is closely related to *Luperomorpha* Weise, 1887. The latter genus is widespread and prevalent in the Australian and Oriental regions, and is probably a synonym of the former genus. Many species currently attributed to *Gabonia* do not show any significant differences from *Luperomorpha* species. The diagnostic character reported by Scherer and Boppré (1997) for separating these two genera is the apical spur on the hind tibia: long and straight in *Gabonia*; very short in *Luperomorpha*. However, this character is not always reliable. In view of the wide spectrum of variability displayed by the genus *Gabonia*, and the need to consider various other genera, synonymy with *Luperomorpha* may be confirmed by a detailed and careful comparative study of this complicated African genus in the future (Biondi and D’Alessandro 2010a; Doguet 1979).

### 

**[*Gastrida* Chapuis, 1879]**

**References.** Chapuis, 1879: 20; Furth and Suzuki 1994: 131.

**Notes.** This genus was transferred to the subfamily Galerucinae (currently tribe Galerucini) by Furth and Suzuki (1994).

**[*Graptodera* Chevrolat, 1836]**

= *Altica* Geoffroy, 1762

### Guilielmia

Weise, 1924

http://species-id.net/wiki/Guilielmia

Figs 46 318

#### References.

Weise 1924: 23; Scherer 1961: 269; Biondi and D’Alessandro 2003: 97; 2010a: 408.

#### Type species.

*Guilielmia monticola* Weise, 1924: 24 (Birunga, Mount Mukeno), designation by monotypy.

#### Distribution.

Uganda and Rwanda (Fig. 318).

#### Ecology.

The only species in this genus was collected at a high altitude (3100 m) (Weise 1924).

#### Notes.

A single species is known.

### Guinerestia

Scherer, 1959

http://species-id.net/wiki/Guinerestia

Figs 47 187 –188 319

#### References.

Scherer 1959: 243−244; 1962a: 57; Biondi and D’Alessandro 2010a: 408.

#### Type species.

*Guinerestia rubripes* Scherer, 1959: 244 (Nigeria-Cameroon: Mamfe), by original designation.

#### Distribution.

Democratic Republic of the Congo, Guinea**,** Nigeria and Rwanda (Fig. 319).

#### Ecology.

No information.

#### Notes.

Three species have been described.

### 

**[*Haltica* Illiger, 1801]**

= *Altica* Geoffroy, 1762

**[*Halticella* Jacoby, 1899b]**

= *Amphimela* Chapuis, 1875

**[*Halticopsis* Fairmaire, 1883a]**

Fig. 48

The genus *Halticopsis* (Fig. 48) is here transferred to the tribe Galerucini.

**References.** Fairmaire 1883a: 197, 1883b: 112; Biondi and D’Alessandro 2010a: 408.

**Type species.***Halticopsis spissicornis* Fairmaire, 1883b: 112 (Mountains of Abyssinia), designation by monotypy.

**Distribution.** Ethiopia.

**Ecology.** No information.

**Notes.** This Afrotropical genus, including a single species known from Ethiopia, is here transferred to the tribe Galerucini because of the absence of a metafemoral spring.

**[*Halticorthaea* Csiki, 1940 in Heikertinger and Csiki 1940]**

= *Amphimela* Chapuis, 1875

### Halticotropis

Fairmaire, 1886

http://species-id.net/wiki/Halticotropis

Figs 49 189–191 320

#### References.

Fairmaire 1886: 95; Bechyné 1964: 158; Biondi and D’Alessandro 2010a: 408.

#### Type species.

*Halticotropis multiplicata* Fairmaire, 1886: 95 (Madagascar), designation by monotypy.

#### Distribution.

Madagascar (Fig. 320).

#### Ecology.

No information.

#### Notes.

Two species have been described.

### 

**[*Halticova* Fairmaire, 1898]**

= *Amphimela* Chapuis, 1875

**[*Haltitarsus* Berthold, 1827]**

= *Dibolia* Latreille, 1829

### Hemipyxis

Chevrolat, 1836

http://species-id.net/wiki/Hemipyxis

Figs 50 192 –194 321

=Sebaethe Baly, 1864 (synonymized by Monrós and Bechyné 1956)
Asphaera Chevrolat, 1843 (pars)

#### References.

Chevrolat 1836: 387; 1843: 227; Baly 1864: 438; Monrós and Bechyné 1956: 1134; Bechyné 1958b: 193; 1960b: 110; Biondi and D’Alessandro 2010a: 408.

#### Type species.

*Hemipyxis*: *Haltica troglodytes* Olivier, 1808: 700 (India), by subsequent designation by Chevrolat (1845: 6); *Sebaethe*: *Haltica badia* Erichson, 1834: 274 (Philippines), by original designation.

#### Distribution.

Afrotropical (excluding Madagascar), Australian, Eastern Palaearctic, and Oriental regions (Fig. 321).

#### Ecology.

Polyphagous. This genus has been associated with herbaceous plants and shrubs belonging to many plant families (cf. Jolivet and Hawkeswood 1995).

#### Notes.

About thirty species are known from Sub-Saharan Africa. Six species of *Hemipyxis*, known from Madagascar, are here transferred to the genus *Pseudadorium* Fairmaire (see Notes in *Pseudadorium*).

### 

**[*Hermaeophaga* Foudras, 1859]**

Not present in the Afrotropical region.

**References.** Foudras, 1859: 147.

**Notes.** Species originally described in this Palaearctic genus were subsequently transferred to *Orthocrepis* Weise by Scherer (1961: 267).

### Hespera

Weise, 1889

http://species-id.net/wiki/Hespera

Figs 51 195 322

=Allomorpha Jacoby, 1892b (synonymized by Maulik 1926)

#### References.

Weise 1889: 638; Jacoby 1892b: 934; Maulik 1926: 137; Biondi and D’Alessandro 2010a: 408.

#### Type species.

*Hespera*: *Hespera sericea* Weise, 1889: 639 (China), by original designation; *Allomorpha*: *Allomorpha sericea* Jacoby, 1892b: 934 [Burma (=Myanmar): Carin Chebà], designation by monotypy.

#### Distribution.

Afrotropical (excluding Madagascar), Eastern Palaeartic, and Oriental regions (Fig. 322).

#### Ecology.

The Afrotropical species of this genus are mainly associated with plants in the families Anacardiaceae and Ericaceae (cf. Jolivet and Hawkeswood 1995; personal data).

#### Notes.

About thirty species are known from Sub-Saharan Africa.

### Hildebrandtina

Weise, 1910b

http://species-id.net/wiki/Hildebrandtina

Figs 52 196–197 323

#### References.

Weise 1910b: 464; Laboissière 1932: 584; Bechyné 1948b: 99; 1964: 131; Biondi and D’Alessandro 2010a: 408.

#### Type species.

*Hildebrandtina variegata* Weise, 1910b: 465 (Madagascar), by original designation.

#### Distribution.

Madagascar (Fig. 323).

#### Ecology.

No information.

#### Notes.

This genus, with about ten species known to occur in Madagascar, was transferred from the subfamily Galerucinae to the Alticinae (currently the tribe Alticini) by Biondi and D’Alessandro (2010a). The reason being the presence of a metafemoral spring, very similar to that described for the Oriental genus *Mandarella* Duvivier (1892b: 433) (cf. Furth and Suzuki 1994).

### Homichloda

Weise, 1902a

http://species-id.net/wiki/Homichloda

Figs 53 198 324

=Weiseana Jacoby, 1903a (synonymized by Cox 1997)

#### References.

Weise 1902a: 165; Jacoby 1903a: 16, 1906: 23 (as *Weiseana*); Cox 1997: 939.

#### Type species.

*Homichloda*: *Homichloda pauli* Weise, 1902a: 166 (Kwai), designation by monotypy; *Weiseana*: *Weiseana barkeri* Jacoby, 1903: 16 (Natal, Malvern), by original designation.

#### Distribution.

Kenya, Republic of South Africa (KwaZulu-Natal), Tanzania and Zambia (Fig. 324).

#### Ecology.

A genus that has been associated with a variety of *Acacia* species, trees in the family Fabaceae (Cox 1997).

#### Notes.

Three species have been described.

### Hyphasis

Harold, 1877b

http://species-id.net/wiki/Hyphasis

Figs 54 199 –200 325

= Hyphasoma Jacoby, 1903b (synonymized by Chen 1936)
Physoma Clark, 1863 (pars)

#### References.

Clark 1863: 165; Harold 1877b: 434; Jacoby 1901: 298; 1903b: 110; Maulik 1926: 158, 166; Chen 1936: 627; Heikertinger and Csiki 1940: 457; Bechyné 1948a: 10; 1958a: 90 (as *Hyphasoma*); Konstantinov and Vanderberg 1996: 369.

#### Type species

**.** *Hyphasis*: *Oedionychis magica* Harold, 1877b: 434 (India), by original designation. *Hyphasoma*: *Hyphasoma inconspicua* Jacoby, 1903b: 111 (India), by subsequent designation of Maulik (1926: 156).

#### Distribution.

Mascarene Islands (probably introduced), Oriental region and South-Eastern part of the Palaearctic region (Fig. 325).

#### Ecology.

This genus is associated with plants in the families Verbenaceae and Lamiaceae (cf. Jolivet and Hawkeswood 1995).

#### Notes.

The Malagasy species initially attributed to this Oriental genus were previously transferred to *Hyphasoma* Jacoby (Heikertinger and Csiki 1940) and then to *Physoma* Clark (Bechyné 1948a). Bechyné (1958a) reports *Hyphasis* (as *Hyphasoma*) *sita* (Maulik 1926: 158), described from Sri Lanka (= Ceylon), as an introduced species on the Mascarene Islands (Mauritius). We here confirm that this flea beetle genus does occur on the island of Mauritius.

### Jacobyana

Maulik, 1926

http://species-id.net/wiki/Jacobyana

Figs 55 201–202 326

#### References.

Maulik 1926: 284, 302–303; Biondi and D’Alessandro 2011: 49.

#### Type species.

*Sphaerophysa piceicollis* Jacoby, 1889c: 195 (Burma), by original designation.

#### Distribution.

Democratic Republic of the Congo, Malawi, Republic of South Africa (Eastern Cape Province), Zimbabwe (!) [Bulawayo, Shangani (BAQ)], and the Oriental region (Fig. 326).

#### Ecology.

No information for Afrotropical region.

#### Notes.

Three species have been described.

### 

**[*Jamesonia* Jacoby, 1895]**

= *Gabonia* Jacoby, 1893

### Kanonga

Bechyné, 1960b

http://species-id.net/wiki/Kanonga

Figs 56 203 327

#### References.

Bechyné 1960a: 54; Biondi and D’Alessandro 2003: 104; 2010a: 409.

#### Type species.

*Kanonga atra* Bechyné, 1960b: 54 (Upemba National Park: Kanonga), by original designation.

#### Distribution.

Democratic Republic of the Congo and Togo (Fig. 327).

#### Ecology.

No information.

#### Notes.

One species is known.

### Kenialtica

Bechyné, 1960b

http://species-id.net/wiki/Kenialtica

Figs 57 204 328

=Mediafra Scherer, 1961 (synonymy reported in Seeno and Wilcox 1982)

#### References.

Bechyné 1960b: 75; Scherer 1961: 266; Seeno and Wilcox 1982: 136; Biondi and D’Alessandro 2010a: 409.

#### Type species.

*Kenialtica***:** *Aphthona muhavura* Bechyné, 1955a: 207 (Rwanda, East of Muhavura), by original designation; *Mediafra*: *Aphthona muhavura* Bechyné, 1955a: 207 (Rwanda, East of Muhavura), by original designation.

#### Distribution.

Democratic Republic of the Congo, Kenya, Madagascar, Republic of South Africa (Limpopo), Republic of the Congo, Rwanda, Sierra Leoneand Uganda (!) [Budongo Forest, Sonso (BAQ)] (Fig. 328).

#### Ecology.

No information.

#### Notes.

Seven species have been described.

### Kimongona

Bechyné, 1959a

http://species-id.net/wiki/Kimongona

Figs 58, 59 205 329

=Mesocrepis Scherer, 1963 syn. n.

#### References.

Bechyné 1959a: 19; Scherer 1963: 668; Biondi and D’Alessandro 2010a: 409, 410.

#### Type species.

*Kimongona*: *Kimongona callifera* Bechyné, 1959a: 19 (Democratic Republic of the Congo: Mayumbe, Kimongo), by original designation. *Mesocrepis*: *Mesocrepis lindemannae* Scherer, 1963: 669 (Tanzania: Njombe), by original designation.

#### Distribution.

Democratic Republic of the Congo, Republic of South Africa (Mpumalanga), Rwanda, and Tanzania (Fig. 329).

#### Ecology.

No information.

#### Notes.

Three species are known.There are no significant diagnostic characters distinguishing *Mesocrepis* Scherer from *Kimongona*. Therefore, the following new synonymy is proposed: *Kimongona* Bechyné, 1959 = *Mesocrepis* Scherer, 1963 syn. n.Type material examined: *Mesocrepis lindemannae* Scherer, “Tanganjika, Uwemba b. Njombe, 2000 m, 8–11.XI.1958, leg. C. Lindemann”, paratypes 1♂ and 1♀ (NHMB).

### 

**[*Lactica* Erichson, 1847]**

Not present in the Afrotropical region.

**References.** Erichson 1847: 173; Weise 1902b: 302; Bryant 1940: 46; Bechyné 1959a: 19.

**Notes.** The Afrotropical species initially attributed to this Neotropical genus were transferred to *Phygasia* Chevrolat (Weise 1902b; Bryant 1940) and *Orthocrepis* Weise (Döberl 2012: 438); and the Malagasy species were transferred to *Antanemora* Bechyné (Bechyné 1959a).

### Lampedona

Weise, 1907a

http://species-id.net/wiki/Lampedona

Figs 60 206 330

#### References.

Weise 1907a: 399; Biondi and D’Alessandro 2010a: 409.

#### Type species.

*Lampedona tarsalis* Weise, 1907a: 399 (Spanish Guinea), designation by monotypy.

#### Distribution.

Democratic Republic of the Congo, Equatorial Guinea, Republic of the Congo and Tanzania (Fig. 330).

#### Ecology.

No information.

#### Notes. 

There are three described species.

### Lepialtica

Scherer, 1962a

http://species-id.net/wiki/Lepialtica

Figs 61 207 331

#### References.

Scherer 1962a: 30; Biondi and D’Alessandro 2010a: 409.

#### Type species.

*Lepialtica bicolor* Scherer, 1962a: 31 (Garamba National Park), designation by monotypy.

#### Distribution.

Democratic Republic of the Congo, Malawi (!) [Mulanje Mts (BAQ); Kasungu(BAQ)] and Zambia (!) [35 km S of Kasama (BAQ)] (Fig. 331).

#### Ecology.

No information.

#### Notes.

Four species are known (personal data).

### 

**[*Livolia* Jacoby, 1903]**

**References.** Jacoby, 1903a: 16; Furth and Suzuki 1994: 132.

**Notes.** Genus transferred to the subfamily Galerucinae (currently tribe Galerucini) by Furth and Suzuki (1994).

### Longitarsus

Berthold, 1827

http://species-id.net/wiki/Longitarsus

Figs 62 208 332)

#### References.

Berthold 1827: 401; Bechyné 1958c: 8; 1960b: 55; Biondi and D’Alessandro 2008b: 719; 2010a: 409.

#### Type species.

*Chrysomela atricilla* Linnaeus 1761: 166 (Europe), by subsequent designation by Maulik (1926: 333).

#### Distribution.

All zoogeographical regions (Fig. 332).

#### Ecology.

This genus is polyphagous and has been associated with several plant families, particularly the Boraginaceae, Asteraceae, Lamiaceae and Scrophulariaceae (cf. Jolivet and Hawkeswood 1995; personal data).

#### Notes.

Over one hundred species known from Madagascar and Sub-Saharan Africa (personal data).

### Luperomorpha

Weise, 1887

http://species-id.net/wiki/Luperomorpha

Figs 63 333

#### References.

Weise 1887: 202; 1915: 179; Bechyné 1959a: 1; Doguet 1979: 308; Biondi and D’Alessandro 2010a: 409; Döberl 2012: 439.

#### Type species.

*Luperomorpha trivialis* Weise, 1887: 204 (Siberia: Raddefka; Chingan), by original designation.

#### Distribution.

Cameroon, Democratic Republic of the Congo, Equatorial Guinea, Ethiopia (!) [Oromia region (BAQ)],Nigeria, and Saudi Arabia, Socotra Island (Yemen) and the Australian, Eastern Palaearctic and Oriental regions (Fig. 333).

#### Ecology.

Polyphagous (cf. Jolivet and Hawkeswood 1995). There is no ecological information on this genus for the Afrotropical region. *Luperomorpha biondii* Döberl (2012: 439) was collected in Socotra on *Cephalocroton socotranus* (Euphorbiaceae).

#### Notes. 

Two species have been described for the Afrotropical region: *Luperomorpha vittula* (Weise, 1915) [described as *Jamesonia* Jacoby but then transferred to *Luperomorpha* by Bechyné (1959a)] and *Luperomorpha biondii* Döberl. Concerning the presence of this genus in the Afrotropical region, we refer to the comments reported for *Gabonia* Jacoby.

### Lypnea

Baly, 1876a

http://species-id.net/wiki/Lypnea

Figs 64 209–210 334)

=Escaleriella Weise, 1907 (synonymized by Scherer 1961)
=Poephila Weise, 1895 (name preoccupied by *Poephila*Gould 1842: 93 [pl.], Aves, Estrildidae)
=Poephilina Csiki in Heikertinger and Csiki, 1940 (new name for *Poephila* Weise, 1895; synonymized by Bechyné 1968)

#### References.

Baly 1876a: 446; Weise 1895: 342; 1907a: 396; Csiki in Heikertinger and Csiki 1940: 349; Bechyné 1960a: 15; 1968: 1717−1718; Scherer 1961: 267; Biondi and D’Alessandro 2010a: 409.

#### Type species.

*Lypnea*: *Lypnea flava* Baly, 1876a: 446 (New Guinea, Batchian), designation by monotypy; *Escaleriella*: *Escaleriella marginata* Weise, 1907: 398 [Spanish Guinea (= Equatorial Guinea)], by present designation; *Poephila*: *Poephila lacessita* Weise, 1895: 342 (Addah), designation by monotypy.

#### Distribution.

Afrotropical (including Madagascar), Australian, Eastern Palaearctic, and Oriental regions (Fig. 334).

#### Ecology.

*Lypnea flaveola* (Bryant, 1944) collected on *Oncoba echinata* Oliver (Flacourtiaceae) (Bryant 1944b, as *Poephila*).

#### Notes.

About ten species are known from Madagascar and Sub-Saharan Africa . Bechyné (1968: 1718) considered *Escaleriella* Weise and *Lypnea* Baly to be separate genera because of the difference in the shape of their elytral epipleura: expanded in *Lypnea*, but straight and narrow in *Escaleriella*.

### 

**[*Macroorthocrepis* Pic, 1921]**

= *Phygasia* Chevrolat, 1836

### Malvernia

Jacoby, 1899b

http://species-id.net/wiki/Malvernia

Figs 65 211 335

#### References.

Jacoby 1899b: 346; Biondi 1998a: 37; Biondi and D’Alessandro 2003: 104; 2010a: 410.

#### Type species.

*Malvernia varicornis* Jacoby 1899b: 347 (KwaZulu-Natal, Malvern), designation by monotypy.

#### Distribution.

Malawi and the Republic of South Africa (Eastern Cape Province, Free State, Gauteng, KwaZulu-Natal, Limpopo, Mpumalanga, and North-West Province) (Fig. 335).

#### Ecology.

*Malvernia varicornis* has been collected on the flowers of *Burchellia bubalina* (L. f.) Simms (Rubiaceae) (Biondi 1998a).

#### Notes.

Two known species.

### Manobia

Jacoby, 1885

http://species-id.net/wiki/Manobia

Figs 66 212 336

=Afroalytus Scherer, 1961 (synonymized by Biondi 2001a)

#### References.

Jacoby 1885: 73; Scherer 1961: 269; 1962a: 54; Biondi 2001a: 648; Biondi and D’Alessandro 2010a: 410.

#### Type species.

*Manobia*: *Manobia nigripennis* Jacoby, 1885: 73 (Sumatra), by subsequent designation by Maulik (1926: 285, 407); *Afroalytus*: *Afroalytus kivuensis* Scherer, 1961: 269, 286 (Kivu: T. Kalehe, 2850 m), by original designation.

#### Distribution.

Central, Eastern [Kenya (!), Taita Hills, Ngangao (BAQ)] and Western Africa; Australian, Eastern Palaearctic, and Oriental regions (Fig. 336).

#### Ecology.

This genus is probably polyphagous and has been reported mainly from the following plant families: Epacridaceae, Urticaceae, Cyatheaceae, Asteraceae and Arecaceae (cf. Jolivet and Hawkeswood 1995).

#### Notes.

About fifteen species described from Sub-Saharan Africa.

### 

**[*Mantura* Stephens, 1831]**

Not present in the Afrotropical region.

= *Balanomorpha* Chevrolat, 1836 (synonymy reported in Heikertinger and Csiki 1940)

**References.** Stephens 1831: 285, 322; Chevrolat 1836: 393; Weise 1907b: 222; Bechyné 1955a: 220.

**Notes.** *Mantura quadriplagiata* Jacoby (1895: 321) transferred to *Podagrica* Chevrolat by Bechyné (1955a); *Balanomorpha aethiopica* Chapuis (1879: 13) transferred to *Neumannia* Weise nom. preocc.(= *Podagricina* Csiki; = *Podagrica* Chevrolat) by Weise (1907).

**[*Mediafra* Scherer, 1961]**

= *Kenialtica* Bechyné, 1960a

**[*Mesocrepis* Scherer, 1963]**

= *Kimongona* Bechyné, 1959a

### Metroserrapha

Bechyné, 1958a

http://species-id.net/wiki/Metroserrapha

Figs 67 213 337

#### References.

Bechyné 1958a: 86; Doguet 1974: 120; Biondi and D’Alessandro 2010a: 410.

#### Type species.

*Metroserrapha prima* Bechyné, 1958a: 86 (Mauritius Island), by original designation.

#### Distribution.

Madagascar and the Mascarene Islands (Fig. 337).

#### Ecology.

The species in this genus are probably polyphagous, and have been associated mainly with plants in the Ericaceae, Asteraceae and Polygonaceae (cf. Jolivet and Hawkeswood 1995).

#### Notes.

Seven species are known from the Mascarene Islands and about ten, as yet undescribed, from Madagascar (personal data).

### 

**[*Monodaltica* Bechyné, 1955b]**

= *Trachytetra* Sharp, 1886

### Montiaphthona

Scherer, 1961

http://species-id.net/wiki/Montiaphthona

Figs 68 214–215 338

#### References.

Scherer 1961: 282; 1962a: 17; Biondi and D’Alessandro 2003: 98; 2010a: 410.

#### Type species.

*Montiaphthona monticola* Scherer, 1961: 285 (Kivu: Mont Muhi), by original designation.

#### Distribution.

Democratic Republic of the Congo, Kenya, Republic of South Africa (!) [Mpumalanga, Mount Sheba (BAQ)], Rwanda, Tanzania and Uganda (Fig. 338)

#### Ecology.

The species of this genus generally live at altitudes above 2,500 m in mixed bamboo forests.

#### Notes.

Six species have been described.

### 

**[*Musaka* Bechyné, 1958a]**

= *Sphaeroderma* Stephens, 1831

### Myrcina

Chapuis, 1875

http://species-id.net/wiki/Myrcina

Figs 69 216 339

=Myrcinella Jacoby, 1901 (synonymized by Bechyné 1964)
=Xenaltica Baly, 1875 (synonymized by Laboissière 1942)

#### References.

Chapuis 1875: 124, 126; Baly 1875: 25; Jacoby 1901: 301; Laboissière 1942: 50; Bechyné 1964: 150; Biondi and D’Alessandro 2010a: 410.

#### Type species.

*Myrcina*: *Myrcina nigra* Chapuis, 1875: 127 (Vieux-Calabar), by original designation; *Myrcinella*: *Myrcina spectabilis* Baly, 1878b: 232 (Madagascar), by subsequent designation by Bechyné (1964: 150); *Xenaltica*: *Xenaltica murrayi* Baly, 1875: 26 (Old Calabar) (= *Myrcina nigra* Chapuis, 1875: 127), by present designation.

#### Distribution.

Madagascar and Sub-Saharan Africa (absent in the southern part of SAF) (Fig. 339).

#### Ecology.

Some species of this genus were collected from *Spathodea* sp. (Bignoniaceae) in Eastern Africa (cf. Jolivet and Hawkeswood 1995).

#### Notes.

Twenty-three species are known.

### 

**[*Myrcinella* Jacoby, 1901]**

= *Myrcina* Chapuis, 1875

### Neodera

Duvivier, 1891

http://species-id.net/wiki/Neodera

Figs 70 340

#### References.

Duvivier 1891: 316; Bechyné 1947b: 139; 1964: 152; Biondi and D’Alessandro 2010a: 410.

#### Type species.

*Crepidodera picticornis* Harold, 1877a: 107 (Madagascar), by present designation.

#### Distribution.

Madagascar. Weise (1923: 122) described *Neodera australis* from specimens from Australia (Queensland); however the generic placement of this species is not correct (C. Reid, 2010, pers. comm.) (Fig. 340).

#### Ecology.

No information.

#### Notes.

About fifteen known species.The subgenus*Neoderina* Bechyné (1952) is here considered to be a synonym of *Diphaulacosoma* Jacoby (see above).

### 

**[*Neoderina* Bechyné, 1952]**

= *Diphaulacosoma* Jacoby, 1892a

**[*Neumannia* Weise, 1907b]**

= *Podagrica* Chevrolat, 1836

**[*Niphraea* Baly, 1878a]**

= *Eriotica* Harold, 1877a

### Nisotra

Baly, 1864

http://species-id.net/wiki/Nisotra

Figs 71 217 –218 341

=Pseudonisotra Bechyné, 1968 (synonymized by Biondi and D’Alessandro 2010a)

#### References.

Baly 1864: 437; Bechyné 1955a: 220; 1959d: 153; 1960b: 88; 1968: 1719; Biondi and D’Alessandro 2010a: 411.

#### Type species.

*Nisotra*: *Haltica gemella* Erichson, 1834: 275 (Philippines: Luzon), by subsequent designation by Chapuis (1875: 42); *Pseudonisotra*: *Crepidodera tosta* Gerstaecker, 1871: 85 (Kenya: Mombasa), by original designation.

#### Distribution.

Afrotropical (including Madagascar), Australian, Eastern Palaearctic and Oriental regions (Fig. 341).

#### Ecology.

Species in this genus are mainly associated with plants in the family Malvaceae (cf. Jolivet and Hawkeswood 1995).

#### Notes.

About seventy species of this genus are known to occur in Madagascar and Sub-Saharan Africa.

### Notomela

Jacoby, 1899b

http://species-id.net/wiki/Notomela

Figs 72 219–220 342

#### References.

Jacoby 1899b: 357; Bryant 1931: 255; Scherer 1969: 371; Biondi and D’Alessandro 2010a: 411.

#### Type species.

*Notomela cyanipennis* Jacoby, 1899b: 357 (Cameroon), designation by monotypy.

#### Distribution.

Cameroon, Democratic Republic of the Congo, Equatorial Guinea (Fernando Poo Island), Ivory Coast, Liberia, Nigeria, Republic of South Africa (North-West Province and KwaZulu-Natal), Rwanda and Uganda, (Fig. 342).

#### Ecology.

*Notomela fulvicollis* Bryant, 1931 was collected on *Xanthoxylum capense* (Thunb.) Harv. (Rutaceae) in South East Africa (Bryant 1931).

#### Notes.

Four species have been described.

### Ntaolaltica

Biondi and D’Alessandro, in press

http://species-id.net/wiki/Ntaolaltica

Figs 73 221–223 343

#### References.

Biondi and D’Alessandro, in press.

#### Type species.

*Ntaolaltica antennata* Biondi and D’Alessandro, in press (Madagascar: Analamerana, 50 km SE of Diégo-Suarez), by original designation.

#### Distribution.

Madagascar (Fig. 343).

#### Ecology.

No information.

#### Notes.

A single species has been described.

### Nzerekorena

Bechyné, 1955b

http://species-id.net/wiki/Nzerekorena

Figs 74 224 344

#### References.

Bechyné 1955b: 507; Scherer 1959: 190; Scherer and Boppré 1997: 10, 32; Biondi and D’Alessandro 2003: 104; 2010a: 411.

#### Type species.

*Nzerekorena cerambycina* Bechyné, 1955b: 507 (French Guinea: Nzérékoré; Liberia: Kaouyéké; Dahomey: Forèt de Ketou), by original designation.

#### Distribution.

Benin, Cameroon, Democratic Republic of the Congo, Guinea, Kenya, Liberia, Malawi (!) [Mulanje Mts. (BAQ)], Nigeria and Uganda (Fig. 344).

#### Ecology.

No information.

#### Notes.

Nine species are known.

### 

**[*Ochrosis* Foudras, 1860]**

Not present in the Afrotropical region.

**References.** Foudras 1860: 147; Jacoby 1906: 17; Biondi and D’Alessandro 2010a: 411.

**Notes.** The species *Ochrosis natalensis* Jacoby (1906) was first attributed to this Palaearctic genus. However, examination of the type material resulted in the transfer of this species to the genus *Bechuana* Scherer (Biondi and D’Alessandro 2010a).

**[*Oedionychis* Latreille, 1829]**

Not present in the Afrotropical region.

**References.** Latreille 1829: 154; Konstantinov and Vandenberg 1996: 367.

**Notes.** Currently, four species that occur in southwestern Europe and North Africa are attributed to this genus (Konstantinov and Vanderberg 1996). Afrotropical species described as *Oedyonichis* were previously transferred to the genera *Eutornus* Clark, *Philopona* Weise, *Physodactyla* Chapuis, *Physoma* Clark, and *Physomandroya* Bechyné.

**[*Orneates* Jacoby, 1899b]**

= *Gabonia* Jacoby, 1893

### Orthocrepis

Weise, 1888

http://species-id.net/wiki/Orthocrepis

Figs 75 225–226 345

Crepidodera Chevrolat, 1836 (pars)
Hermaeophaga Foudras, 1860 (pars)
Lactica Erichson, 1847 (pars)

#### References.

Weise 1888: 850; Bechyné 1948a: 4 (as *Hermaeophaga*); 1954b: 677; 1955a: 224; 1964: 141; Scherer 1961: 267; 1963: 664; Biondi and D’Alessandro 2010a: 411.

#### Type species.

*Haltica ruficollis* Lucas, 1849: 546 (Algeria), designation by monotypy.

#### Distribution.

Afrotropical (including Madagascar), Oriental and Palaearctic regions (Fig. 345).

#### Ecology.

The species in this genus are mainly associated with plants in the family Euphorbiaceae, but also with Leguminosae and Malvaceae (cf. Jolivet and Hawkeswood 1995; Pollard 1957).

#### Notes.

About twenty-five species have been recorded in Sub-Saharan Africa and sixteen from Madagascar.

### Paradibolia

Baly, 1875

http://species-id.net/wiki/Paradibolia

Figs 76 227 346

#### References.

Baly 1875: 31; Weise 1912: 157; Bryant 1927: 617; Biondi and D’Alessandro 2010a: 411.

#### Type species.

*Paradibolia indica* Baly, 1875: 31 (India), designation by monotypy.

#### Distribution.

Cameroon, Democratic Republic of the Congo, Guinea, Namibia (!) [Fish River Canyon (ZMHB); Hereroland (ZMHB); Windhoek (BAQ); Waterberg National Park (BAQ)], Republic of South Africa, Sierra Leone, Australian and Oriental regions (Fig. 346).

#### Ecology.

Species in this genus are associated with plants from the family Lamiaceae in South Africa (personal data).

#### Notes.

Three species are known.

### 

= *Sesquiphaera* Bechyné, 1958a

### Perichilona

Weise, 1919

http://species-id.net/wiki/Perichilona

Figs 77 228 347

#### References.

Weise 1919: 202; Biondi and D’Alessandro 2010a: 411.

#### Type species.

*Perichilona rufa* Weise, 1919: 203 (Gaviro, Kwiro), by present designation.

#### Distribution.

Tanzania (Fig. 347).

#### Ecology.

No information.

#### Notes.

Two species have been described.

### Philopona

Weise, 1903

http://species-id.net/wiki/Philopona

Figs 78 229 348

Oedionychus Berthold, 1827 (pars)

#### References.

Weise 1903: 216; Laboissière 1942: 105; Biondi and D’Alessandro 2010a: 411.

#### Type species.

*Oedionychis* (?) *vernicata* Gerstaecker, 1871: 84 (Zanzibar), by original designation.

#### Distribution.

Afrotropical (excluding Madagascar), Australian, Oriental and Southern-Eastern Palaearctic regions (Fig. 348).

#### Ecology.

*Philopona usambarica* Csiki (in Heikertinger and Csiki 1940: 453) collected on *Thunbergia alata* Bojer ex Sims (Acanthaceae) in Kenya (Furth 1985).

#### Notes.

About twenty species known from Sub-Saharan Africa.

### Phygasia

Chevrolat, 1836

http://species-id.net/wiki/Phygasia

Figs 79 230 349

=Macroorthocrepis Pic, 1921 (synonymized by Bechyné 1960b)
Lactica Erichson, 1847 (pars)

#### References.

Chevrolat 1836: 387; Harold 1877b: 365; Fairmaire 1888: 156; Pic 1921: 14; Bryant 1940: 46; Bechyné 1960b: 82; Biondi and D’Alessandro 2010a: 412; in press.

#### Type species.

*Phygasia*: *Altica unicolor* Olivier, 1808: 699 (India), by original designation; *Macroorthocrepis*: *Macroorthocrepis pallidicolor* Pic, 1921: 14 (Abyssinia), designation by monotypy.

#### Distribution.

Afrotropical (excluding Madagascar), Oriental and Palaearctic regions (Fig. 349).

#### Ecology.

Species in this genus are mainly associated with plants in the family Asclepiadaceae (cf. Jolivet and Hawkeswood 1995).

#### Notes.

About thirty-five species have been described in Sub-Saharan Africa. The Madagascan species previously attributed to this genus have been transferred to the genus *Pseudophygasia* Biondi and D’Alessandro, in press.

### Phyllotreta

Chevrolat, 1836

http://species-id.net/wiki/Phyllotreta

Figs 80 231 350

#### References.

Chevrolat 1836: 391; Bryant 1942a: 145; Heikertinger 1943: 33; Bechyné 1955c: 61; Biondi and D’Alessandro 2010a: 412.

#### Type species.

*Chrysomela brassicae* Fabricius, 1787: 78 (Europe), by subsequent designation by Chevrolat (1845: 6).

#### Distribution.

All zoogeographical regions (Fig. 350).

#### Ecology.

A genus that has mainly been associated with plants in the families Cruciferae (= Brassicaceae), Resedaceae, and Capparidaceae (cf. Jolivet and Hawkeswood 1995).

#### Notes.

About forty species are known from the Arabian Peninsula, Madagascar and Sub-Saharan Africa.

### Physodactyla

Chapuis, 1875

http://species-id.net/wiki/Physodactyla

Figs 81 232–233 351

Oedionychus Berthold, 1827 (pars)

#### References.

Chapuis 1875: 83, 88; Scherer 1962a: 72; Biondi and D’Alessandro 2010a: 412.

#### Type species.

*Physonychis africana* Chapuis, 1875: 89 (East Africa), by original designation.

#### Distribution.

Democratic Republic of the Congo, Kenya, Republic of South Africa (Mpumalanga and KwaZulu-Natal), Sudan and Tanzania (Fig. 351).

#### Ecology.

*Physodactyla rubiginosa* (Gerstaecker 1871: 84) was collected from *Thunbergia alata* Bojer ex Sims (Acanthaceae) in Kenya (Furth 1985); *Physodactyla africana* (Chapuis) from *Digera arvensis* Forssk. (Amaranthaceae) in Sudan (Pollard 1957, as *Physonychis*).

#### Notes.

Six described species.

### Physoma

Clark, 1863

http://species-id.net/wiki/Physoma

Figs 82 234–235 352

=Tropidophora Thomson, 1858: 217 (synonymized by Jacoby 1888)
Hyphasis Harold, 1877 (pars)
Hyphasoma Jacoby, 1903 (pars)
Oedionychus Berthold, 1827 (pars)

#### References.

Clark 1863: 165; Thomson 1858: 217; Chapuis 1875: 83, 87; Harold 1877b: 434; Weise 1895: 344; Jacoby 1888: 205; 1903b: 110; Bechyné 1959c: 318; Scherer 1962a: 73 (as *Physonychis*); Biondi and D’Alessandro 2010a: 412.

#### Type species.

*Physoma*:*Physoma tripartitum* (Thomson, 1858) (Gabon)(*= Physonychis rugicollis* Clark, 1860 *in litteris*), by subsequent designation by Chapuis (1875); *Tropidophora*: *Tropidophora tripartita* Thomson, 1858: 217 (Gabon), designation by monotypy.

#### Distribution.

Central and Western Africa, and Madagascar (Fig. 352)

#### Ecology.

No information.

#### Notes.

Two species known from Sub-Saharan Africa and about twenty from Madagascar.The genus-name*Tropidophora* Thomson is not available because it was ambiguously applied (ICZN, 1999: art. 12.2.5).

### Physomandroya

Bechyné, 1959

http://species-id.net/wiki/Physomandroya

Figs 83 236–238 353

Asphaera Chevrolat, 1843 (pars)
Oedionychus Berthold, 1827 (pars)

#### References.

Chevrolat 1843: 227; Bechyné 1959c: 318: Biondi and D’Alessandro 2010a: 412.

#### Type species.

*Physomandroya decorsei* Bechyné, 1959c: 319 (Madagascar: Ambowombé), by original designation.

#### Distribution.

Madagascar (Fig. 353).

#### Ecology.

No information.

#### Notes. 

Seven described species. Bechyné (1959c) also transferred *Asphaera melanarthra* Fairmaire, 1886: 94 (= *Asphaera madagascariensis* Jacoby, 1892a: 573) to this genus.

### Physonychis

Clark, 1860

http://species-id.net/wiki/Physonychis

Figs 84 239 354

#### References.

Clark 1860: 29; Duvivier 1891: 424; Bechyné 1959c: 321; Biondi and D’Alessandro 2010a: 412.

#### Type species.

*Physonychis smaragdina* Clark, 1860: 31 (Western Africa), by original designation.

#### Distribution.

Sub-Saharan Africa (absent in the southern-western part of SAF and Madagascar). *Physonychis varicornis* Duvivier, 1891 from Madagascar was transferred to the genus *Physoma* Clark by Bechyné (1959c) (Fig. 354).

#### Ecology.

No information.

#### Notes.

About thirty known species.

### 

**[*Plectroscelis* Chevrolat, 1836]**

= *Chaetocnema* Stephens, 1831

### Podagrica

Chevrolat, 1836

http://species-id.net/wiki/Podagrica

Figs 85, 86 355

=Neumannia Weise, 1907 (name preoccupied by *Neumania* Lebert, 1879: 357, Acari, Unionicolidae)
=Podagricina Csiki in Heikertinger and Csiki 1940 (new name for *Neumannia* Weise, 1907) syn. n.
=Podagrixena Bechyné, 1968 (synonymized by Biondi and D’Alessandro 2010a)

#### References.

Chevrolat 1836: 394; Csiki in Heikertinger and Csiki 1940: 364; Weise 1907b: 223; Bryant 1942b: 229; Bechyné 1960b: 84; 1968: 1719; Biondi and D’Alessandro 2010a: 412.

#### Type species.

*Podagrica*: *Altica fuscipes* Fabricius, 1775: 114 (Europe), by subsequent designation by Maulik, (1926: 273); *Neumannia*: *Balanomorpha aethiopica* Chapuis, 1879: 13 (Ethiopia), by present designation;* Podagrixena*: *Podagrica decolorata* Duvivier, 1892a: 60 (Democratic Republic of the Congo: Ibembo), by original designation.

#### Distribution.

Afrotropical [including Madagascar (!): Nossibé (ZMHB); Ranohira (ZMHB); Tamatave (ZMHB)], Oriental , and Palaearctic regions (Fig. 355).

#### Ecology.

The species in this genus are mainly associated with plants in the family Malvaceae (cf. Jolivet and Hawkeswood 1995: 128); some are known to cause damage to cotton crops, *Gossypium* sp. (Malvaceae).

#### Notes.

About fifty species are known from Sub-Saharan Africa, with one having been recorded from Madagascar. There are no significant diagnostic characters distinguishing *Podagricina* Csiki from *Podagrica*. Therefore, the following new synonymy is proposed: *Podagrica* Chevrolat, 1836 = *Podagricina* Csiki in Heikertinger and Csiki, 1940 syn. n. Type material examined: *Balanomorpha aethiopica* Chapuis, “Bogos, 1870, Keren, O. Beccari”, 4 syntypes (MCSN).

### 

**[*Podagricina* Csiki in Heikertinger and Csiki 1940]**

= *Podagrica* Chevrolat, 1836

**[*Podagrixena* Bechyné, 1968]**

= *Podagrica* Chevrolat, 1836

**[*Poephila* Weise, 1895]**

= *Lypnea* Baly, 1876a

**[*Poephilina* Csiki in Heikertinger and Csiki 1940]**

= *Lypnea* Baly, 1876a

### Polyclada

Chevrolat, 1836

http://species-id.net/wiki/Polyclada

Figs 87 240–241 356

=Cladocera Hope, 1840 (synonymy reported in Achard 1922)
=Cladotelia Kolbe, 1894 (synonymy reported in Achard 1922)

#### References.

Chevrolat 1836: 375; Hope 1840: 169; Baly 1861: 198; Kolbe 1894: 86; Achard 1922: 4; Medvedev 1996: 261; Biondi and D’Alessandro 2010a: 413.

#### Type species.

*Polyclada*: *Clythra pectinicornis* Olivier, 1789: 31 (Africa), designation by monotypy.

#### Distribution.

Sub-Saharan Africa (absent in the south-western part of SAF and Madagascar), Saudi Arabia, and Yemen (Fig. 356).

#### Ecology.

This genus is generally associated with plants in the family Anarcadiaceae (cf. Jolivet and Hawkeswood 1995; Chaboo et al. 2007).

#### Notes.

Sixteen species have been described.

### Pratima

Maulik, 1931

http://species-id.net/wiki/Pratima

Figs 88 242 357

#### References.

Maulik 1931: 253; Bechyné 1958a: 84; Biondi and D’Alessandro 2010a: 413.

#### Type species.

*Pratima variabilis* Maulik, 1931: 253 (Seychelles: Silhouette and Mahe), by original designation.

#### Distribution.

Indian Ocean (Seychelles and Mascarene Islands) (Fig. 357).

#### Ecology.

The species of this genus live in forests (300–700 m) (Maulik 1931).

#### Notes.

There are eight known species.

### 

**[*Prototrigona* Chevrolat, 1837]**

Nomen nudum.

**Notes.** Chevrolat (1837: 411) uses this genus-name, without a description, for the following two unpublished species: “Glauca, *Dej*.” from Madagascar, and “Viridana, *Dej*.” from an unknown locality. “Glauca, *Dej*.” was also synonymized with “Prasinipennis, *Chevr*.”; this was also never published.

### Pseudadorium

Fairmaire, 1885

http://species-id.net/wiki/Pseudadorium

Figs 89 243–245 358

Asphaera Chevrolat, 1843 (pars)
Hemipyxis Chevrolat, 1836 (pars)

#### References.

Fairmaire 1885: 239; Bechyné 1958b: 193.

#### Type species.

*Pseudadorium vernicatum* Fairmaire, 1885: 239 (Madagascar), designation by monotypy.

#### Distribution.

Only found onMadagascar (Fig. 358).

#### Ecology.

No information.

#### Notes.

About twenty described species. We define this genus in a broader sense than Bechyné (1958b) did. He separated *Pseudadorium* from *Hemipyxis* Chevrolat on the basis of the distinctly vertically orientated elytral epipleura alone; not visible laterally in dorsal view (horizontally orientated and visible in lateral view in *Hemipyxis*). However, the elytral epipleural configuration is variable. Other diagnostic characters are more useful for separating these two genera, such as: frontal carina not extended towards the clypeus (Fig. 244) in *Pseudadorium* [extended in *Hemipyxis* (Fig. 193)]; frons distinctly raised distally (Fig. 244) in *Pseudadorium* [not raised in *Hemipyxis* (Fig. 193)]; elytra with distinct basal calli in *Pseudadorium* (absent in *Hemipyxis*); pronotum with anterior angles distinctly thickened and produced towards anterior (Fig. 243) in *Pseudadorium* [slightly thickened and not distinctly produced towards the anterior in *Hemipyxis* (Fig. 192)]; hind tibiae narrowly and less deeply channeled dorsally in *Pseudadorium* (Fig. 245) [broadly and more deeply channeled in *Hemipyxis* (Fig. 194)]. The following species from Madagascar, previously attributed to *Hemipyxis*, are here transferred to *Pseudadorium*: *Hemipyxis balyana* (Csiki in Heikertinger and Csiki 1940: 461) = *Pseudadorium balyanum* (Csiki in Heikertinger and Csiki 1940) comb. n.; *Hemipyxis brevicornis* (Jacoby, 1892a: 573) = *Pseudadorium brevicornis* (Jacoby, 1892a) comb. n.; *Hemipyxis cyanea* (Weise, 1910b: 439) = *Pseudadorium cyaneum* (Weise, 1910b) comb. n.; *Hemipyxis gynandromorpha* Bechyné, 1958c: 195 = *Pseudadorium gynandromorphum* (Bechyné, 1958c) comb. n.; *Hemipyxis latiuscula* Bechyné, 1958c: 195 = *Pseudadorium latiusculum* (Bechyné, 1958c) comb. n.; *Hemipyxis soror* (Weise, 1910b: 469) = *Pseudadorium soror* (Weise, 1910b) comb. n.

### 

**[*Pseudonisotra* Bechyné, 1968]**

= *Nisotra* Baly, 1864

### Pseudophygasia

Biondi & D’Alessandro, in press

http://species-id.net/wiki/Pseudophygasia

Figs 90 247 –248 359

Phygasia Chevrolat, 1836 (pars)

#### References.

Biondi and D’Alessandro, in press; Bechyné 1952: 249.

#### Type species.

*Crepidodera analis* Harold, 1877a: 107 (Madagascar), by original designation.

#### Distribution.

Only found on Madagascar (Fig. 359).

#### Ecology.

No information.

#### Notes.

Nine described species. All the species previously attributed to the genus *Phygasia* Chevrolat from Madagascar have been moved to *Pseudophygasia* (Biondi and D’Alessandro, in press).

### Psylliodes

Berthold, 1827

http://species-id.net/wiki/Psylliodes

Figs 91 248 360

#### References.

Berthold 1827: 401; Biondi 1996: 257; Nadein 2007: 317; Biondi and D’Alessandro 2010a: 413; Döberl 2012: 444.

#### Type species.

*Chrysomela chrysocephala* Linnaeus, 1758: 372 (Europe), by subsequent designation by Maulik (1926: 144).

#### Distribution.

Found inall the zoogeographical regions. In the Afrotropical region this genus is known from Ethiopia, Kenya, Republic of South Africa, Tanzania and Socotra Island (Yemen) (Fig. 360).

#### Ecology.

Known host plants for this genus fall in the plant families Cruciferae (= Brassicaeae), Solanaceae and Poaceae (also known as Gramineae) (cf. Jolivet and Hawkeswood 1995).

#### Notes.

There are seven described species.

### Pydaristes

Harold, 1875

http://species-id.net/wiki/Pydaristes

#### References.

Harold 1875: 446; Scherer 1961: 252; Biondi and D’Alessandro 2010a: 413.

#### Type species.

*Pydaristes attagenoides* Harold, 1875: 447 (Africa), designation by monotypy.

#### Distribution.

“Africa”.

#### Ecology.

No information.

#### Notes.

Only one species is known. Unfortunately, the location for the type material is unknown. The original description of *Pydaristes* appears to be identical or very close to that of *Amphimela* Chapuis. The synonymy proposed by Scherer (1961) between *Pydaristes* and *Blepharida* Chevrolat is unconvincing, because of the absence of dentiform emargination on the hind tibiae in *Pydaristes* (according to the original description), such emargination is characteristically present in *Blepharida*.

### Sanckia

Duvivier, 1891

http://species-id.net/wiki/Sanckia

Figs 92, 93 249–250 361

=Eugonotes Jacoby, 1897 syn. n.

#### References.

Duvivier 1891: 316; Jacoby 1897: 558; Bechyné 1956: 173; Medvedev 1995: 479; Biondi and D’Alessandro 2010a: 413.

#### Type species.

*Sanckia*: *Sanckia johanna* Duvivier, 1891: 316 (Madagascar: Antsianaka Forest), by original designation; *Eugonotes*: *Eugonotes longicornis* Jacoby, 1897: 559 (Madagascar: Diego-Suarez), designation by monotypy.

#### Distribution.

Burundi (!) [Bururi (ZSM)], Democratic Republic of the Congo, Ethiopia, Guinea, Kenya, Madagascar, Rwanda, Senegal, Uganda, and the Oriental region (Fig. 361).

#### Ecology.

No information.

#### Notes.

About twenty species are known from the Afrotropical region, most of these are from Madagascar. There are no significant diagnostic characters distinguishing *Eugonotes* Jacoby from *Sanckia*. The following new synonymy is therefore proposed: *Sanckia* Duvivier, 1891 = *Eugonotes* Jacoby, 1897 syn. n. Type material examined: *Eugonotes longicornis* Jacoby, “Madagascar, Diego Suarez”, syntype ♀ (BMNH).

### Serraphula

Jacoby, 1897

http://species-id.net/wiki/Serraphula

Figs 94 251–253 362

#### References.

Jacoby 1897: 556; Maulik 1929: 308; Biondi and D’Alessandro 2010a; 413; 2010b: 3.

#### Type species.

*Serraphula aenea* Jacoby, 1897: 557 (Mashonaland), designation by monotypy.

#### Distribution.

Republic of South Africa (Limpopo, Mpumalanga, Free State, KwaZulu-Natal, Eastern, and Western Cape Provinces) and Zimbabwe (Fig. 362).

#### Ecology.

Species in this genus are known to be associated with plants in the family Asteraceae (Biondi and D’Alessandro 2010b).

#### Notes.

Nineteen species have been described.

### Sesquiphaera

Bechyné, 1958a

http://species-id.net/wiki/Sesquiphaera

Figs 95, 96 254–256 363

=Paropsiderma Bechyné, 1958a syn. n.

#### References.

Bechyné 1958a: 92; Scherer 1961: 275; Biondi and D’Alessandro 2010a: 411, 413.

#### Type species.

*Sesquiphaera*: *Sphaeroderma mashonanum* Jacoby, 1900: 252 (Mashonaland: Salisbury), by original designation; *Paropsiderma*: *Sphaeroderma anthrax* Brancsik, 1910: 185 (Madagascar), designation by monotypy.

#### Distribution.

Democratic Republic of the Congo, Guinea, Guinea Bissau, Madagascar, Namibia, Republic of South Africa (Gauteng, Mpumalanga, and KwaZulu-Natal), Rwanda, Tanzania, and Zimbabwe (Fig. 363).

#### Ecology.

No information.

#### Notes.

There are about ten described species. No significant diagnostic characters distinguish *Paropsiderma* Bechyné from *Sesquiphaera*. The following new synonymy is therefore proposed: *Sesquiphaera* Bechyné, 1958 = *Paropsiderma* Bechyné, 1958 syn. n. Material examined: *Paropsiderma anthrax* (Brancsik) (det. J. Bechyné), “Madagascar, Joffreville, 13.V.1953, F. Kaiser”, 1 specimen (NHMB).

### Seychellaltica

Biondi, 2002b

http://species-id.net/wiki/Seychellaltica

Figs 97 257 –260 364

#### References.

Biondi 2002b: 358; Biondi and D’Alessandro 2010a: 413.

#### Type species.

*Chaetocnema mahensis* Maulik, 1931: 250 (Seychelles: Mahé), by original designation.

#### Distribution.

Indian Ocean (Seychelles) (Fig. 364).

#### Ecology.

The species in this genus are associated with indigenous forests in the Seychelles.

#### Notes.

Four species have been described.

### Sjostedtinia

Weise, 1910a

http://species-id.net/wiki/Sjostedtinia

Figs 98 261–262 365

#### References.

Weise 1910a: 205; Bryant 1953: 162; Scherer 1963: 648; Biondi and D’Alessandro 2010a: 413.

#### Type species.

*Sjostedtinia montivaga* Weise, 1910a: 206 (Kilimanjaro: Kibocho), by original designation.

#### Distribution.

Kenya, Tanzania, and Uganda, (Fig. 365).

#### Ecology.

This genus lives at high altitudes on Kilimanjaro and Mount Elgon. *Sjostedtinia montivaga* has been collected from *Lobelia deckeni* (Asch.) Hemsl. (Lobeliaceae) (Weise, 1910a), and *Sjostedtinia fordi* Bryant, 1953 from a *Senecio* sp. (Asteraceae) in Uganda (Bryant, 1953), and from a *Lobelia* sp. in Kenya (S. Zoia 2009, pers. comm.).

#### Notes.

Two species are known.

### Sphaeroderma

Stephens, 1831

http://species-id.net/wiki/Sphaeroderma

Figs 99 263–264 366

=Argosomus Wollaston, 1867 (synonymized by Scherer 1961)
=Musaka Bechyné, 1958a (synonymized by Scherer 1961)

#### References.

Stephens 1831: 328; Wollaston 1867: 152; Bryant 1943: 487; Bechyné 1958a: 89; 1968: 1702; Scherer 1961: 252; Biondi and D’Alessandro 2010a: 414.

#### Type species.

*Sphaeroderma*: *Altica testacea* Fabricius, 1775: 114 (Europe), by subsequent designation by Maulik, (1926: 316); *Argosomus*: *Argosomus epilachnoides* Wollaston, 1867: 152 (Cape Verde Islands: Brava), by subsequent designation by Konstantinov and Vanderberg (1996: 351); *Musaka*: *Sphaeroderma freyi* Bechyné, 1955b: 563 (Cameroon), by original designation.

#### Distribution.

Afrotropical (including Madagascar) and Australian, Oriental, and Palaearctic regions. The species of *Sphaeroderma* reported from the Neotropical and Nearctic regions should be attributed to different genera (cf. Savini and Furth 2001) (Fig. 366).

#### Ecology.

Species in this genus are mainly associated with plants in the families Asteraceae and Ranunculaceae (cf. Jolivet and Hawkeswood 1995).

#### Notes.

Over fifty species have been recorded from Sub-Saharan African and about ten from Madagascar.

### 

**[*Sphaerophysa* Baly, 1876b]**

= *Amphimela* Chapuis, 1875

### Stegnaspea

Baly, 1877

http://species-id.net/wiki/Stegnaspea

Figs 100 265 367

#### References.

Baly 1877: 181; Biondi and D’Alessandro 2010a: 414; D’Alessandro et al. 2012: 12.

#### Type species.

*Stegnaspea trimeni* Baly, 1877: 182 (Cape of Good Hope), designation by monotypy.

#### Distribution.

Republic of South Africa (Western Cape Province) and Tristan da Cunha (Fig. 367).

#### Ecology.

*Stegnaspea trimeni* collected from Poaceae (also known as Gramineae) in meadows (D’Alessandro et al. 2012).

#### Notes.

Six species are known.

### Stuckenbergiana

Scherer, 1963

http://species-id.net/wiki/Stuckenbergiana

Figs 101 368

#### References.

Scherer 1963: 670; Biondi and D’Alessandro 2010a: 414.

#### Type species.

*Podagrica glabrata* Jacoby, 1899b: 349 (KwaZulu-Natal: Umtenweni River; Eastern Cape Province: Port St. John), by original designation.

#### Distribution.

Republic of South Africa [Mpumalanga, KwaZulu-Natal, and Eastern Cape Province] (Fig. 368).

#### Ecology.

No information.

#### Notes.

Only one species is known.

### Terpnochlorus

Fairmaire, 1904

http://species-id.net/wiki/Terpnochlorus

Figs 102 266–267 369

Chaloenus Westwood, 1862 (pars)

#### References.

Fairmaire 1904: 269; Laboissière 1932: 575; Bryant 1927: 615 (as *Chaloenus*); Bechyné 1955b: 543; 1960b: 101; Furth and Suzuki 1994: 130; Biondi 2002b: 364; Biondi and D’Alessandro 2010a: 414.

#### Type species.

*Terpnochlorus perrieri* Fairmaire, 1904: 269 (Madagascar: Soalala), designation by monotypy.

#### Distribution.

Botswana, Democratic Republic of the Congo, Gambia, Guinea Bissau, Madagascar, Mali, Namibia, Sierra Leone, and South America (Venezuela and Mexico) (Fig. 369).

#### Ecology.

This genus lives in moist habitats generally associated with plans from the family Juncaceae (Biondi 2002b).

#### Notes.

Two species have been described from the Afrotropical region. Bechynè (1955b) synonymised *Chaloenus viridis* Bryant (Bryant 1927) with *Terpnochlorus perrieri* Fairmaire [*Terpnochlorus perrieri* Fairmaire = *Chaloenus viridis* Bryant (Bryant 1927)].

### 

**[*Torodera* Weise, 19082a]**

= *Argopistoides* Jacoby, 1892b

### Toxaria

Weise, 1903

http://species-id.net/wiki/Toxaria

Figs 103 370

#### References.

Weise 1903: 215; 1912: 157; Laboissière 1941: 318; Bryant 1943: 488; Biondi and D’Alessandro 2010a: 414.

#### Type species.

*Galleruca indica* Fabricius, 1798: 98 (Western Cape), by original designation.

#### Distribution.

Democratic Republic of the Congo, Kenya, the Republic of South Africa, and Uganda (Fig. 370).

#### Ecology.

No information.

#### Notes.

Five species are known.

### Trachytetra

Sharp, 1886

http://species-id.net/wiki/Trachytetra

Figs 104 268–269 371

=Monodaltica Bechyné, 1955b (synonymized by Konstantinov and Prathapan 2008)

#### References.

Sharp 1886: 448; Bechyné 1955b: 509; Scherer 1962a: 18 (as *Monodaltica*); Konstantinov and Prathapan 2008: 413; Biondi and D’Alessandro 2010a: 414.

#### Type species.

*Phyllotreta rugulosa* Broun, 1880: 636 (New Zealand), by original designation; *Monodaltica*: *Monodaltica guineensis* Bechyné, 1955b: 510 (French Guinea), by original designation.

#### Distribution.

Cameroon, Democratic Republic of the Congo, Ghana, Guinea, Nigeria, Sierra Leone, and Australian, Eastern Palaearctic, and Oriental regions (Fig. 371).

#### Ecology.

No information.

#### Notes.

Five species have been described from Sub-Saharan Africa.

### Tritonaphthona

Bechyné, 1960b

http://species-id.net/wiki/Tritonaphthona

Figs 105 270 372

#### References.

Bechyné 1960b: 70; Biondi and D’Alessandro 2010a: 416.

#### Type species.

*Aphthona longicornis* Laboissière, 1942: 21 (Albert National Park), by original designation; Biondi and D’Alessandro 2010a: 415.

#### Distribution.

Democratic Republic of the Congo (Fig. 372).

#### Ecology.

No information.

#### Notes.

One described species.

### 

**[*Thrymnes* Weise, 1895]**

= *Gabonia* Jacoby, 1893

**[*Tropidophora* Thomson, 1858]**

= *Physoma* Clark, 1863

### Upembaltica

Bechyné, 1960b

http://species-id.net/wiki/Upembaltica

Figs 106 271 –272 373

#### References.

Bechyné 1960b: 53; Biondi and D’Alessandro 2003: 104; 2010a: 416.

#### Type species.

*Upembaltica scolytina* Bechyné 1960b: 53−54 (Upemba National Park: Lupiala; Kaswabilenga), by original designation.

#### Distribution.

Democratic Republic of the Congo (Fig. 373).

#### Ecology.

No information.

#### Notes.

Only one species has been recorded.

### 

**[*Weiseana* Jacoby, 1906]**

= *Homichloda* Weise, 1902

### Xanthophysca

Fairmaire, 1901

http://species-id.net/wiki/Xanthophysca

Figs 107 273–275 374

#### References.

Fairmaire 1901: 242; Achard 1915: 31; Bechyné 1954a: 46; Biondi and D’Alessandro 2010a: 417.

#### Type species.

*Xanthophysca perrieri* Fairmaire, 1901: 242 (Madagascar: Saberbieville), designation by monotypy.

#### Distribution.

Madagascar (Fig. 374).

#### Ecology.

No information.

#### Notes.

Five species have been described. These usually display different colour varieties. Two species from Madagascar, previously included in the genus *Blepharida*, are here attributed to this genus: *Xanthophysca multiguttata* (Duvivier, 1891: 242) comb. n. and *Xanthophysca insignis* (Brancsik, 1897: 130) comb. n.

### 

**[*Xenaltica* Baly, 1875]**

= *Myrcina* Chapuis, 1875

### Yemenaltica

Scherer, 1985

http://species-id.net/wiki/Yemenaltica

Figs 108 276–277 375

#### References.

Scherer 1985: 86; Medvedev 1996: 261; Biondi and D’Alessandro 2010a: 417; Döberl 2012: 444.

#### Type species.

*Yemenaltica scorteccii* Scherer, 1985: 86 (Yemen: El Kasaba), by original designation.

#### Distribution.

Arabian Peninsula and Socotra Island (Yemen) (Fig. 375).

#### Ecology.

No information.

#### Notes.

Two species have been described.

### Zomba

Bryant, 1922b

http://species-id.net/wiki/Zomba

Figs 109 376

#### References.

Bryant 1922a: 263; Biondi and D’Alessandro 2010a: 417.

#### Type species.

*Zomba gossypii* Bryant, 1922a: 264 (Nyasaland: Luchenza; N.W. Rhodesia: Livingstone), designation by monotypy.

#### Distribution.

Malawi and Zimbabwe (Fig. 370).

#### Ecology.

The only species in this genus was collected from Cotton plants, *Gossypium* sp. (Malvaceae) (Bryant 1922a).

#### Notes.

Only one species is known. This genus is particularly interesting because it is the only representative of the tribe Monoplatini in the Afrotropical region. This tribe occurs almost exclusively in the Neotropical and southern part of the Nearctic regions. The only exceptions are the genera *Zomba* and *Opisthopygme* Blackburn (1896: 41); this last genus is occurring with two species in the Australian region.

## Discussion

There are 99 flea beetle genera known from the Afrotropical region. Of these, 83 occur in continental Sub-Saharan Africa, with the different regions each having a varying number of species: CAF: 63; EAF; 55; SAF: 58; and WAF: 44. Furthermore, 39 genera are known from Madagascar, 9 from the Mascarene Islands and 5 from the Seychelles Islands (Table 1, Fig. 377). However, these numbers are still provisional as information concerning the Afrotropical flea beetle fauna is limited, particularly for Madagascar.

Our preliminary analysis indicates that this fauna is distinct and can be separated from the faunas of other zoogeographical regions. In Fig. 381, a dendrogram obtained from the cluster analysis [Coincidence index and Weighted Pair Group Method using Arithmetic averaging (WPGMA) (cf. Biondi 2006; Biondi and D’Alessandro 2010a)] performed, using the presence/absence data of flea beetle genera in each of the OGUs (Operational Geographical Unit) considered, is reported. Results reveal a main Sub-Saharan cluster, including the four continental OGUs, these in turn form two subclusters, namely a central-western (WAF-CAF) subcluster and a southern-eastern (EAF-SAF) subcluster. Madagascar (MAD) also forms part of the Sub-Saharan cluster, completing the “Afrotropical group”.

The Seychelles (SEY) and the Mascarene islands (MAS) are more closely associated with the other zoogeographical regions [(NAR-NTR)(PAR-ORR)AUR)] because of the occurrence of a high percentage of widespread genera that characterize the flea beetle fauna of these two archipeligoes. Moreover, faunistic similarity based on the widespread flea beetle genera also clusters the other zoogeographical regions together in two distinct groups, namely the Palaearctic-Oriental-Australian regions [(PAR-ORR)AUR] and Nearctic-Neotropical regions (NAR-NTR).

The geographic distribution of Afrotropical flea beetle genera is therefore well characterized and it has distinct Malagasy and Sub-Saharan African components (Fig. 381).

The percentage of Alticini genera endemic to the Afrotropical region is very high (71.0%), with the following distribution: Sub-Saharan Africa, 52 genera; Madagascar, 12; Seychelles Islands, 1; Sub-Saharan Africa-Madagascar, 4; Madagascar-Mascarene Islands, 1; Seychelles-Mascarene Islands, 1 (Fig. 378). Within the endemic Sub-Saharan Africa component, only 6 genera occur in all four subregions, while 25 genera occur only in one subregion (SAF: 11; CAF: 9; EAF: 5). There are no exclusively endemic flea beetle genera in WAF (Fig. 378).

The percentage of genera occurring in both the Afrotropical and another zoogeographical region is 32.0%, with the cosmopolitan component significant and well represented [8.0% of the total of 99] (Fig. 379). The Afrotropical region shares the highest percentage of genera with the Oriental (27.0%) and Palaearctic (27.0%) regions (Fig. 380). The co-presence of genera in both the Afrotropical and Oriental regions, often called Palaeotropical distribution, may be due to a possible Gondwanian origin., *Sanckia* is such an example, although the genus occurs mainly in Madagascar, species are also found in Sub-Saharan Africa and the southern part of the Oriental region. Other examples are the genera *Argopistoides* and *Jacobyana* which occur in Sub-Saharan Africa and the Oriental region and are absent from Madagascar; and *Amphimela*, *Chabria*, *Nisotra*, and *Paradibolia*, which occur in the Afrotropical, Oriental and the Australian regions. Other genera, such as *Bikasha*, *Hemipyxis*, *Luperomorpha*, *Lypnea, Manobia*, *Philopona* and *Trachytetra* occur not only in the Afrotropical (including Madagascar, although infrequently), Oriental and, generally Australian regions, but also in the eastern part of the Palaearctic region. More specifically, the Palaeartic region shares 27 flea beetle genera with the Afrotropical region (Fig. 380), including the unique Pan-African flea beetle genus, *Angulaphthona*, which occurs in Mediterranean Africa, Sub-Saharan Africa and Madagascar (cf. Biondi and D’Alessandro 2006). A significantly lower percentage of genera (20.0%) occur in both the Afrotropical and Australian regions, although all of these can also be found throughout the Oriental region.

As expected, the lowest percentage of genera occur in both the Afrotropical/Nearctic regions (10.0%) and Afrotropical/Neotropical regions (10.0%). All genera common to the Afrotropical, Nearctic and Neotropical regions are also found in all other zoogeographical regions with the exception of the genus *Terpnochlorus*, which only occurs in the Afrotropical region, Venezuela and Mexico (cf. Furth and Suzuki 1994). Moreover, the possible synonymy between the genera *Abrarius* from Madagascar and *Gioia* from South America (see above), if confirmed, could indicate an interesting zoogeographical connection among the ancient regions of Gondwana (Biondi and D’Alessandro 2010a).

As reported in Biondi and D’Alessandro (2010a), other likely Gondwanian elements in the Afrotropical flea beetle fauna are:

The unique Afrotropical genus *Zomba* belonging to the tribe Monoplatini, which mainly occurs in the Neotropical region with a few species found in the Nearctic region. The genus *Opisthopygme*, also from the Monoplatini, is also present in Australia (see above).

Two new, as yet undescribed, flea beetle genera that occur in Madagascar and South Africa (Western Cape Province). Both these genera have clavate or subclavate antennae with 11 segments, are subsphaerical in shape and very small, characteristics they share with related genera in Central America, such as: *Bubiscus* Savini, Furth and Joly (2009: 53), a recently described Costa Rican genus (1 species); *Normaltica* Konstantinov (2002: 2), an endemic genus from Great Antilles (2 species); *Clavicornaltica* Scherer (1974: 58), a genus occurring in the Oriental (18 species) and Australian regions (1 species) (cf. Konstantinov and Duckett 2005). Other very closely related flea beetle genera, but with a reduced number of antennal segments, are: *Kiskeya* Konstantinov and Chamorro-Lacayo (2006: 276), which has nine-segmented clavate antennae - 2 species in the Dominican Republic; and *Monotalla* Bechyné (1956: 588), which has ten-segmented clavate antennae, with 1 species in Guadalupe (cf. Savini and Furth 2001: 907).

There are 39 flea beetle genera known from Madagascar, 13 of which are endemic. One of them, *Metroserrapha* Bechyné, also occurs in the Mascarene Islands (Biondi and D’Alessandro 2010a; 2012, in press). Some of these Madagascan genera, such as *Neodera*, *Physomandroya*, *Pseudadorium*, *Pseudophygasia*, and *Xanthophysca*, show clear African affinities, but *Antanemora*, *Ntaolaltica*, and *Metroserrapha* are more closely related to Oriental genera (Biondi and D’Alessandro 2012, in press). The remaining endemic Malagasy genera, *Anaxerta*, *Diphaulacosoma*, *Halticotropis*, and *Hildebrandtina*, are probably all very ancient. Establishing their affinities with certainty, whether African or Oriental, is very difficult using only a comparative morphological approach.

**Table 1.** Occurrence of Alticinae genera of the Chrysomelidae in different areas of the Afrotropical region and other zoogeographical areas (see text for abbreviations) (updated from Biondi and D’Alessandro 2010a).

Genus	WAF	CAF	EAF	SAF	MAD	SEY	MAS	PAR	NAR	NTR	ORR	AUR
*Abrarius*					X					?		
*Afroaltica*				X								
*Afrocrepis*				X	X							
*Afrorestia*		X	X	X	X							
*Alocypha*			X	X								
*Altica*	X	X	X	X	X		X	X	X	X	X	X
*Amphimela*	X	X	X	X	X			X			X	X
*Anaxerta*					X							
*Angulaphthona*	X	X	X	X	X			X				
*Antanemora*					X							
*Aphthona*	X	X	X	X	X		X	X	X		X	X
*Argopistes*	X	X	X	X	X			X	X	X	X	X
*Argopistoides*	X	X	X	X							X	
*Bangalaltica*		X										
*Bechuana*				X								
*Bechynella*	X	X										
*Bezdekaltica*			X									
*Bikasha*	X	X	X	X	X	X		X			X	
*Biodontocnema*				X								
*Blepharida*	X	X	X	X				X	X	X	X	X
*Carcharodis*		X		X	X							
*Celisaltica*		X										
*Chabria*	i?			i?	X						X	
*Chaetocnema*	X	X	X	X	X	X	X	X	X	X	X	X
*Chaillucola*		X										
*Chirodica*				X								
*Collartaltica*	X	X	X	X								
*Decaria*	X	X	X	X								
*Diamphidia*		X	X	X								
*Dibolia*	X	X	X	X				X	X	X		
*Dimonikaea*		X										
*Diphaulacosoma*					X							
*Djallonia*	X	X										
*Drakensbergianella*				X								
*Dunbrodya*			X	X								
*Epitrix*	X	X	X	X	X		X	X	X	X	X	X
*Eriotica*			X									
*Eurylegna*		X	X									
*Eutornus*	X	X	X	X	X							
*Gabonia*	X	X	X	X								
*Guilielmia*		X										
*Guinerestia*	X	X										
*Halticotropis*					X							
*Hemipyxis*	X	X	X	X				X			X	X
*Hespera*	X	X	X	X				X			X	
*Hildenbrandtina*					X							
*Homichloda*		X	X	X								
*Hyphasis*							i?	X			X	
*Jacobyana*		X	X	X							X	
*Kanonga*	X	X										
*Kenialtica*	X	X	X	X	X							
*Kimongona*		X	X	X								
*Lampedona*		X	X									
*Lepialtica*		X	X									
*Longitarsus*	X	X	X	X	X	X	X	X	X	X	X	X
*Luperomorpha*		X	X					X			X	X
*Lypnea*	X	X	X	X	X			X			X	X
*Malvernia*			X	X								
*Manobia*	X	X	X					X			X	X
*Metroserrapha*					X		X					
*Montiaphthona*		X	X	X								
*Myrcina*	X	X	X	X	X							
*Neodera*					X							
*Nisotra*	X	X	X	X	X			X			X	X
*Notomela*	X	X		X								
*Ntaolaltica*					X							
*Nzerekorena*	X	X	X									
*Orthocrepis*	X	X	X	X	X			X			X	
*Paradibolia*	X	X		X							X	X
*Perichilona*			X									
*Philopona*	X	X	X	X				X			X	X
*Phygasia*	X	X	X	X				X			X	
*Phyllotreta*	X	X	X	X	X			X	X	X	X	X
*Physodactyla*		X	X	X								
*Physoma*	X	X			X							
*Physomandroya*					X							
*Physonychis*	X	X	X	X								
*Podagrica*	X	X	X	X	X			X			X	
*Polyclada*	X	X	X	X				X				
*Pratima*						X	X					
*Pseudadorium*					X							
*Pseudophygasia*					X							
*Psylliodes*			X	X				X	X	X	X	X
*Sanckia*	X	X	X		X						X	
*Serraphula*				X								
*Sesquiphaera*	X	X	X	X	X							
*Seychellaltica*						X						
*Sjostedtinia*		X	X									
*Sphaeroderma*	X	X	X	X	X		X	X	X	X	X	X
*Stegnaspea*				X								
*Stuckenbergiana*				X								
*Terpnochlorus*	X	X		X	X					X		
*Toxaria*		X	X	X								
*Trachytetra*	X	X						X			X	X
*Tritonaphthona*		X										
*Upembaltica*		X										
*Xanthophysca*					X							
*Yemenaltica*			X					X				
*Zomba*			X	X								

## Figure plates

**Figures 2–10.**
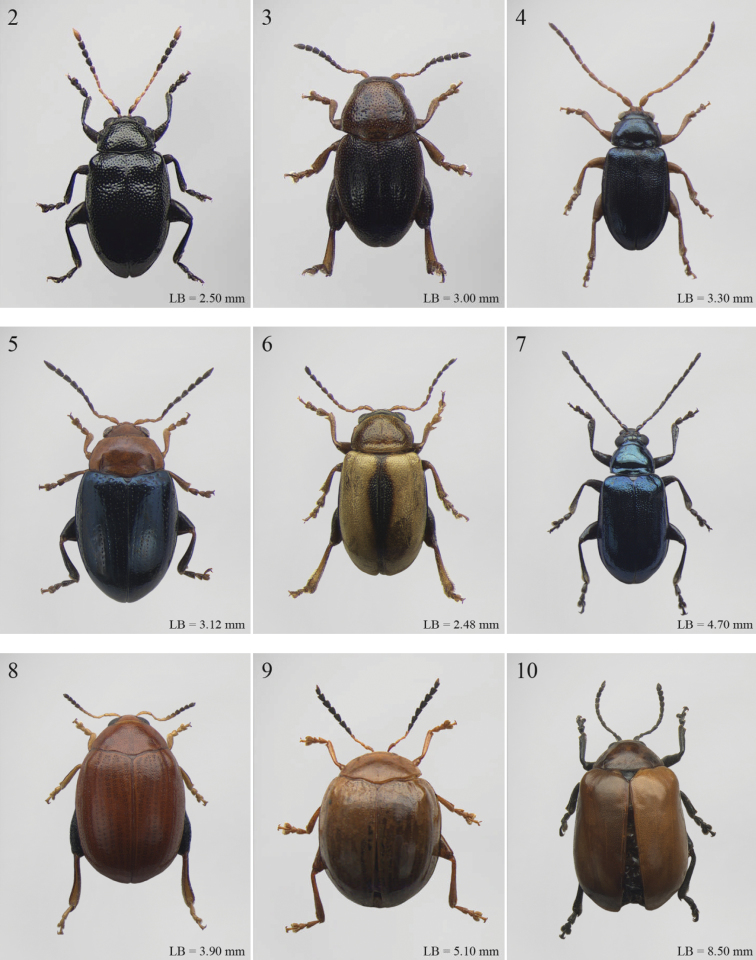
Habitus. **2** *Abrarius cribrosus* Fairmaire **3** *Afroaltica subaptera* Biondi & D’Alessandro **4 ***Afrocrepis malvernensis* (Jacoby) **5** *Afrorestia peringueyi* (Jacoby) **6** *Alocypha bimaculata* (Jacoby) **7** *Altica madagascariensis* (Allard) **8** *Amphimela bryanti* (Csiki) **9** *Amphimela* (ex *Sphaerophysa* Baly) *heikertingeri* (Bechyné) **10** *Anaxerta castanea* Fairmaire.

**Figures 11–19.**
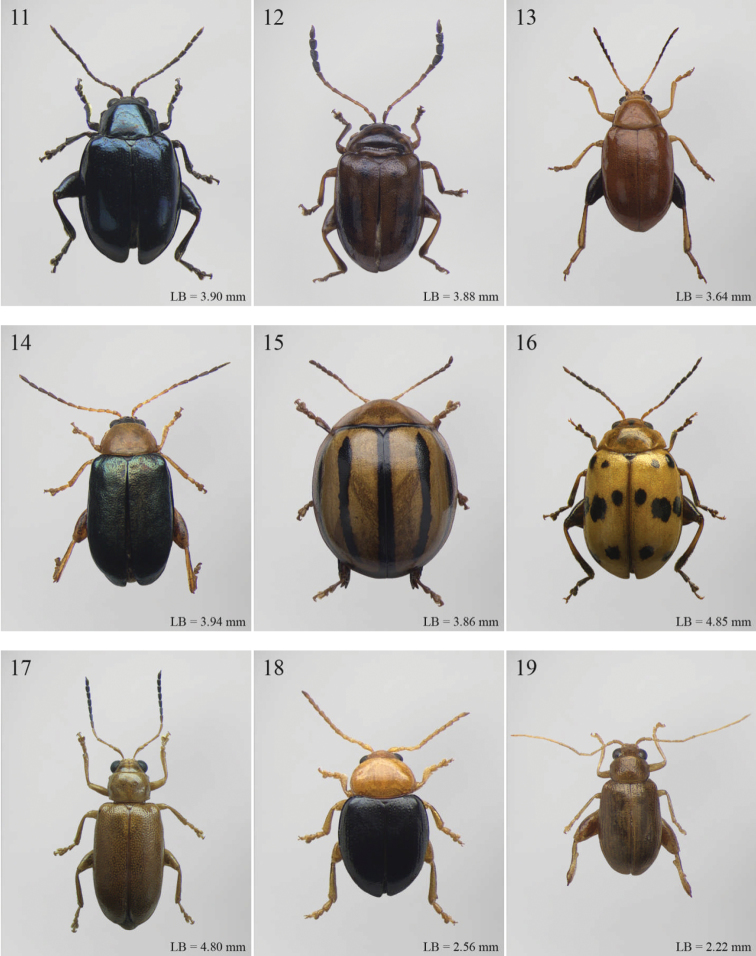
Habitus **.11** *Angulaphthona heteromorpha* (Bechyné) **12** *Antanemora ghesquierei* (Bechyné) **13** *Aphthona senegalensis* Jacoby **14** *Aphthona* (ex *Ethiopia* Scherer) *tricolor* (Scherer) **15** *Argopistes sexvittatus* Bryant **16** *Argopistoides africanus* (Bryant) **17** *Bangalaltica antennalis* Bechyné 18. *Bechuana nigripes* Scherer **19** *Bechynella pallens* (Bechyné).

**Figures 20–28.**
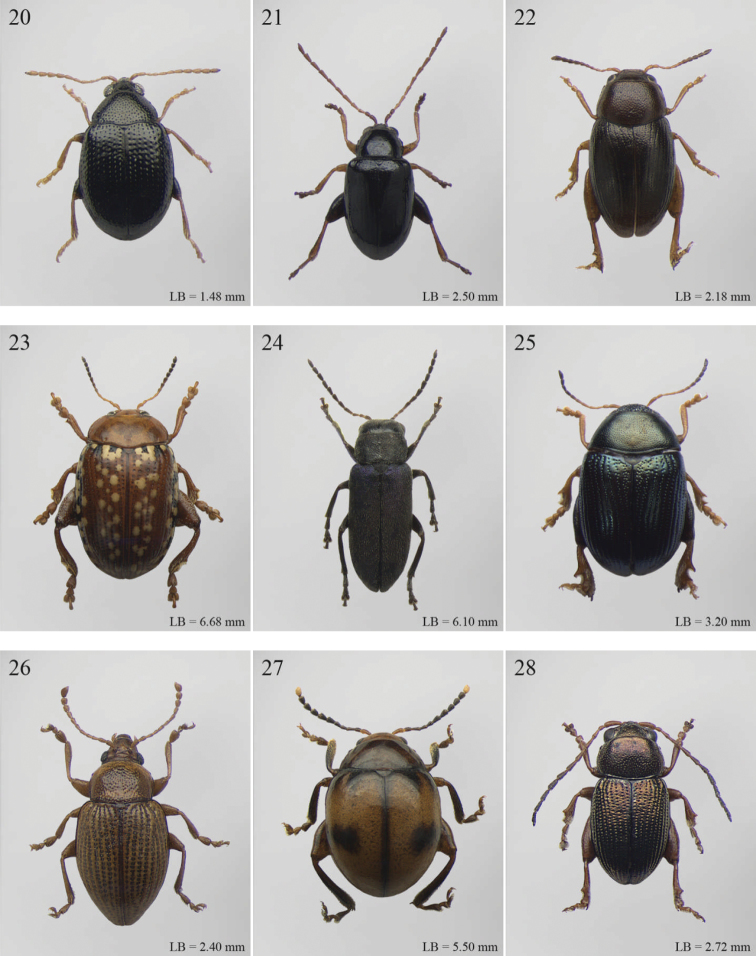
Habitus. **20** *Bezdekaltica socotrana* Döberl **21** *Bikasha tenuipunctata* Maulik **22** *Biodontocnema brunnea* Biondi **23** *Blepharida ornata* Baly **24** *Buphonella nigroviolacea* (Allard) (genus transferred to Galerucini) **25** *Carcharodis rugiceps* (Baly). **26** *Celisaltica ruwenzorica* Biondi **27** *Chabria bezanozana* Biondi & D’Alessandro **28** *Chaetocnema purpurea* Jacoby.

**Figures 29–37.**
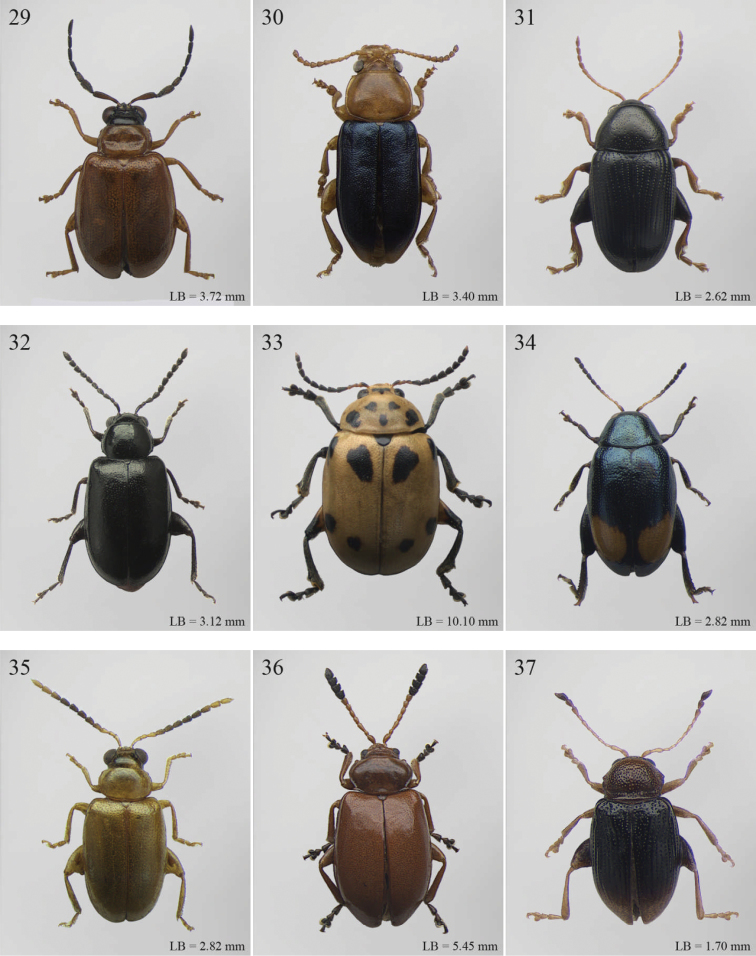
Habitus. **29** *Chaillucola formicicornis* Bechyné **30** *Chirodica chalcoptera* Germar **31 ***Collartaltica cryptostoma* Bechyné **32** *Decaria abdominalis* Jacoby **33** *Diamphidia nigroornata* Stål **34 ***Dibolia bimaculata* Jacoby **35** *Dimonikaea descarpentriesi* Bechyné **36** *Diphaulacosoma laevipenne* Jacoby **37** *Djallonia maindra* Bechyné.

**Figures 38–46.**
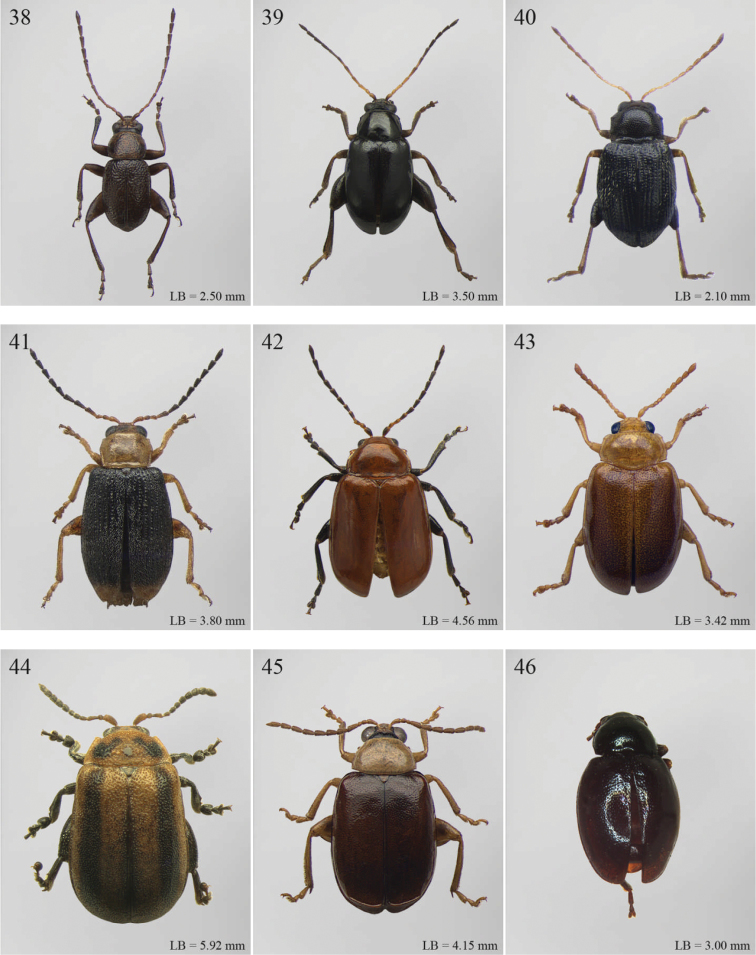
Habitus**. 38** *Drakensbergianella rudebecki* Biondi & D’Alessandro **39** *Dunbrodya nitida* Jacoby **40** *Epitrix aethiopica* Weise **41** *Eriotica fuscipennis* Harold **42** *Eurylegna fulva* Weise **43** *Eurylegna* (ex *Eurylegniella* Scherer) *guineensis* Bechyné **44** *Eutornus rugicollis* (Jacoby) **45** *Gabonia unicostata* Jacoby **46** *Guilielmia monticola* Weise.

**Figures 47–55.**
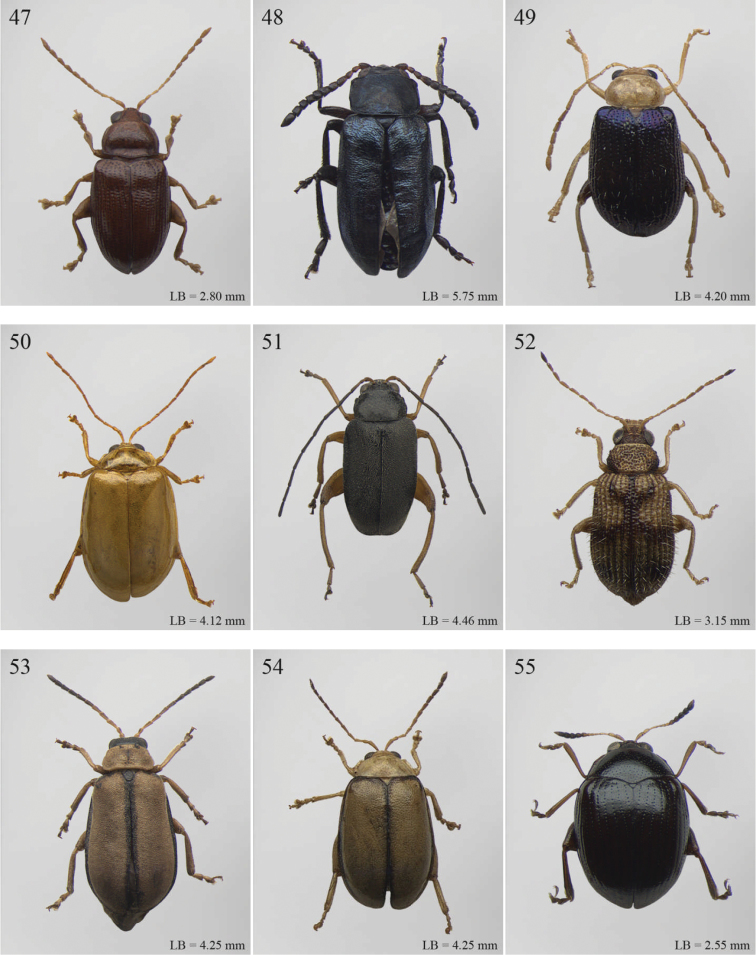
Habitus. **47** *Guinerestia rubra* Scherer **48** *Halticopsis spissicornis* Fairmaire (genus transferred to Galerucini) **49** *Halticotropis costipennis* Bechyné **50** *Hemipyxis africana* (Allard) **51** *Hespera africana* Jacoby **52** *Hildebrandtina similis* Bechyné **53** *Homichloda barkeri* (Jacoby) **54** *Hyphasis sita* (Maulik) **55** *Jacobyana bezdeki* Biondi & D’Alessandro.

**Figures 56–64:**
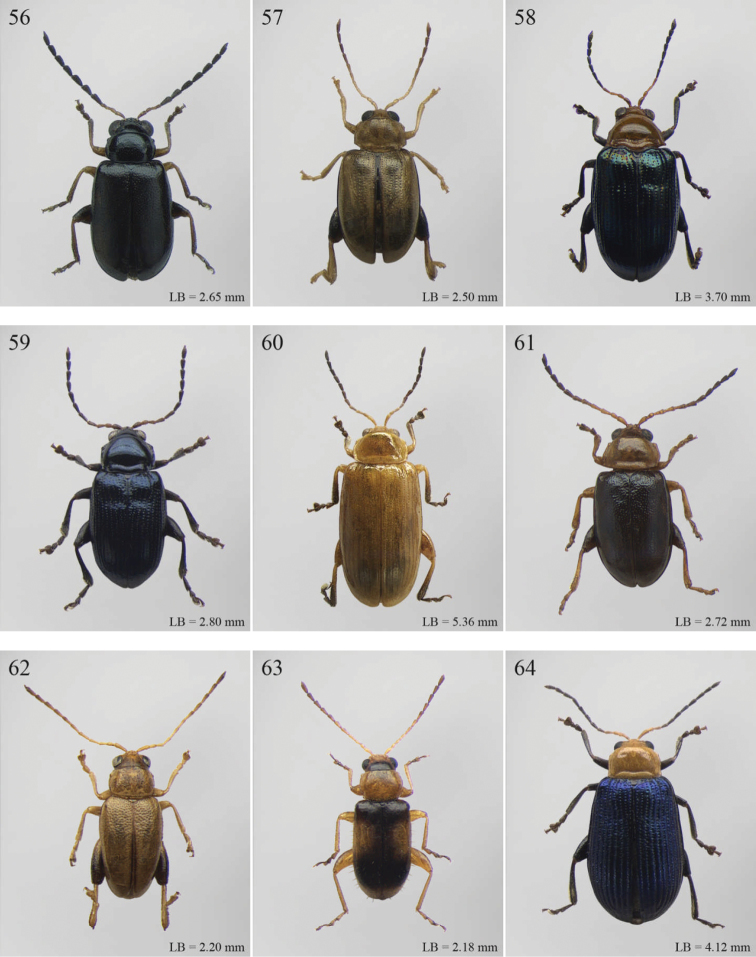
. Habitus. **56** *Kanonga atra* Bechyné **57** *Kenialtica muhavura* Bechyné **58** *Kimongona callifera* Bechyné **59** *Kimongona* (ex *Mesocrepis* Scherer) *lindemannae* (Scherer) **60** *Lampedona testacea* Bechyné **61** *Lepialtica bicolor* Scherer **62** *Longitarsus africanus* Jacoby **63** *Luperomorpha biondii* Döberl **64** *Lypnea costatipennis* ( Jacoby).

**Figures 65–73.**
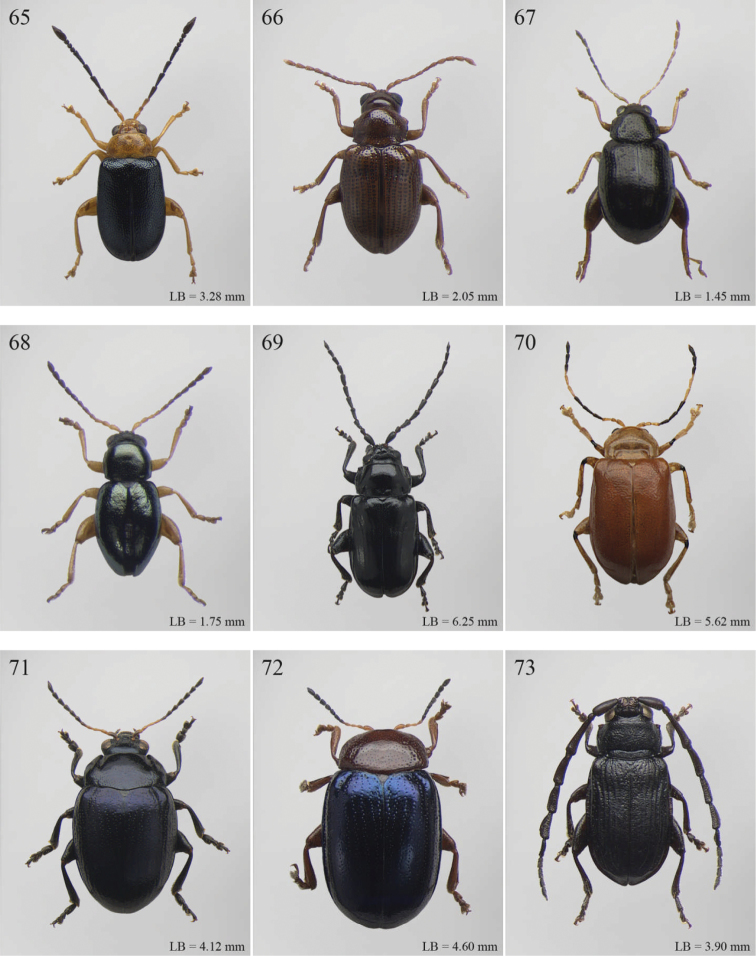
Habitus. **65** *Malvernia varicornis* Jacoby **66** *Manobia africana* Laboissière **67** *Metroserrapha* sp **68** *Montiaphthona gedyei* (Bryant) **69** *Myrcina vandenplaasi* Laboissière **70** *Neodera fraterna* Duvivier **71** *Nisotra aruwimiana* Weise **72** *Notomela fulvicollis* Bryant **73** *Ntaolaltica antennata* Biondi & D’Alessandro.

**Figures 74–82.**
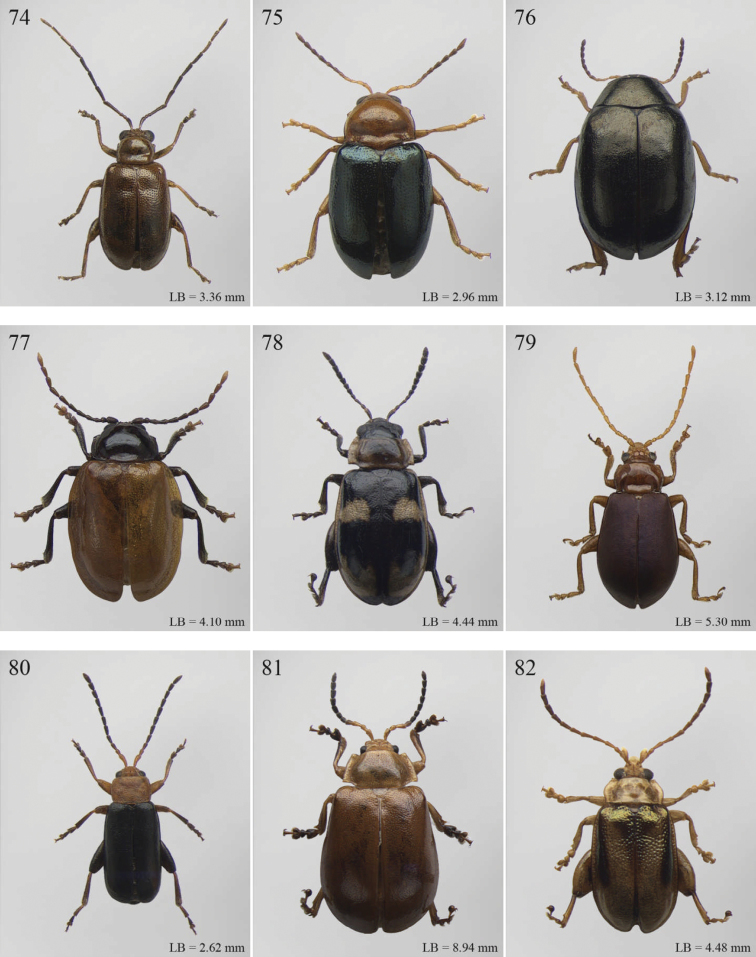
Habitus. **74** *Nzerekorena filicornis* Scherer & Boppré **75** *Orthocrepis togoensis* Weise **76** *Paradibolia abdominalis* (Jacoby) **77** *Perichilona bicolor* Weise **78** *Philopona fulvicollis* (Fabricius) **79** *Phygasia rubripennis* Weise **80** *Phyllotreta puncticollis* (Jacoby) **81** *Physodactyla rubiginosa* (Gerstaecker) **82** *Physoma costuliferum* Bechyné.

**Figures 83–91.**
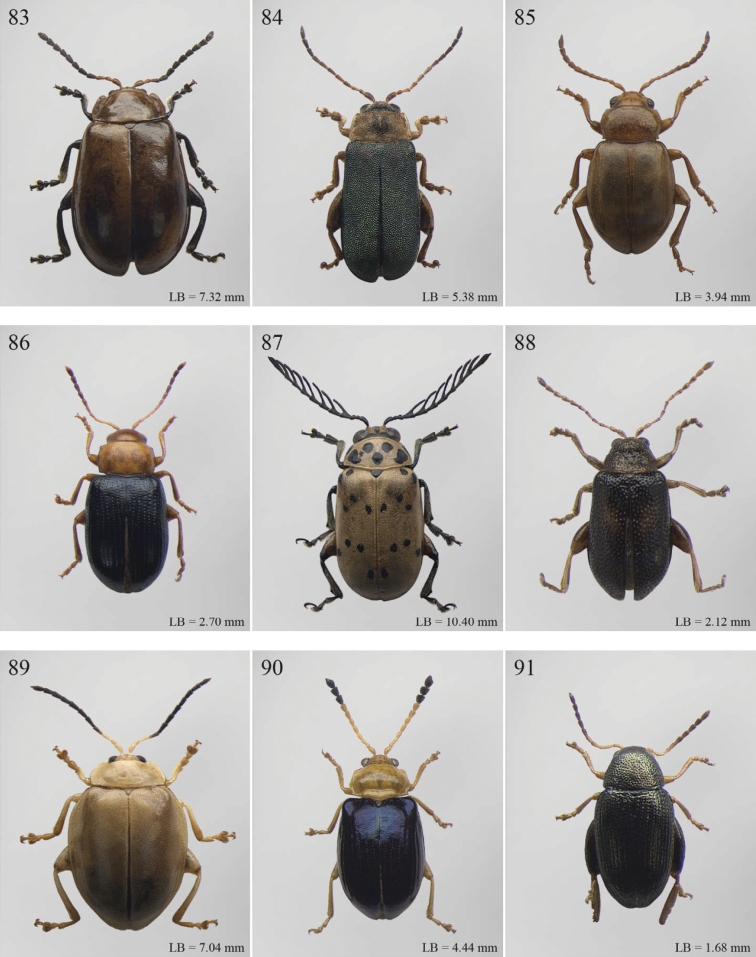
Habitus. **83** *Physomandroya melanarthra* (Fairmaire) **84** *Physonychis violaceipennis* (Baly) **85** *Podagrica oneili* (Jacoby) **86** *Podagrica* (ex *Podagricina* Csiki) *aethiopica* (Chapuis) **87** *Polyclada bohemani* (Baly) **88** *Pratima variabilis* Maulik **89** *Pseudadorium amplum* (Weise) **90** *Pseudophygasia analis* (Harold) **91** *Psylliodes calcarata* (Bryant).

**Figures 92–100.**
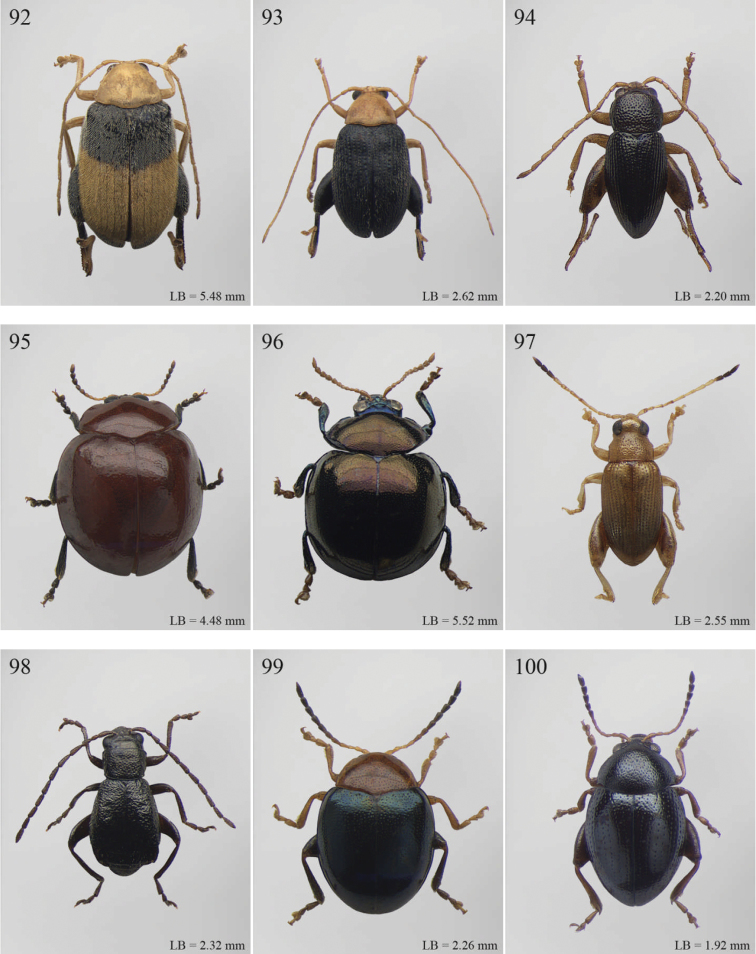
Habitus. **92** *Sanckia lebisi* Bechyné **93** *Sanckia* (ex *Eugonotes* Jacoby) *longicornis* (Jacoby) **94** *Serraphula elongata* Jacoby **95** *Sesquiphaera mashonana* (Jacoby) **96** *Sesquiphaera* (ex *Paropsiderma* Bechyné) near *vadoni* (Bechyné) **97** *Seychellaltica mahensis* (Maulik) **98** *Sjostedtinia montivaga* Weise **99 ***Sphaeroderma femoratum* Jacoby **100** *Stegnaspea trimeni* Baly.

**Figures 101–109.**
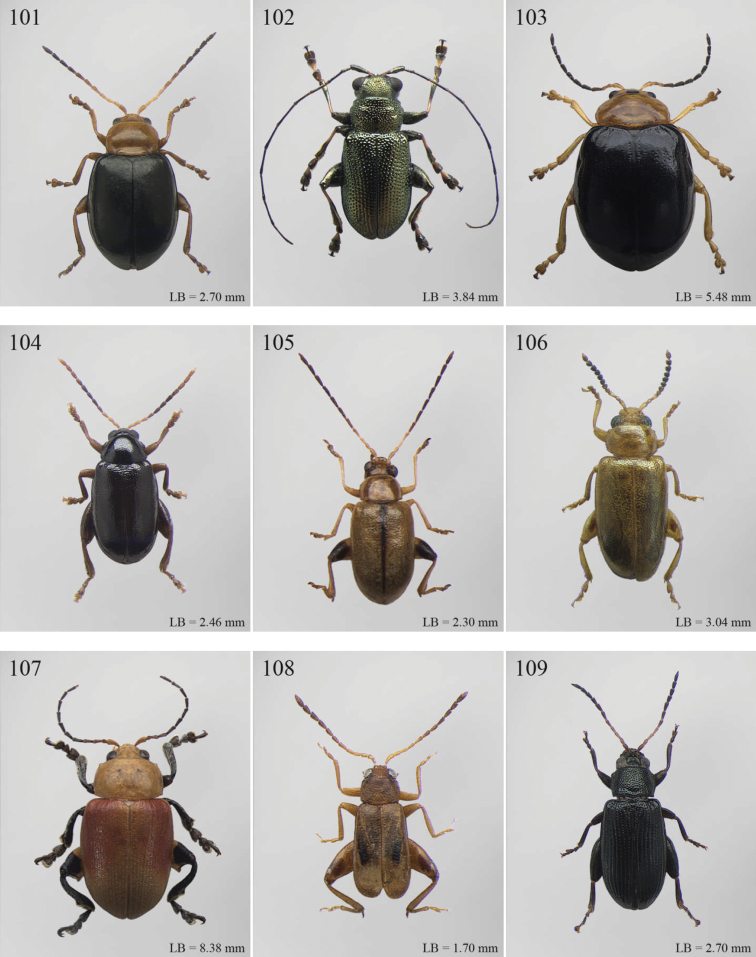
Habitus. **101** *Stuckenbergiana glabrata* (Jacoby) **102** *Terpnochlorus perrieri* Fairmaire **103** *Toxaria indica* (Fabricius) **104** *Trachytetra guineensis* (Bechyné) **105** *Tritonaphthona longicornis* (Laboissière) **106** *Upembaltica scolytina* Bechyné **107** *Xanthophysca perrieri* Fairmaire **108** *Yemenaltica scorteccii* Scherer **109** *Zomba gossypii* Bryant.

**Figures 110–115.**
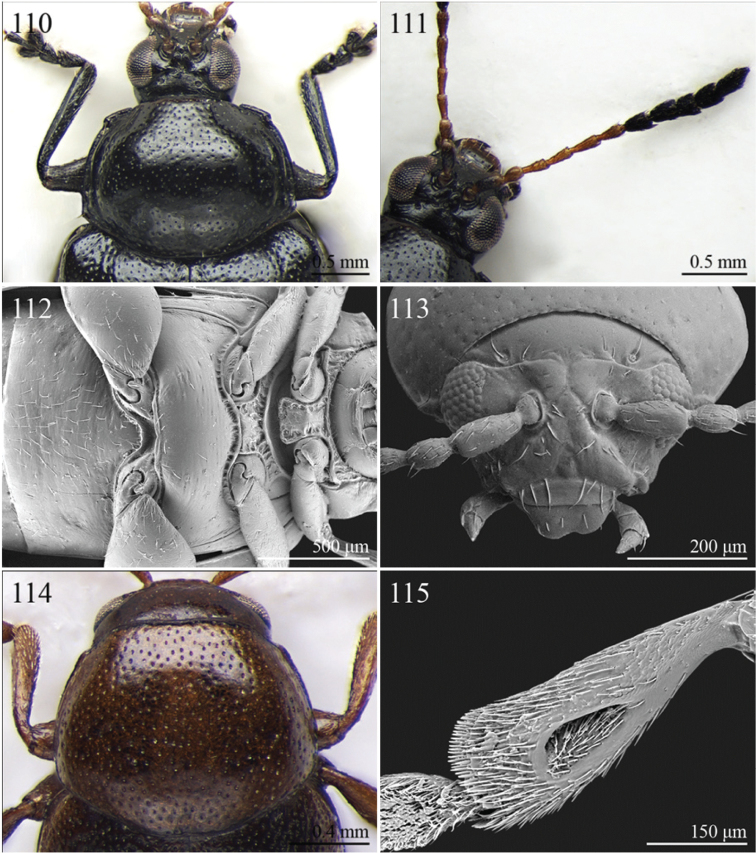
Morphological characters. **110** *Abrarius aethiops* (Weise), head and pronotum in dorsal view **111** Ditto, head and right antenna in dorsal view **112** *Abrarius cribrosus* Fairmaire, ventral parts **113** *Afroaltica parvula* D’Alessandro & Biondi, head in frontal view **114** *Afroaltica subaptera* Biondi & D’Alessandro, head and pronotum in dorsal view **115** Ditto, triangular hollow on ventral side of middle tibiae in male.

**Figures 116–121.**
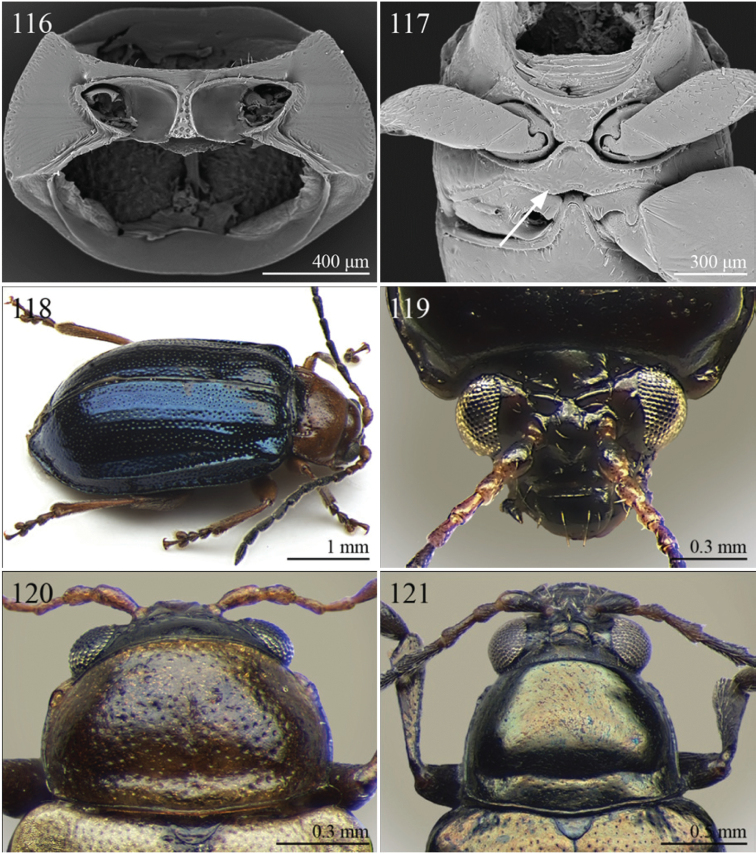
Morphological characters**. 116** *Afroaltica subaptera* Biondi & D’Alessandro, prosternum **117** Ditto, meso- and metasternum (white arrow) **118** *Afrocrepis carinipennis* (Jacoby), pronotum and elytra in sublateral view **119** *Afrorestia jonesi* (Bryant), head in frontal view **120** *Alocypha bimaculata* (Jacoby), head and pronotum in dorsal view **121** *Altica nigrita* Laboissière, head and pronotum in dorsal view.

**Figures 122–127.**
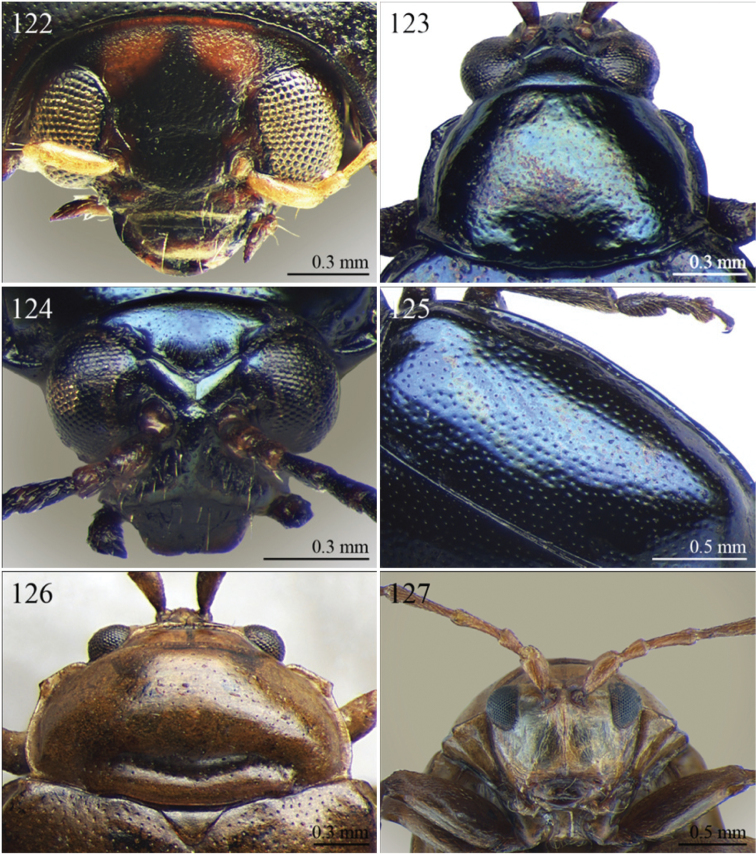
Morphological characters. **122** *Amphimela citri* (Bryant), head in frontal view **123 ***Angulaphthona latipennis* (Pic), head and pronotum in dorsal view **124** Ditto, head in frontal view **125** Ditto, elytra in dorsal view **126** *Antanemora ghesquierei* (Bechyné), head and pronotum in dorsal view **127** Ditto, head in frontal view.

**Figures 128–133.**
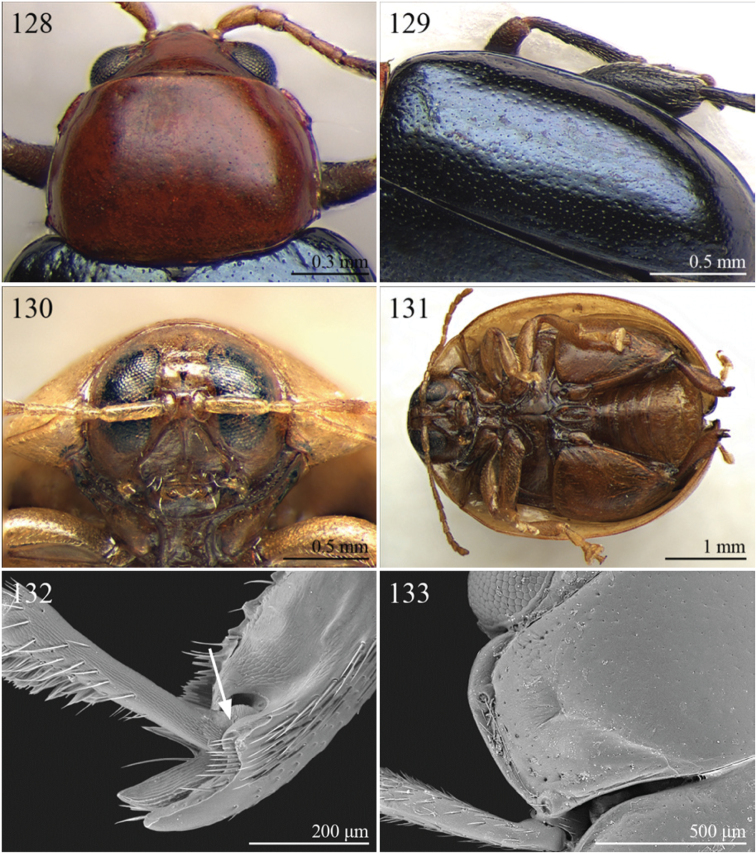
Morphological characters. **128** *Aphthona braunsi* (Jacoby), head and pronotum in dorsal view **129** Ditto, elytra in dorsal view **130** *Argopistes silvestrii* Weise, head in frontal view **131 **Ditto, ventral parts **132** *Argopistes sexvittatus* Bryant, apical part of hind tibia **133** *Argopistoides africanus* (Bryant), sublateral sulci on pronotum.

**Figures 134–139.**
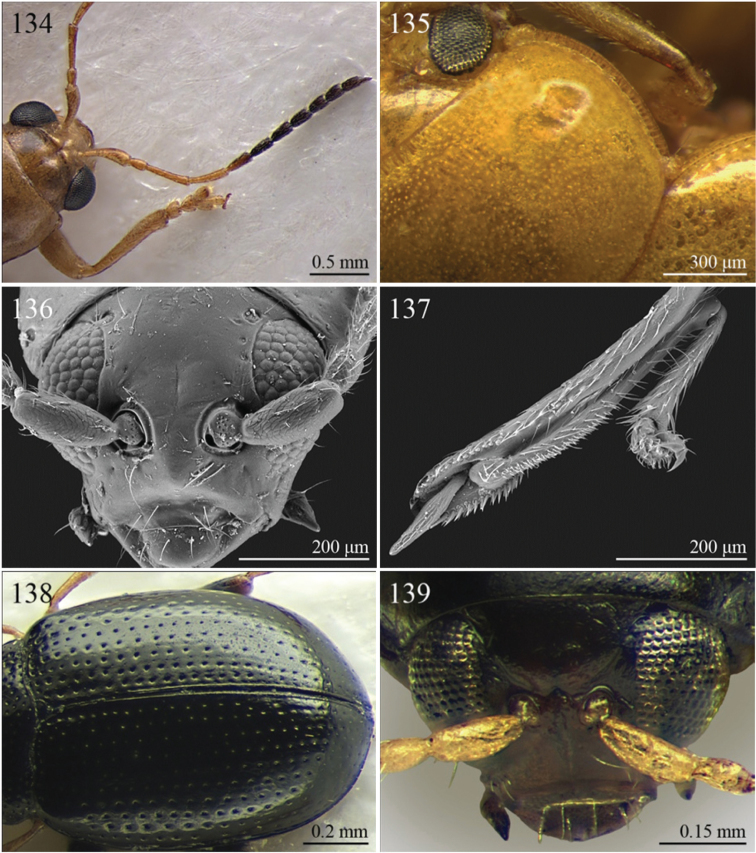
Morphological characters. **134** *Bangalaltica antennalis* Bechyné, head and right antenna in dorsal view **135** *Bechuana nigripes* Scherer, sublateral depression on pronotum **136** *Bechynella pallens* (Bechyné), head in frontal view **137** Ditto, hind tibia and metatarsus **138** *Bezdekaltica socotrana* Döberl, elytra in dorsal view **139** *Bikasha fortipunctata* Maulik, head in frontal view.

**Figures 140–145.**
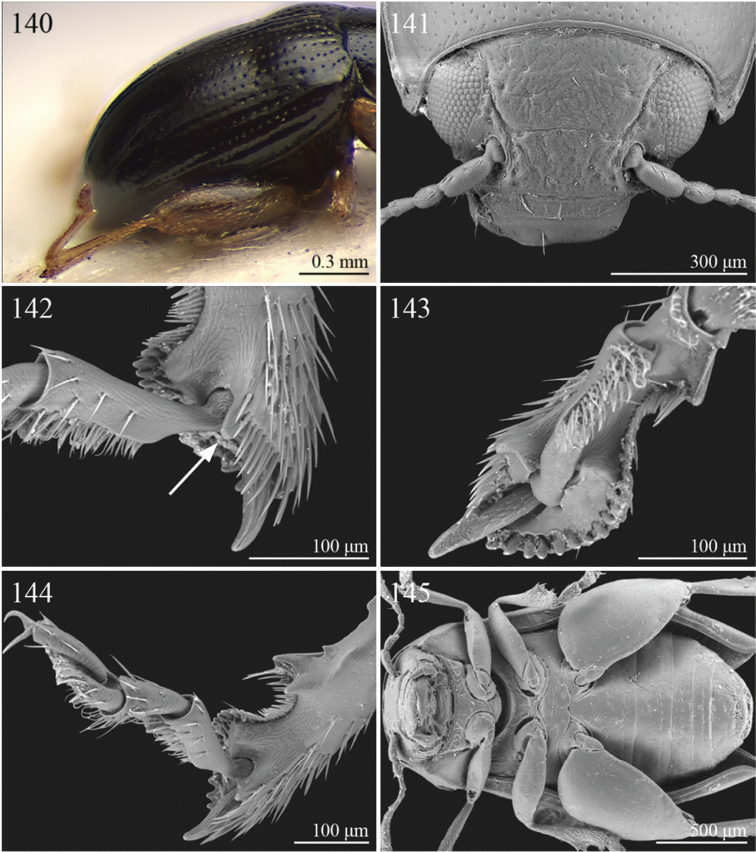
Morphological characters. **140** *Bikasha fortipunctata* Maulik, hind leg and elytra in lateral view **141** *Biodontocnema brunnea* Biondi, head in frontal view **142** Ditto, distal half of hind tibia and metatarsus **143** Ditto, hind tibial socket **144** Ditto, dentiform process (white arrow) in inner apical side of hind tibiae **145** Ditto, ventral parts.

**Figures 146–151.**
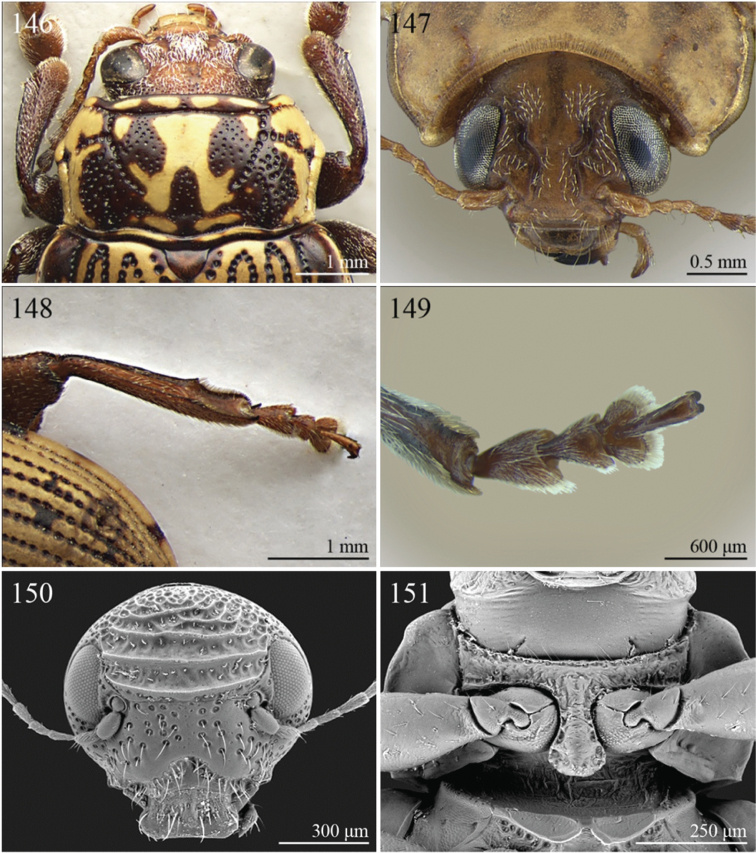
Morphological characters. **146** *Blepharida intermedia* Jacoby, head and pronotum in dorsal view **147** *Blepharida reticulata* (Baly), head in frontal view **148** *Blepharida ugandae* Bryant, hind leg **149** *Blepharida geminata* Bryant, metatarsus **150** *Carcharodis malvernensis* Bechyné, head in frontal view **151** *Celisaltica ruwenzorica* Biondi, prosternum.

**Figures 152–157.**
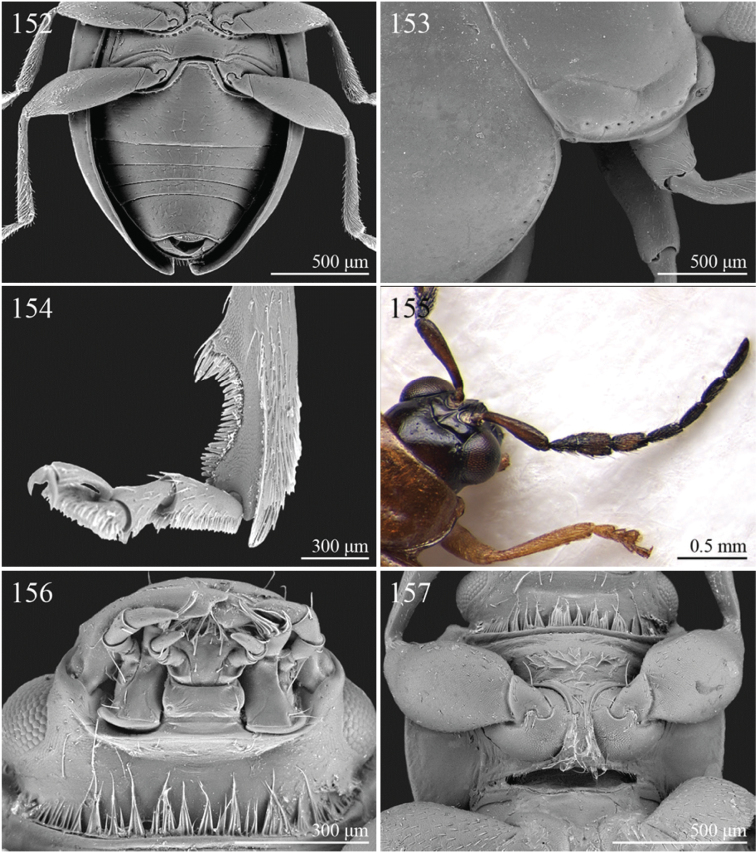
Morphological characters. **152** *Celisaltica ruwenzorica* Biondi, metasternum and abdomen **153** *Chabria betsimisaraka* Biondi & D’Alessandro, sublateral sulci on pronotum **154** *Chaetocnema sudafricana* Biondi & D’Alessandro, ciliate dentate emargination of hind tibiae **155** *Chaillucola formicicornis* Bechyné, head and right antenna in dorsal view in male **156** *Chirodica similfulva* Biondi, buccal parts **157** *Chirodica chalcoptera* Germar, prosternum and anterior femora in ventral view.

**Figures 158–163.**
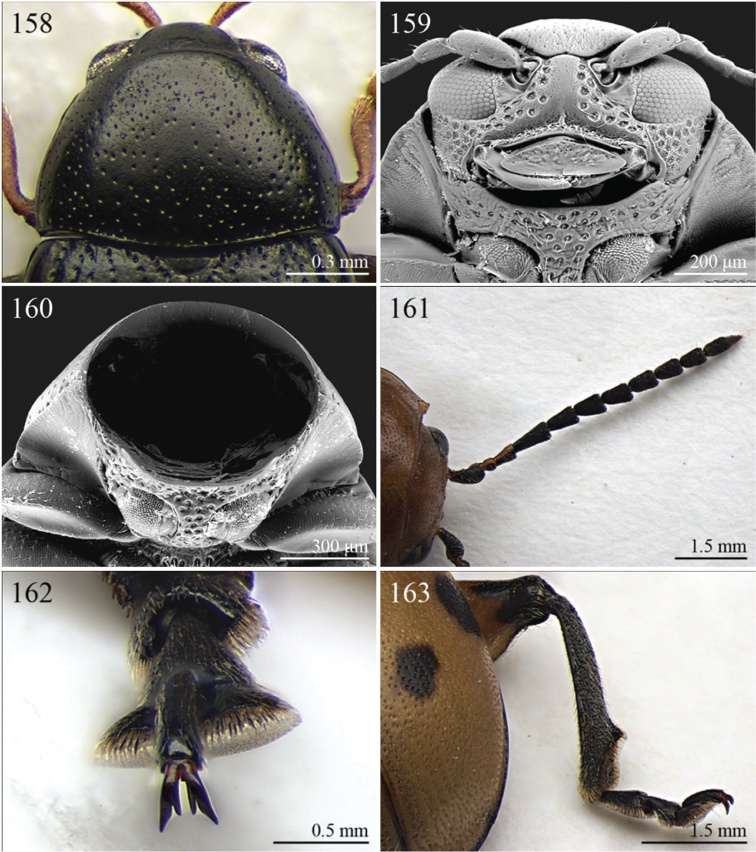
Morphological characters. **158** *Collartaltica tenebrosa* (Laboissière), head and pronotum in dorsal view **159** Ditto, head in ventral view **160** Ditto, prosternum **161** *Diamphidia femoralis* Gerstaecker, left antenna in dorsal view **162** Ditto, mesotarsus **163** *Diamphidia nigroornata* Stål, hind leg and elytral punctation.

**Figures 164–169.**
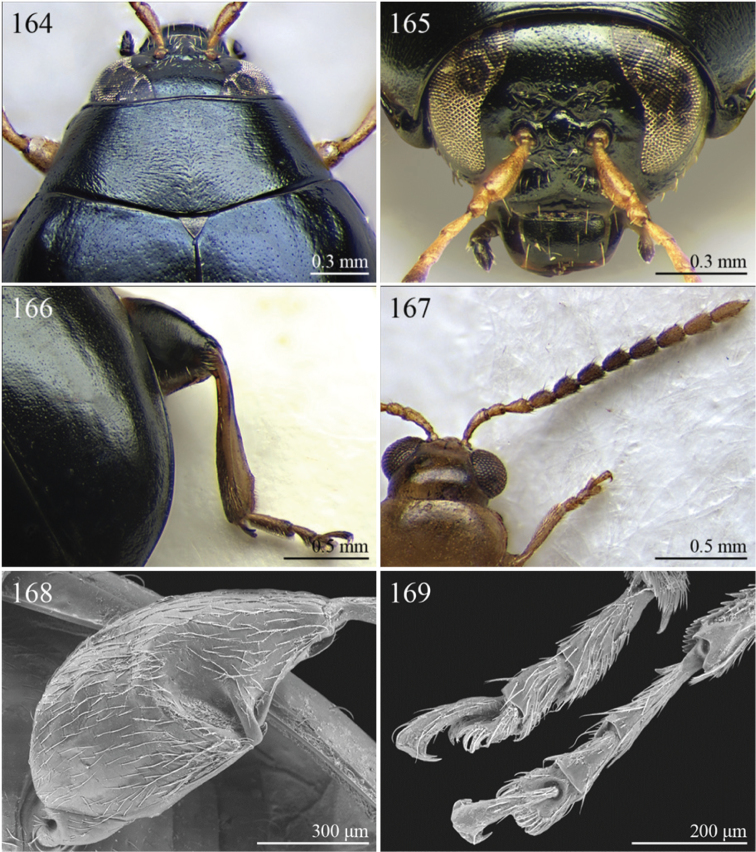
Morphological characters. **164** *Dibolia thoracica* Jacoby, head and pronotum in dorsal view **165** Ditto, head in frontal view **166** Ditto, hind leg and elytral punctation **167** *Dimonikaea descarpentriesi* Bechyné, head and right antenna in dorsal view **168** Ditto, dentiform process on ventral side of hind femora in male **169** Ditto, metatarsus in lateral (left) and dorsal (right) view.

**Figures 170–175.**
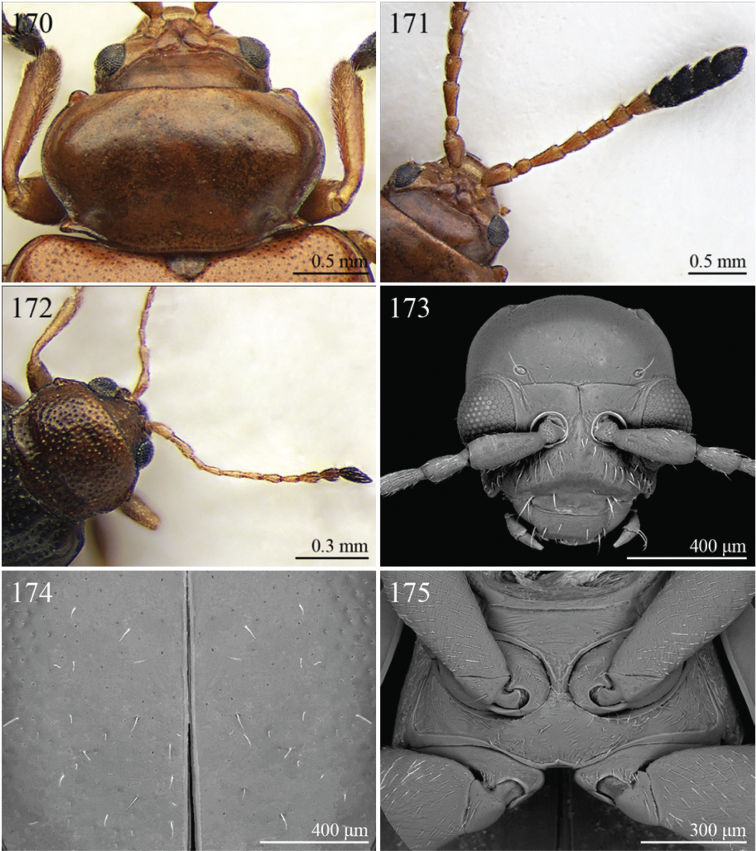
Morphological characters. **170** *Diphaulacosoma laevipenne* Jacoby, head and pronotum in dorsal view **171** Ditto, head and right antenna in dorsal view **172** *Djallonia maindra* Bechyné, head, pronotum and right antenna in dorsal view **173** *Drakensbergianella rudebecki* Biondi & D’Alessandro, head in frontal view **174** Ditto, elytral surface **175** Ditto, meso- and metasternum.

**Figures 176–181.**
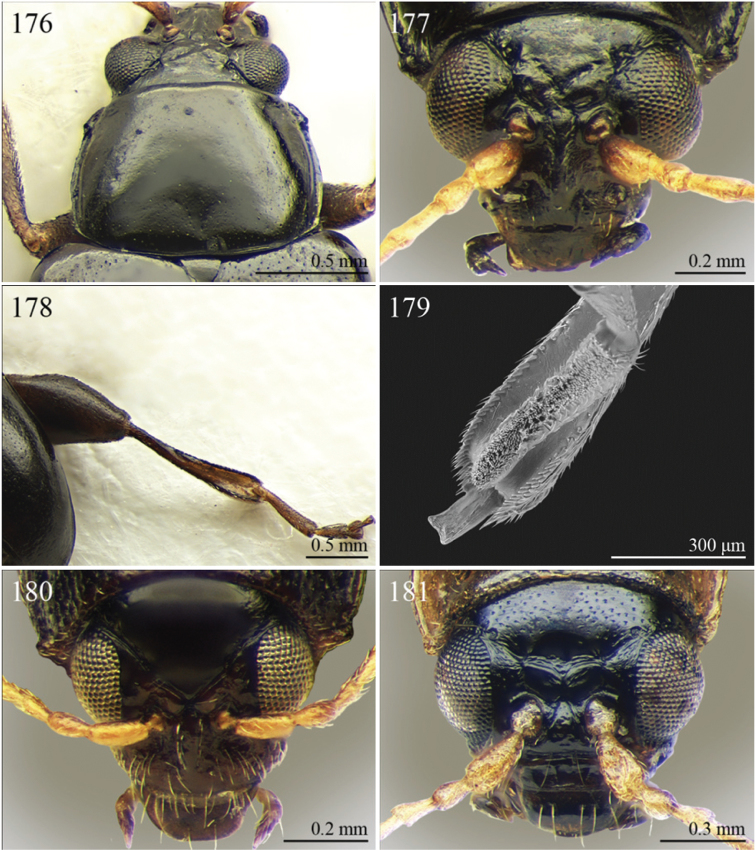
Morphological characters**. 176** *Dunbrodya nitida* Jacoby, head and pronotum in dorsal view **177** Ditto, head in frontal view **178** Ditto, hind leg **179** Ditto, bifid apical spur of hind tibiae **180** *Epitrix aethiopica* Weise, head in frontal view **181** *Eriotica fuscipennis* Harold, head in frontal view.

**Figures 182–187.**
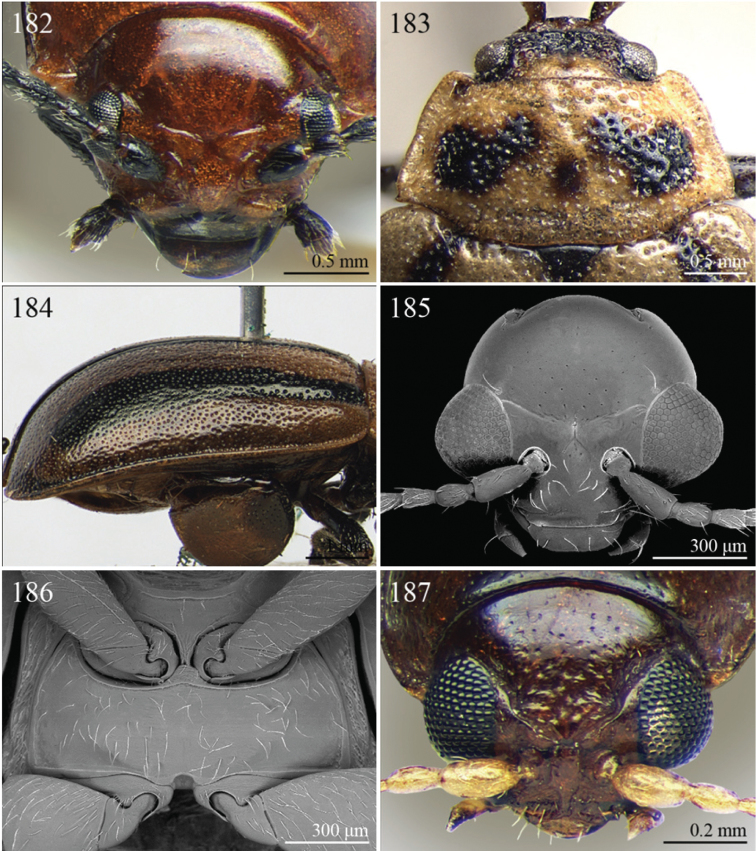
Morphological characters. **182** *Eurylegna rubra* (Weise), head in frontal view **183 ***Eutornus dilatatus* Bryant, head and pronotum in dorsal view **184** *Eutornus taeniatus* Bechyné, elytra in lateral view **185** *Gabonia nigripennis* (Jacoby), head in frontal view **186** Ditto, meso- and metasternum **187** *Guinerestia rubra* Scherer, head in frontal view.

**Figures 188–193.**
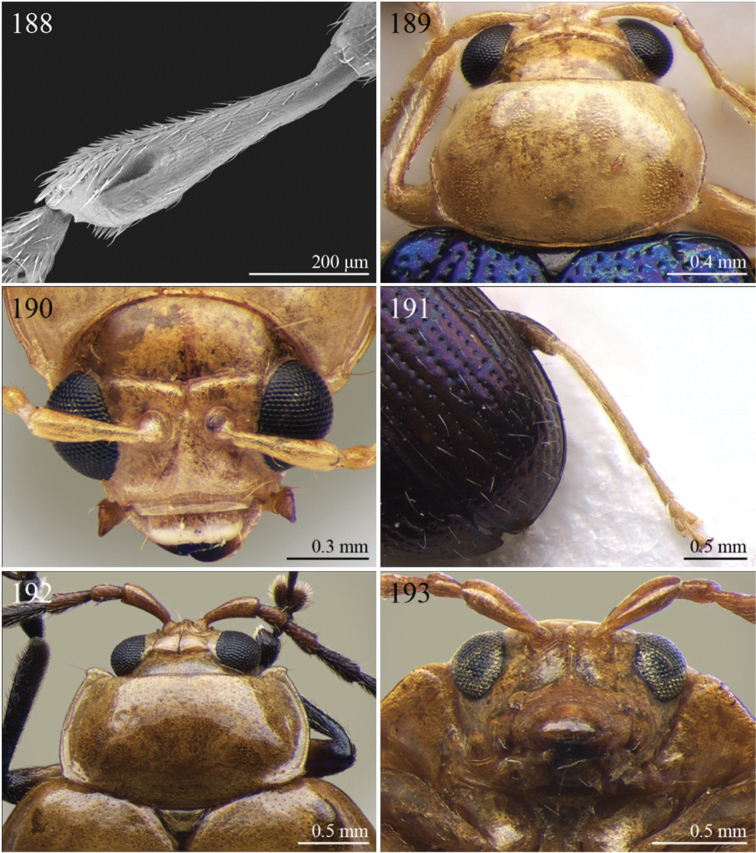
Morphological characters. **188** *Guinerestia rubra* Scherer, longitudinal hollow in distal half of anterior tibiae **189** *Halticotropis costipennis* Bechyné, head and pronotum in dorsal view **190 **Ditto, head in frontal view **191** Ditto, hind leg and elytral surface **192** *Hemipyxis burgeoni* (Laboissière), head and pronotum in dorsal view **193** Ditto, head in frontal view.

**Figures 194–199.**
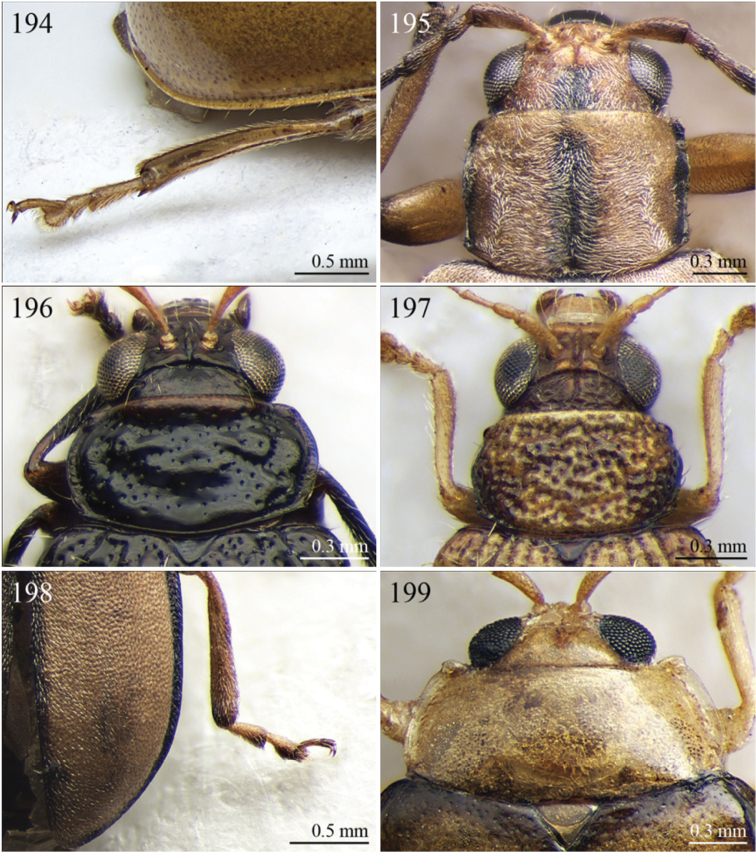
Morphological characters. **194** *Hemipyxis obscuretestaceum* (Thomson), hind tibia and metatarsus **195** *Hespera maculicollis* Jacoby, head and pronotum in dorsal view **196** *Hildebrandtina obscura* Bechyné, head and pronotum in dorsal view **197** *Hildebrandtina similis* Bechyné, head and pronotum in dorsal view **198** *Homichloda barkeri* (Jacoby), hind tibia and metatarsus and elytral surface **199 ***Hyphasis sita* (Maulik), head and pronotum in dorsal view.

**Figures 200–205.**
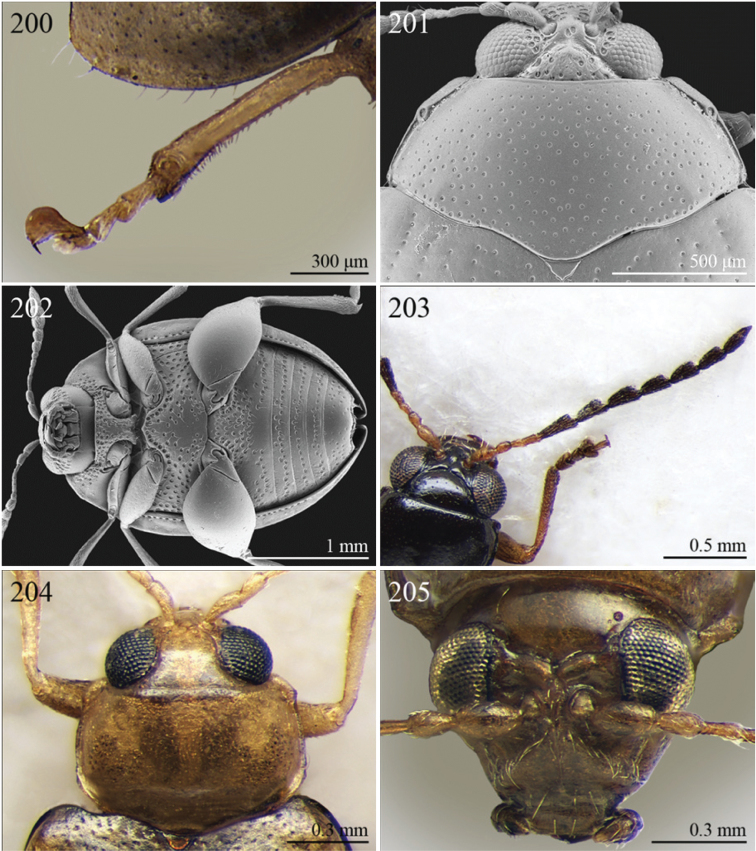
Morphological characters. **200** *Hyphasis sita* (Maulik), hind tibia and metatarsus **201** *Jacobyana bezdeki* Biondi & D’Alessandro, head and pronotum in dorsal view **202** Ditto, ventral parts **203** *Kanonga atra* Bechyné, head and right antenna in dorsal view **204** *Kenialtica muhavura* Bechyné, head and pronotum in dorsal view **205** *Kimongona callifera* Bechyné, head in frontal view.

**Figures 206–211.**
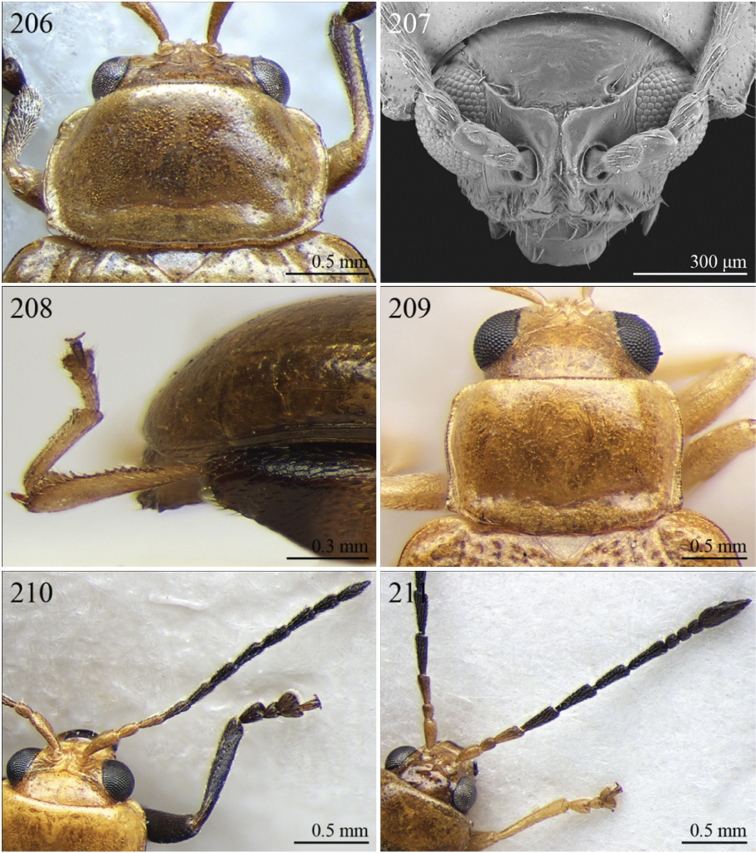
Morphological characters. **206** *Lampedona testacea* Bechyné, head and pronotum in dorsal view **207** *Lepialtica bicolor* Scherer, head in frontal view **208** *Longitarsus africanus* Jacoby, hind leg in lateral view **209** *Lypnea flaveola* (Bryant), head and pronotum in dorsal view **210** *Lypnea costatipennis* (Jacoby), head and right antenna in dorsal view **211** *Malvernia varicornis* Jacoby, head and right antenna in dorsal view.

**Figures 212–217.**
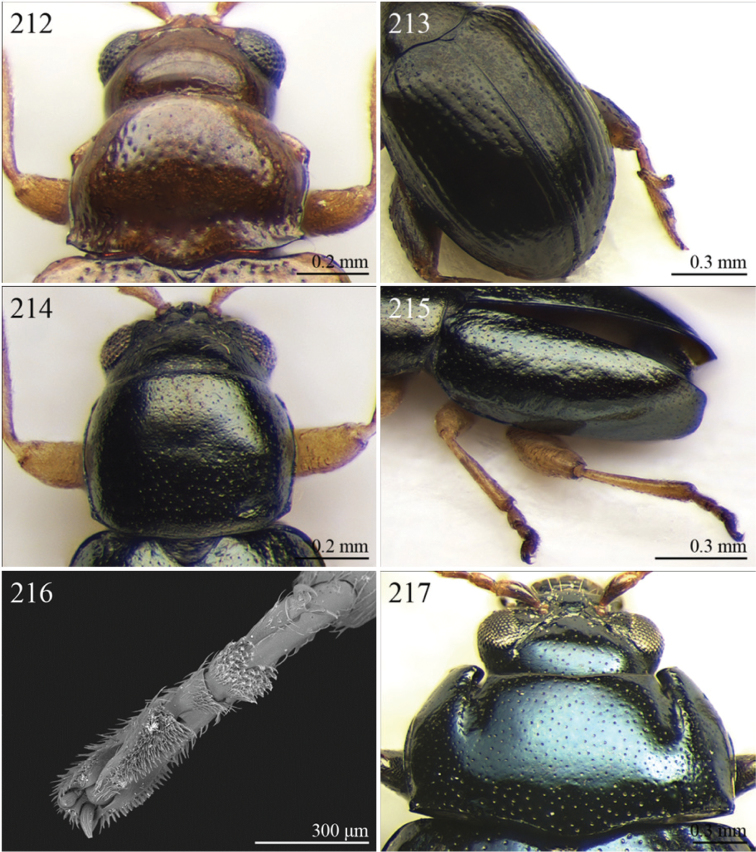
Morphological characters. **212** *Manobia africana* Laboissière, head and pronotum in dorsal view **213** *Metroserrapha* sp., hind leg and elytral punctation **214** *Montiaphthona gedyei* (Bryant), head and pronotum in dorsal view **215** Ditto, elytra in sublateral view **216** *Myrcina vandenplaasi* Laboissiére, double apical spur of hind tibia **217** *Nisotra aruwimiana* Weise, head and pronotum in dorsal view.

**Figures 218–223.**
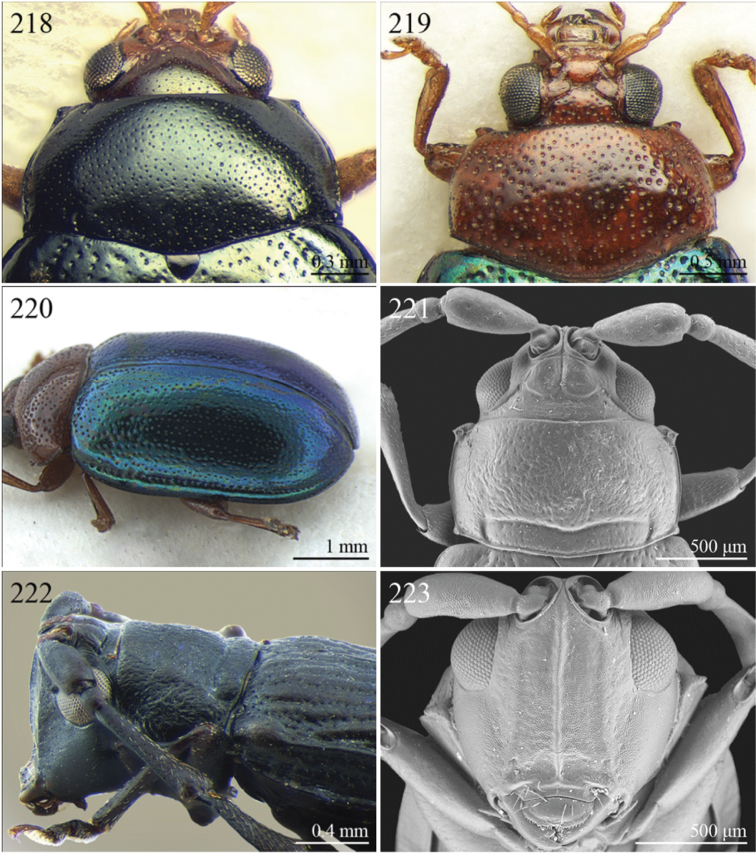
Morphological characters. **218** *Nisotra* sp., head and pronotum in dorsal view **219 ***Notomela viridipennis* Bryant, head and pronotum in dorsal view **220** Ditto, elytra in lateral view **221** *Ntaolaltica antennata* Biondi & D’Alessandro, head and pronotum in dorsal view **222** Ditto, head and pronotum in lateral view **223** Ditto, head in frontal view.

**Figures 224–229.**
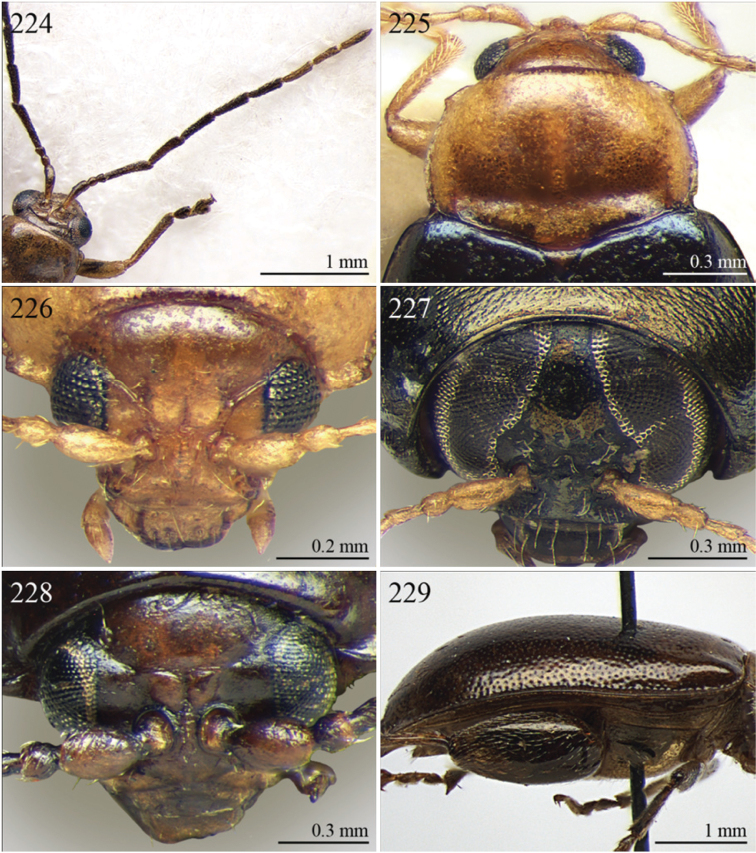
Morphological characters. **224** *Nzerekorena filicornis* Scherer & Boppré, head and right antenna in dorsal view **225** *Orthocrepis kibonotensis* (Weise), head and pronotum in lateral view **226 **Ditto, head in frontal view **227** *Paradibolia abdominalis* (Jacoby), head in frontal view **228** *Perichilona bicolor* Weise, head in frontal view **229** *Philopona sulcicollis* (Jacoby), elytra in lateral view.

**Figures 230–235.**
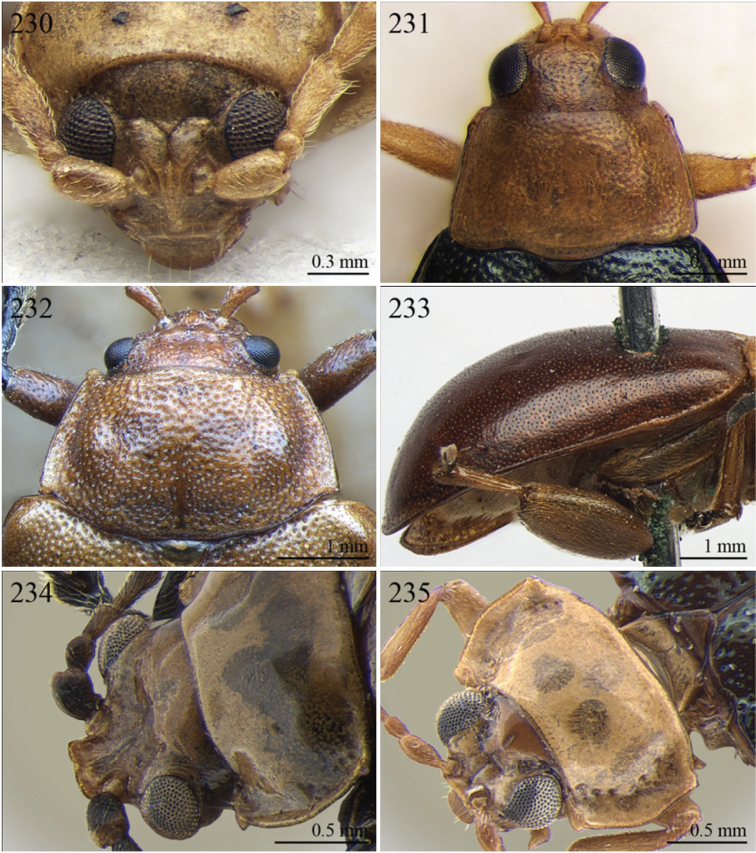
Morphological characters. **230** *Phygasia sulphuripennis* Jacoby, head in frontal view **231** *Phyllotreta natalensis* Jacoby, head and pronotum in lateral view **232** *Physodactyla rubiginosa* (Gerstaecker), head and pronotum in lateral view **233** *Physodactyla gerstaeckeri* (Jacoby), elytra in lateral view **234** *Physoma atripes* (Fairmaire), head in sublateral view **235** *Physoma costuliferum* Bechyné, head in sublateral view.

**Figures 236–241.**
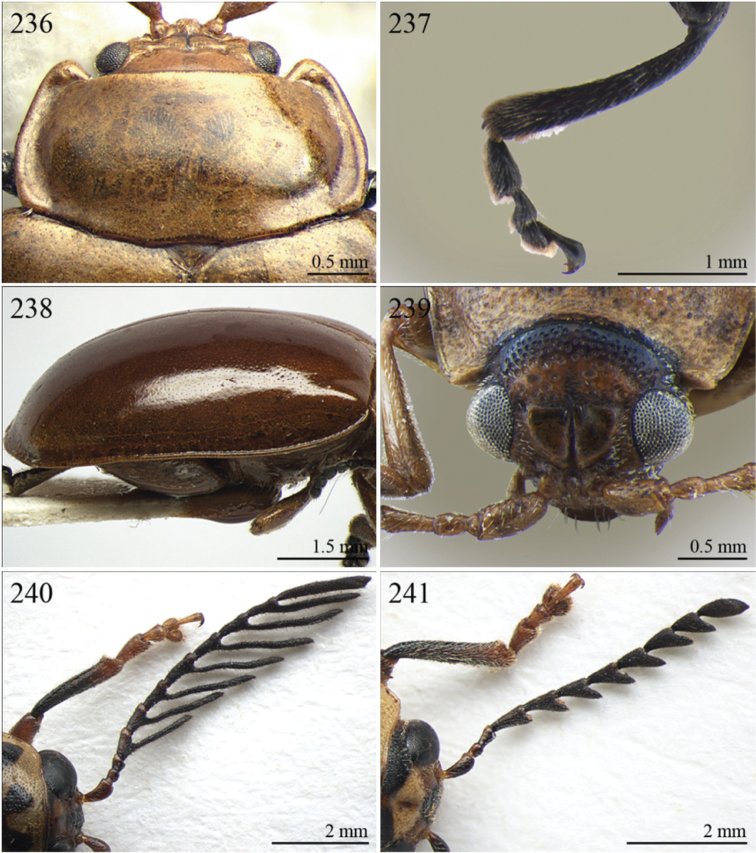
Morphological characters. **236** *Physomandroya melanarthra* (Fairmaire), head and pronotum in dorsal view **237** Ditto, hind tibia and metatarsus **238** *Physomandroya punctulifera* Bechyné, elytra in lateral view **239** *Physonychis viridipennis* (Dalman), head in frontal view **240** *Polyclada smythi* Bryant, left antenna in male in dorsal view **241** Ditto, left antenna in female in dorsal view.

**Figures 242–247.**
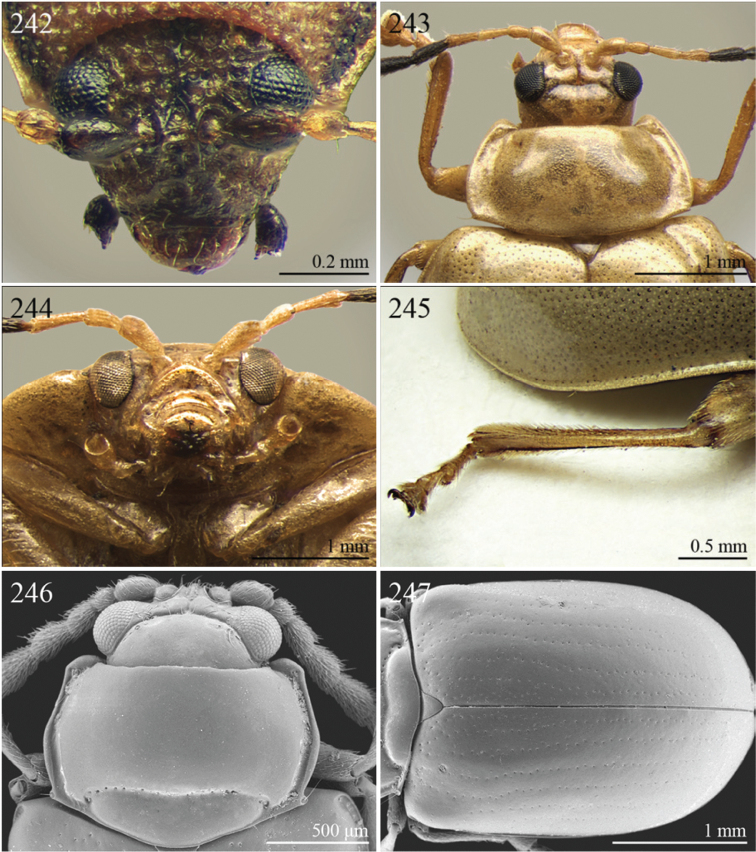
Morphological characters **242** *Pratima vinsoni* Bechyné, head in frontal view **243 ***Pseudadorium balyanum* (Csiki), head and pronotum in dorsal view **244** Ditto, head in frontal view **245** Ditto, hind leg in sublateral view **246** *Pseudophygasia analis* (Harold), head and pronotum in dorsal view **247** Ditto, elytral punctation.

**Figures 248–253.**
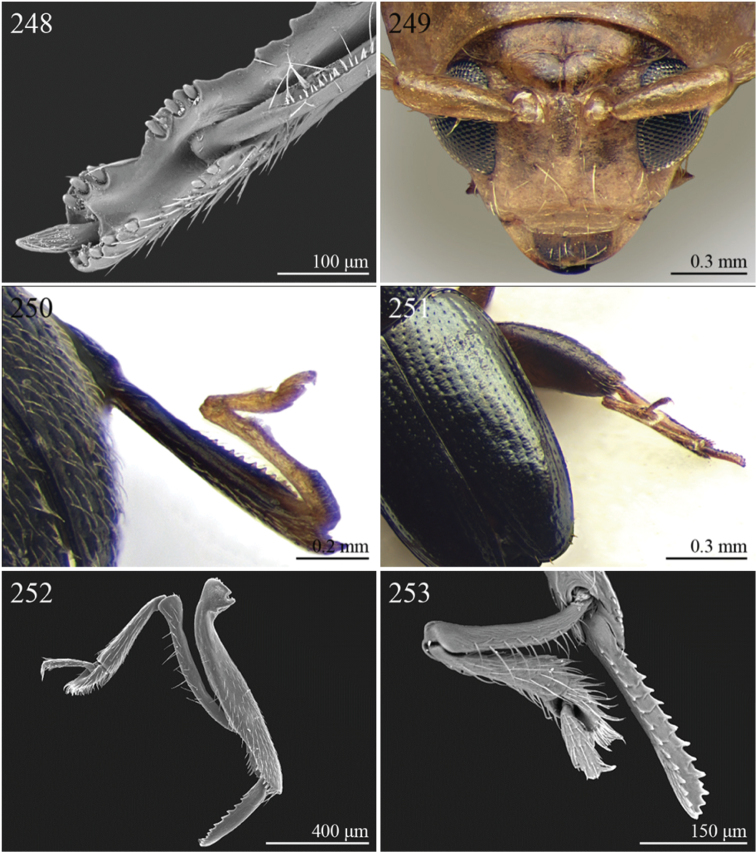
Morphological characters. **248** *Psylliodes teresae* Biondi, apical part of hind tibia and preapical insertion of metatarsus **249** *Sanckia* sp., head in frontal view **250** *Sanckia longicornis* (Jacoby), hind leg **251** *Serraphula grobbelaariae* Biondi & D’Alessandro, hind leg and elytral punctation **252** *Serraphula elongata* Jacoby, hind leg **253** *Serraphula audisiana* Biondi & D’Alessandro, serrate apical spur of hind tibiae.

**Figures 254–259.**
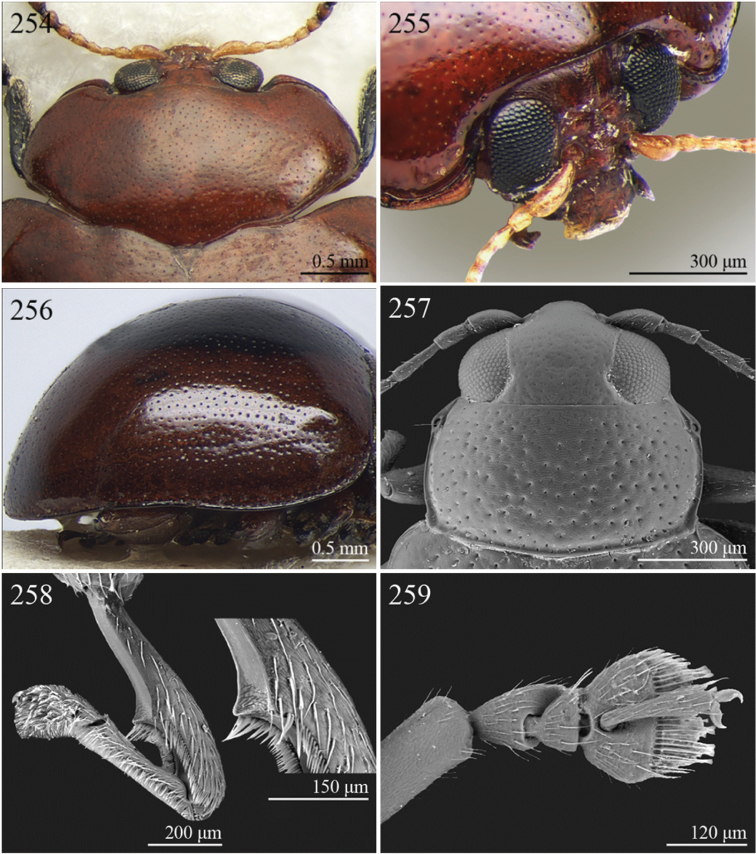
Morphological characters. **254** *Sesquiphaera mashonana* (Jacoby), head and pronotum in dorsal view **255** Ditto, head and anterior margin of pronotum in subfrontal view **256** *Sesquiphaera nigrosignata* (Bryant), elytra in lateral view **257** *Seychellaltica krishna* (Maulik), head and pronotum in dorsal view **258** Ditto, hind tibia and metatarsus **259** Ditto, protarsus in dorsal view.

**Figures 260–265.**
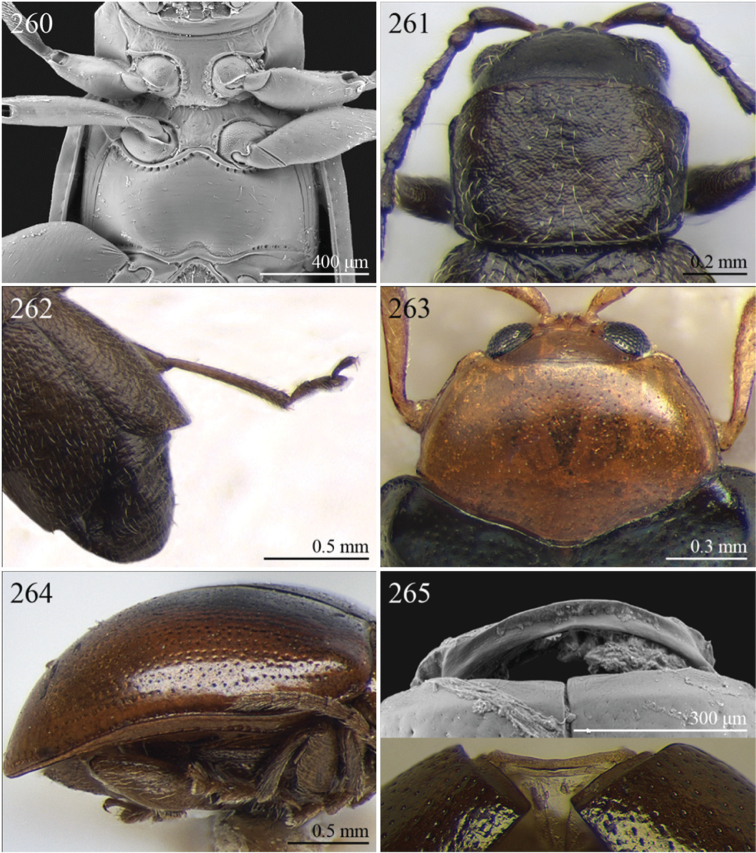
Morphological characters. **260** *Seychellaltica mahensis* (Maulik), pro-, meso- and metasternum **261** *Sjostedtinia fordi* Bryant, head and pronotum in dorsal view **262** Ditto, hind leg and elytra in dorsal view **263** *Sphaeroderma nigripennis* Bryant, head and pronotum in dorsal view **264 ***Sphaeroderma iyengari* Bechyné, elytra in lateral view **265** *Stegnaspea trimeni* Baly, basal part of elytra.

**Figures 266–271.**
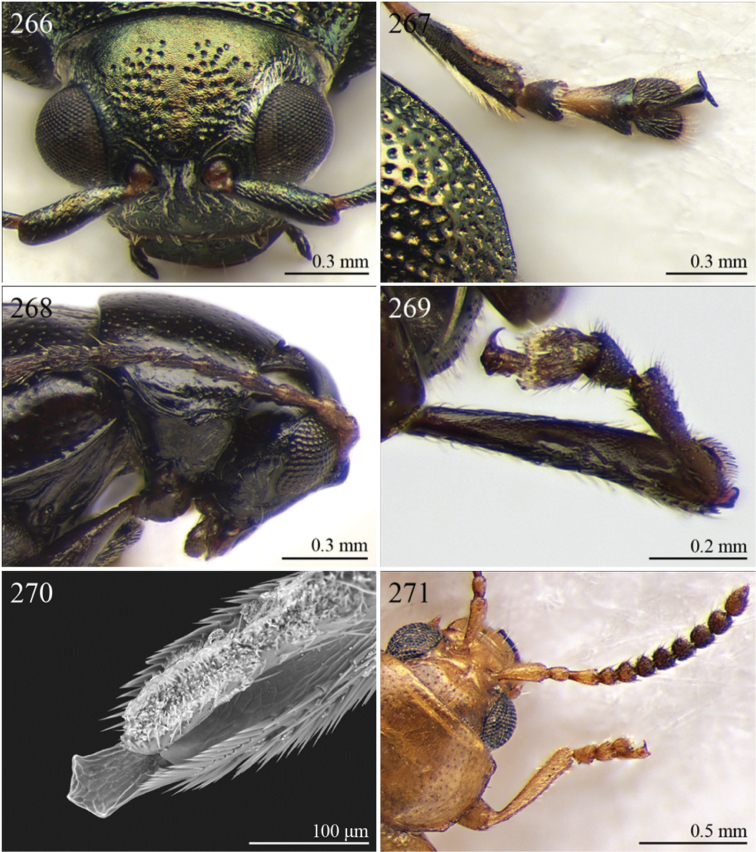
Morphological characters. **266** *Terpnochlorus perrieri* Fairmaire, head in subfrontal view **267** Ditto, metatarsus in dorsal view **268** *Trachytetra guineensis* (Bechyné), head and pronotum in lateral view **269** Ditto, hind tibia, metatarsus and apical spur **270** *Tritonaphthona longicornis* (Laboissière), trifid apical spur of hind tibiae **271** *Upembaltica scolytina* Bechyné, head and right antenna in dorsal view.

**Figures 272–277.**
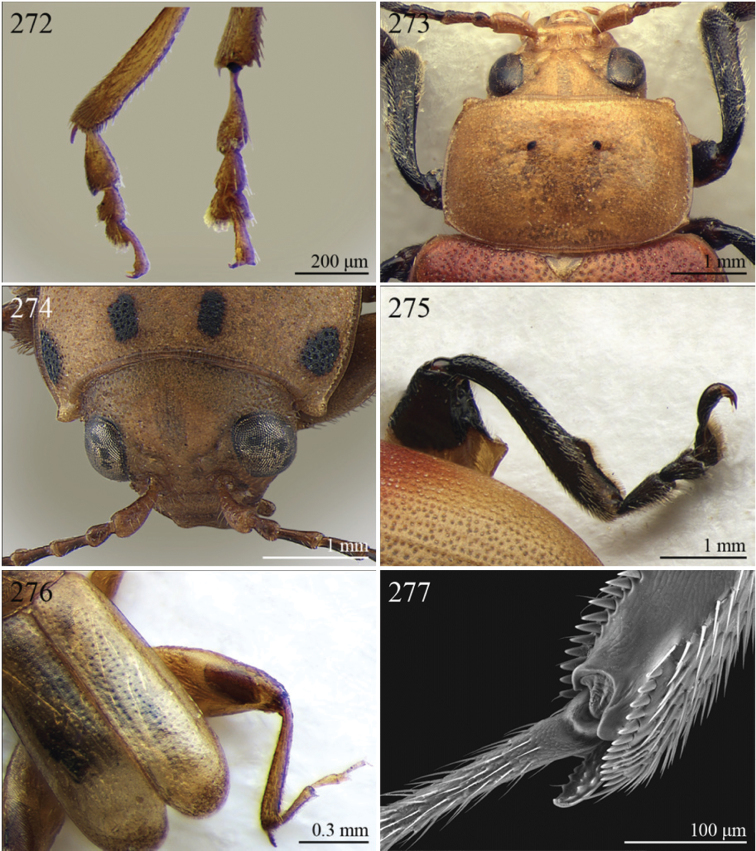
Morphological characters. **272** *Upembaltica scolytina* Bechyné, metatarsus in lateral (left) and dorsal (right) view **273** *Xanthophysca perrieri* Fairmaire, head and pronotum in dorsal view **274** Ditto, head in subfrontal view **275** Ditto, hind leg and elytral punctation **276** *Yemenaltica scorteccii* Scherer, hind leg and elytral punctation **277** *Yemenaltica furthi* Döberl, serrate apical spur of hind tibiae.

**Figures 278–283.**
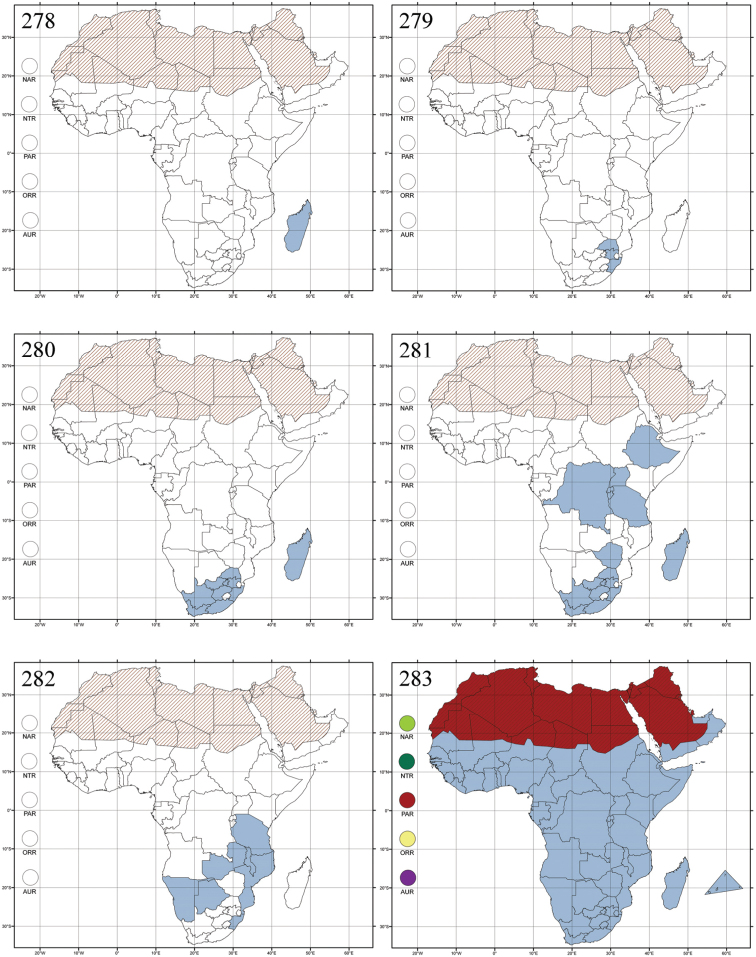
Maps of distribution. **278** *Abrarius* Fairmaire **279** *Afroaltica* Biondi & D’Alessandro **280** *Afrocrepis* Bechyné **281** *Afrorestia* Bechyné **282** *Alocypha* Weise **283** *Altica* Geoffroy.

**Figures 284–289.**
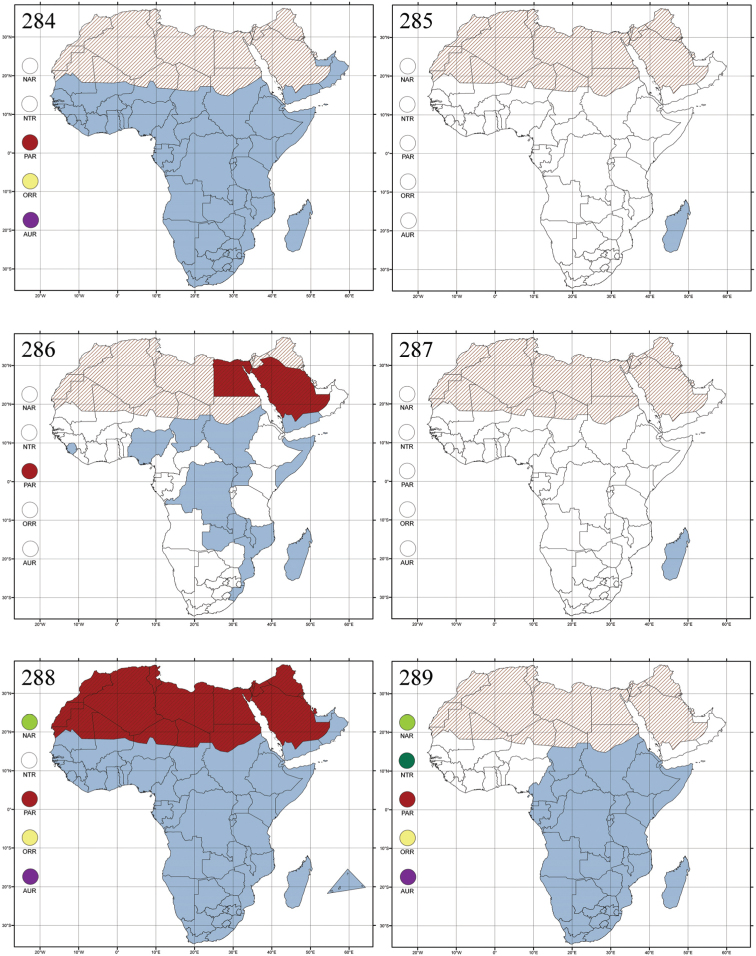
Maps of distribution. **284** *Amphimela* Chapuis **285** *Anaxerta* Fairmaire **286** *Angulaphthona* Bechyné **287** *Antanemora* Bechyné **288** *Aphthona* Chevrolat **289** *Argopistes* Motschulsky.

**Figures 290–295.**
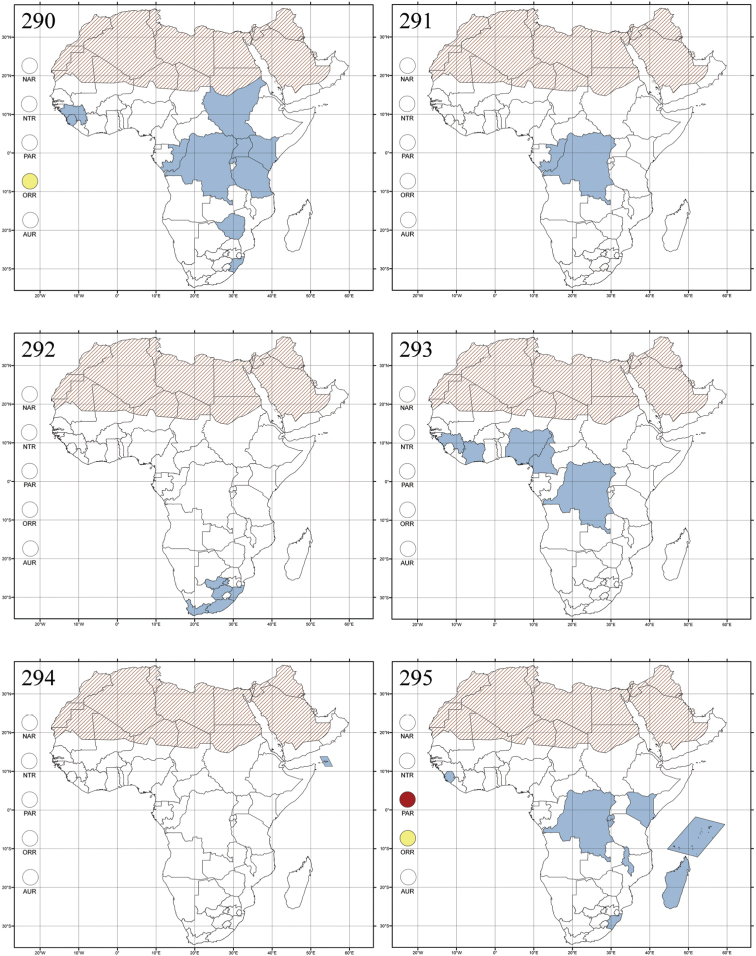
Maps of distribution. **290** *Argopistoides* Jacoby **291** *Bangalaltica* Bechyné **292** *Bechuana* Scherer **293** *Bechynella* Biondi & D’Alessandro **294** *Bezdekaltica* Döberl **295** *Bikasha* Maulik.

**Figures 296–301.**
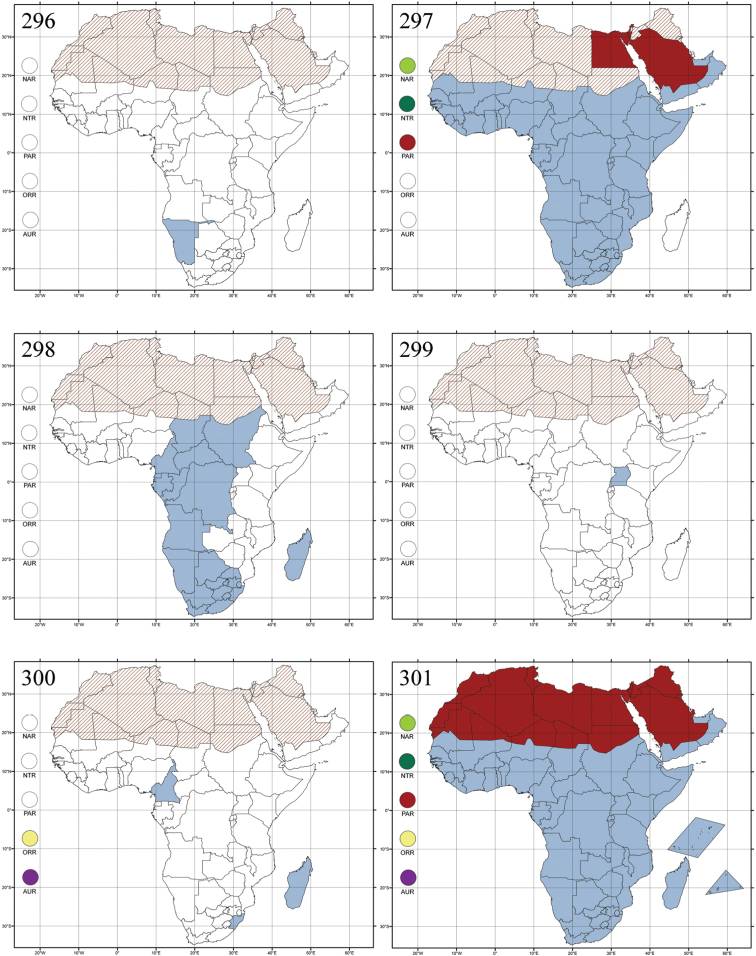
Maps of distribution. **296** *Biodontocnema* Biondi **297** *Blepharida* Chevrolat **298 ***Carcharodis* Weise **299** *Celisaltica* Biondi **300** *Chabria* Jacoby **301** *Chaetocnema* Stephens.

**Figures 302–307.**
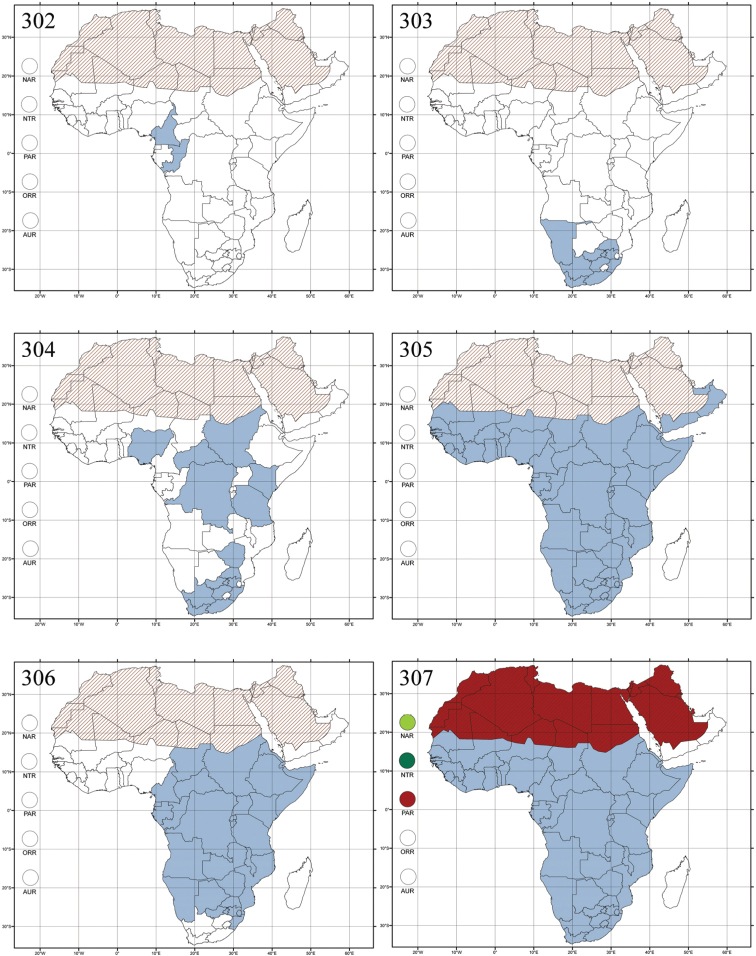
Maps of distribution. **302** *Chaillucola* Bechyné **303** *Chirodica* Germar **304** *Collartaltica* Bechyné **305** *Decaria* Weise **306** *Diamphidia* Gerstaecker **307** *Dibolia* Latreille.

**Figures 308–313.**
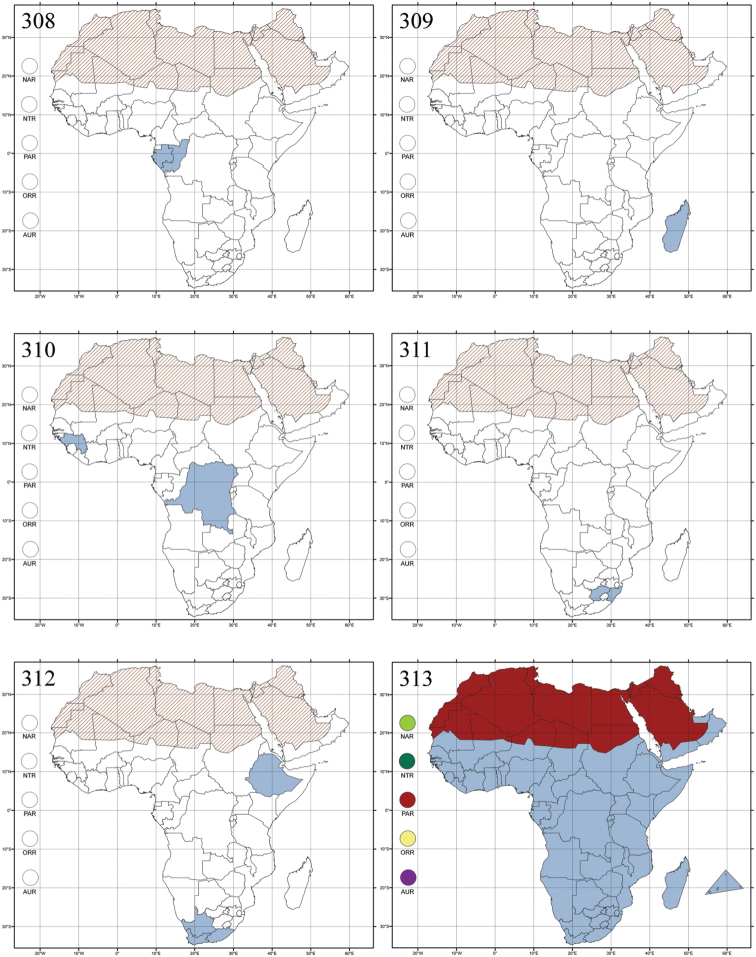
Maps of distribution. **308** *Dimonikaea* Bechyné **309** *Diphaulacosoma* Jacoby **310** *Djallonia* Bechyné **311** *Drakensbergianella* Biondi & D’Alessandro **312** *Dunbrodya* Jacoby **313** *Epitrix* Foudras.

**Figures 314–319.**
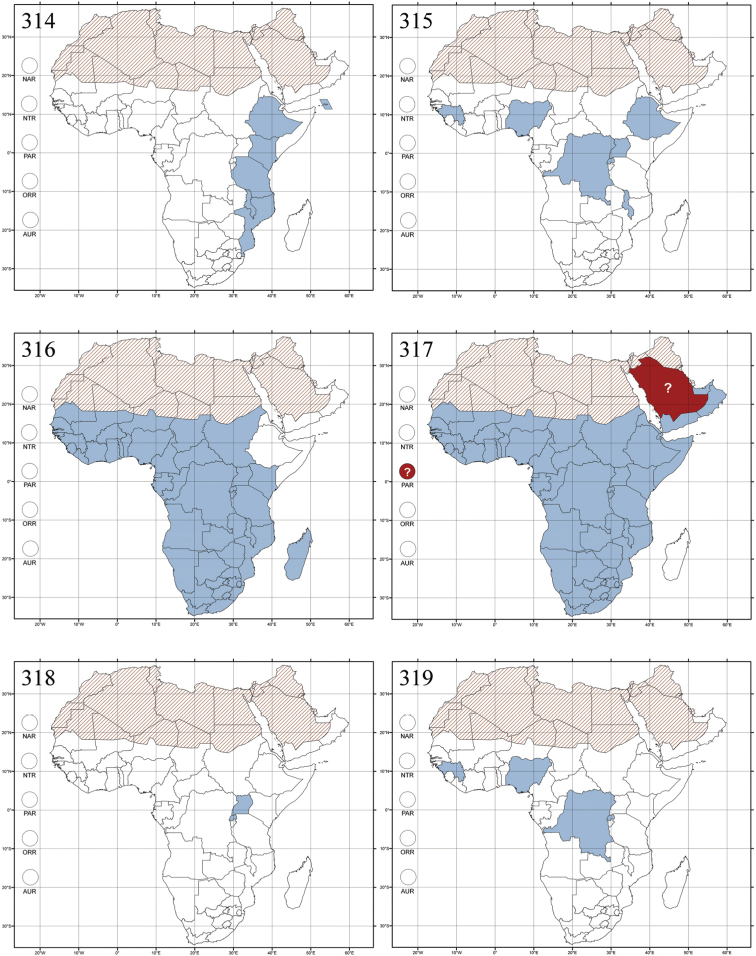
Maps of distribution. **314** *Eriotica* Harold **315** *Eurylegna* Weise **316** *Eutornus* Clark **317** *Gabonia* Jacoby **318** *Guilielmia* Weise **319** *Guinerestia* Scherer.

**Figures 320–325.**
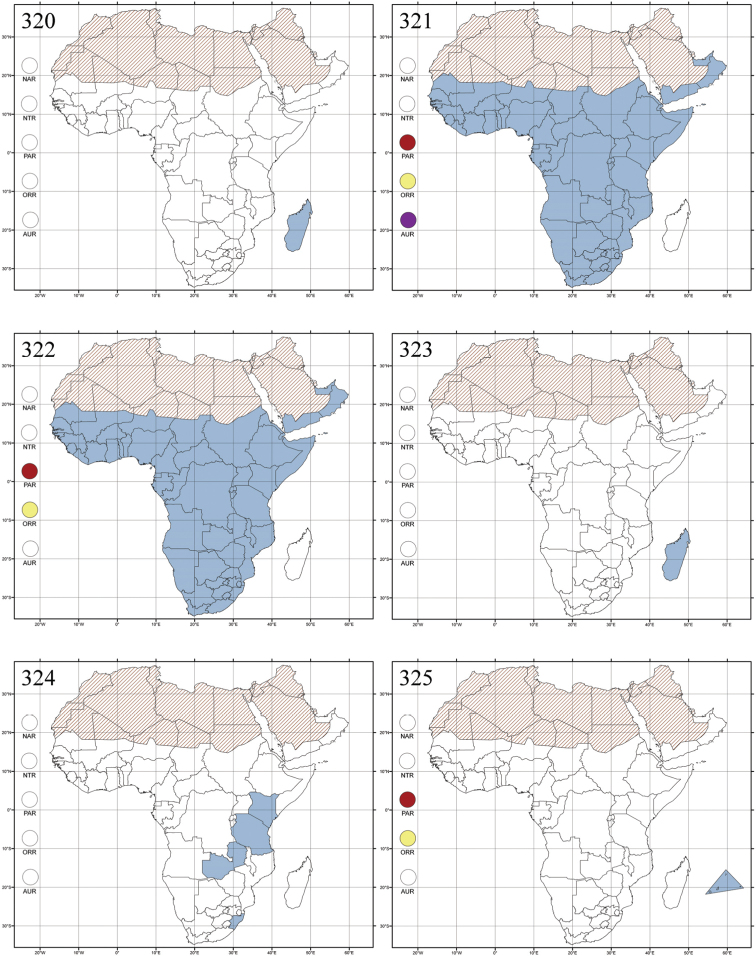
Maps of distribution. **320** *Halticotropis* Fairmaire **321** *Hemipyxis* Chevrolat **322 ***Hespera* Weise **323** *Hildebrandtina* Weise **324** *Homichloda* Weise **325** *Hyphasis* Harold.

**Figures 326–331.**
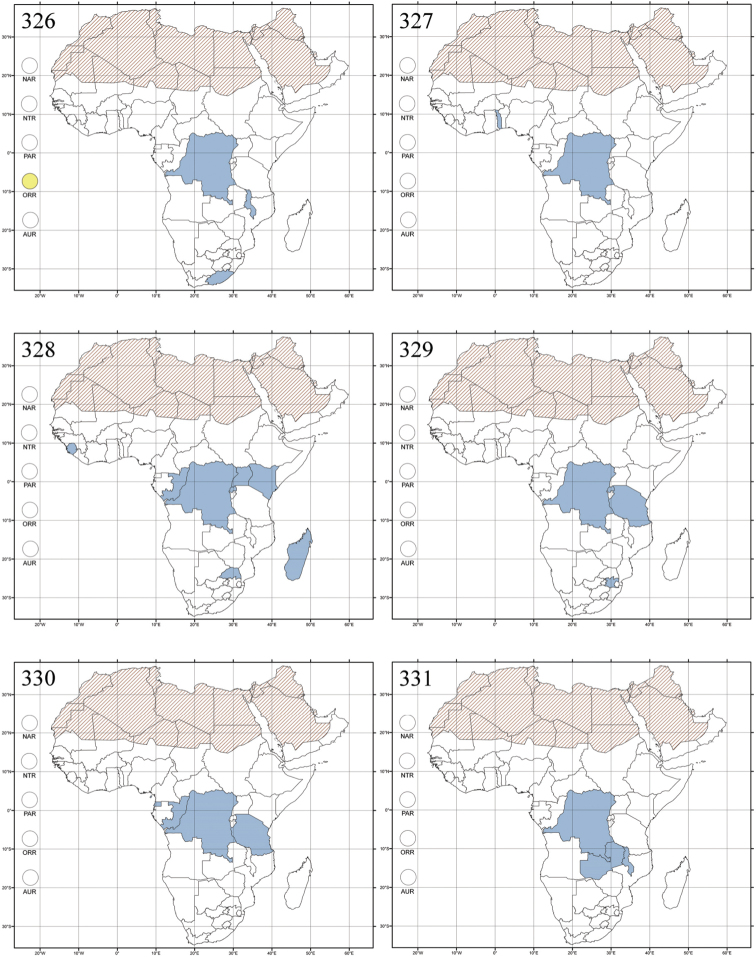
Maps of distribution. **326** *Jacobyana* Maulik **327** *Kanonga* Bechyné **328** *Kenialtica* Bechyné **329** *Kimongona* Bechyné **330** *Lampedona* Weise **331** *Lepialtica* Scherer.

**Figures 332–337.**
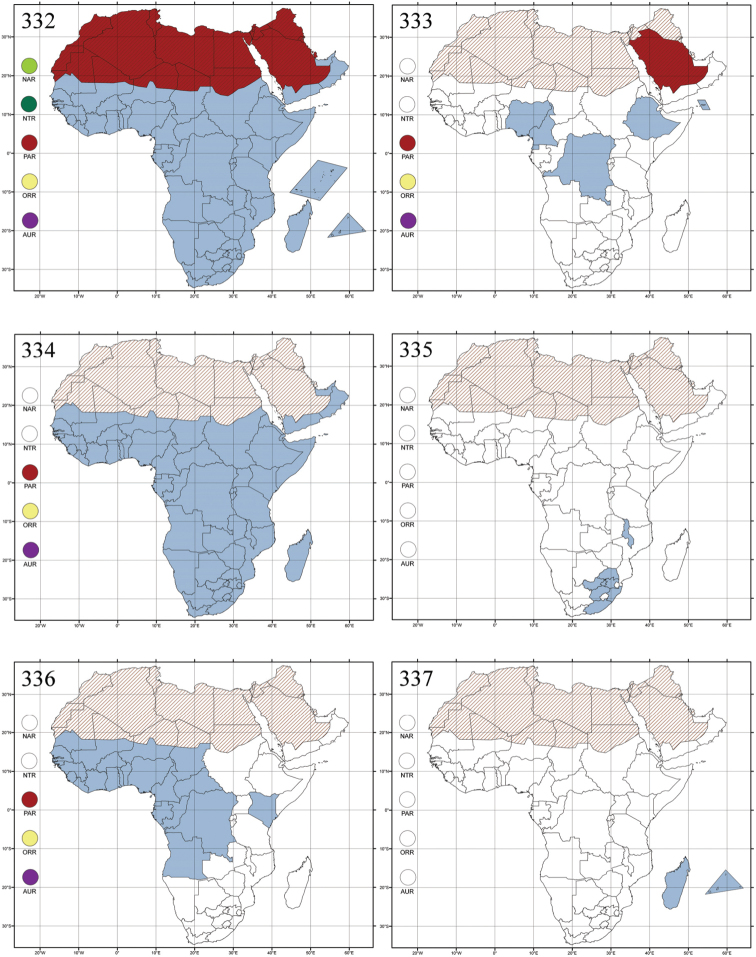
Maps of distribution. **332** *Longitarsus* Berthold **333** *Luperomorpha* Weise **334** *Lypnea* Baly **335** *Malvernia* Jacoby. **336** *Manobia* Jacoby **337** *Metroserrapha* Bechyné.

**Figures 338–343.**
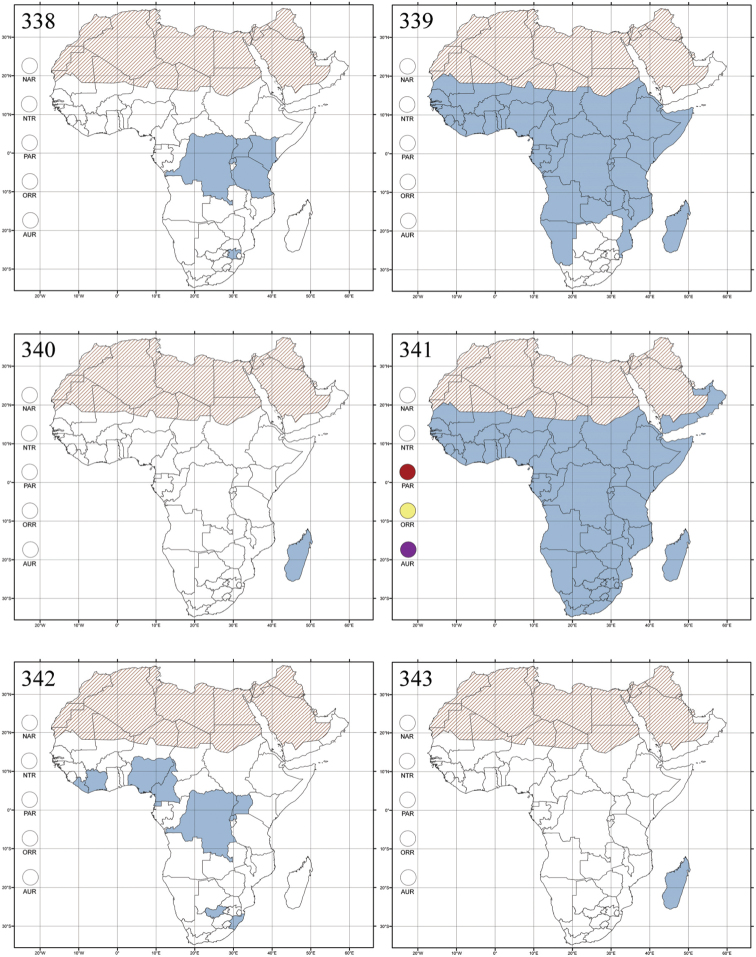
Maps of distribution. **338** *Montiaphthona* Scherer **339** *Myrcina* Chapuis **340** *Neodera* Duvivier **341** *Nisotra* Baly. **342** *Notomela* Jacoby **343** *Ntaolaltica* Biondi & D’Alessandro.

**Figures 344–349.**
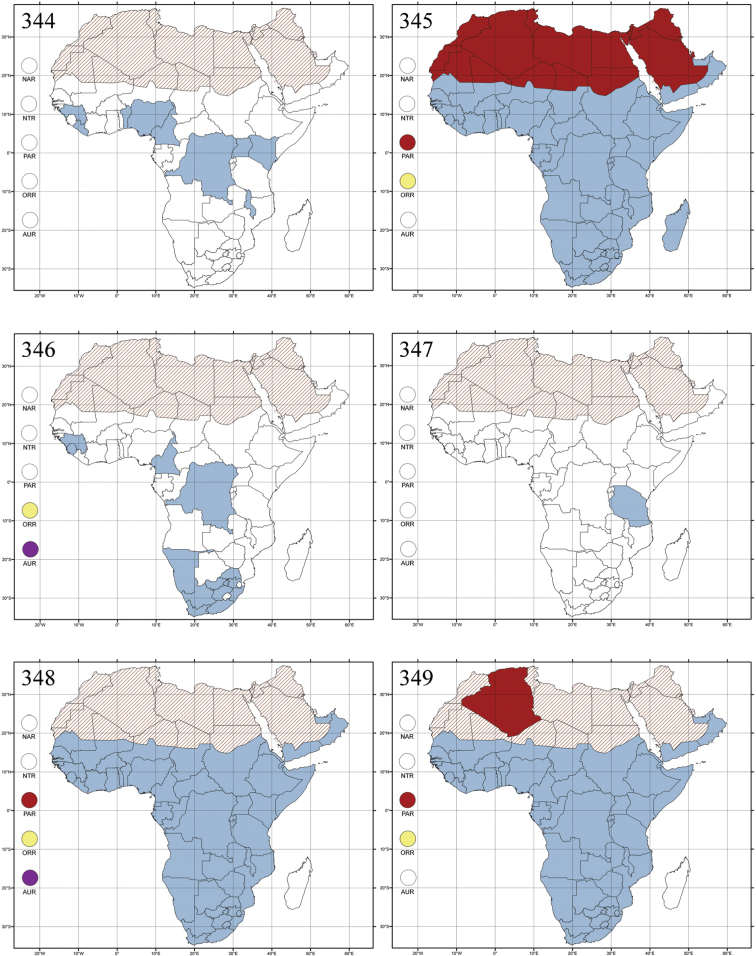
Maps of distribution. **344** *Nzerekorena* Bechyné **345** *Orthocrepis* Weise **346** *Paradibolia* Baly **347** *Perichilona* Weise **348** *Philopona* Weise **349** *Phygasia* Chevrolat.

**Figures 350–355.**
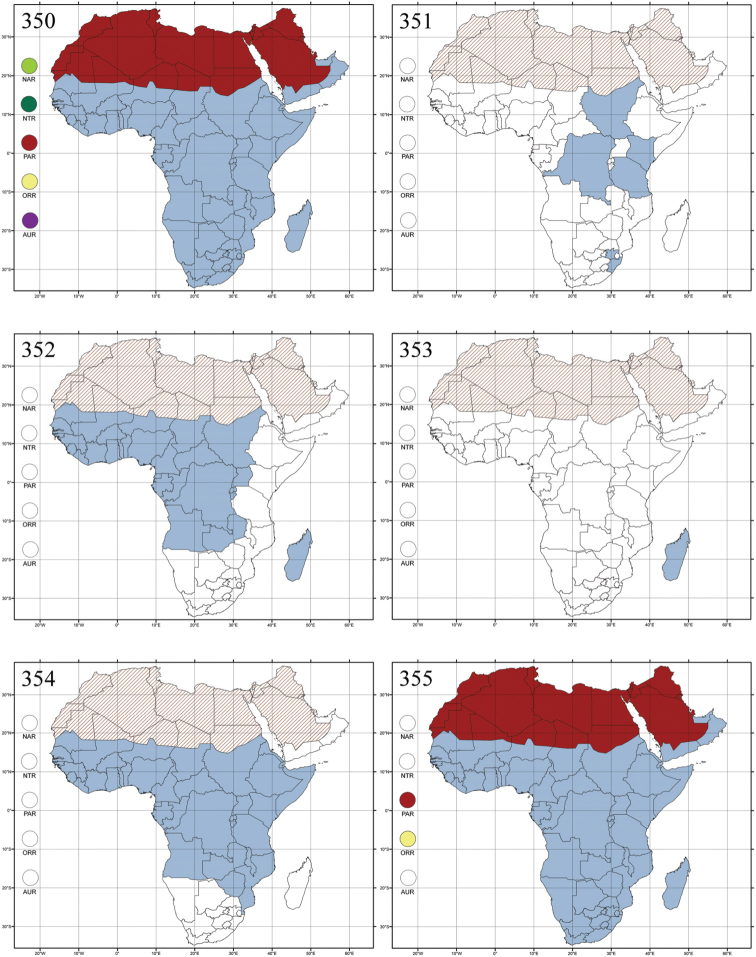
Maps of distribution. **350** *Phyllotreta* Chevrolat **351** *Physodactyla* Chapuis **352  ***Physoma* Clark **353** *Physomandroya* Bechyné **354** *Physonychis* Clark **355** *Podagrica* Chevrolat.

**Figures 356–361.**
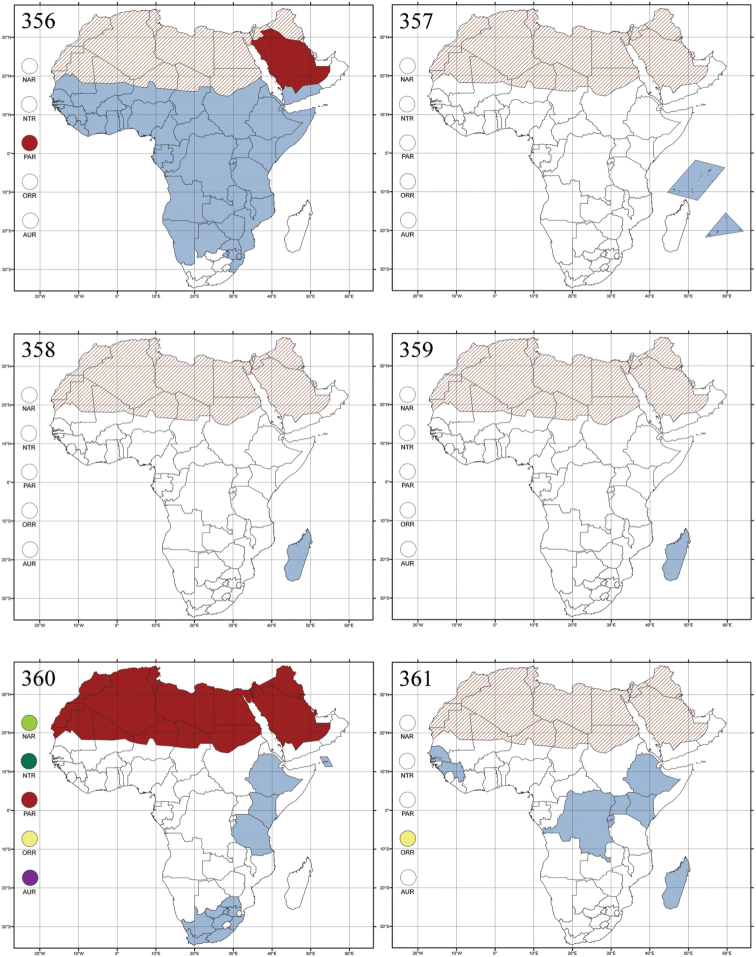
Maps of distribution. **356** *Polyclada* Chevrolat **357** *Pratima* Maulik **358** *Pseudadorium* Fairmaire **359** *Pseudophygasia* Biondi & D’Alessandro, in press **360** *Psylliodes* Berthold **361** *Sanckia* Duvivier.

**Figures 362–367.**
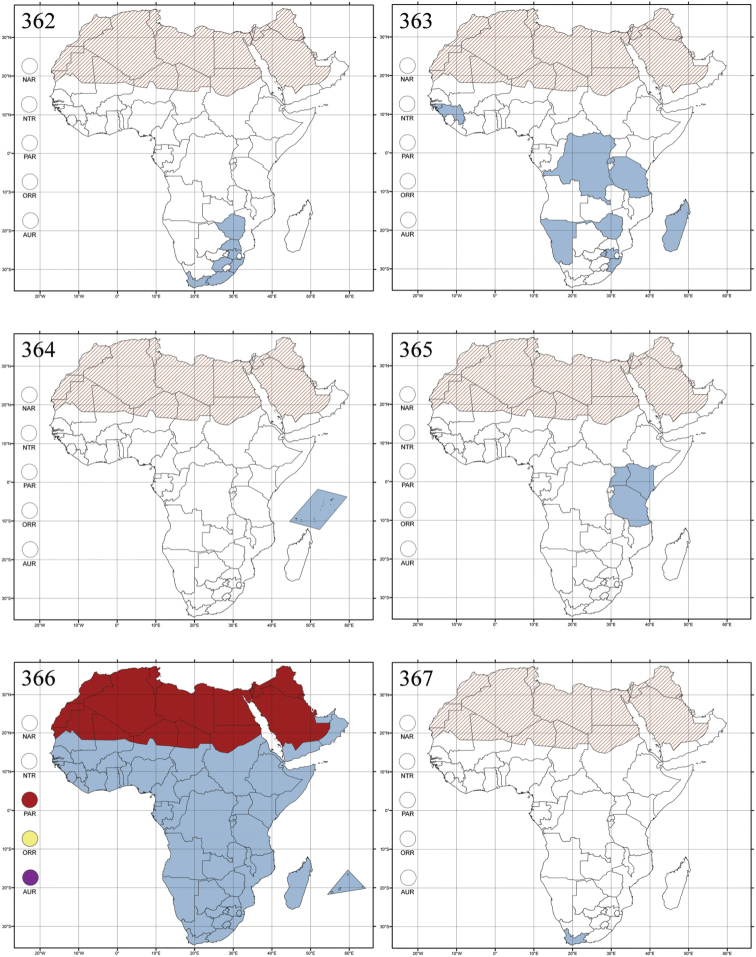
Maps of distribution. **362** *Serraphula* Jacoby **363** *Sesquiphaera* Bechyné **364** *Seychellaltica* Biondi **365** *Sjostedtinia* Weise **366** *Sphaeroderma* Stephens **367** *Stegnaspea* Baly.

**Figures 368–373.**
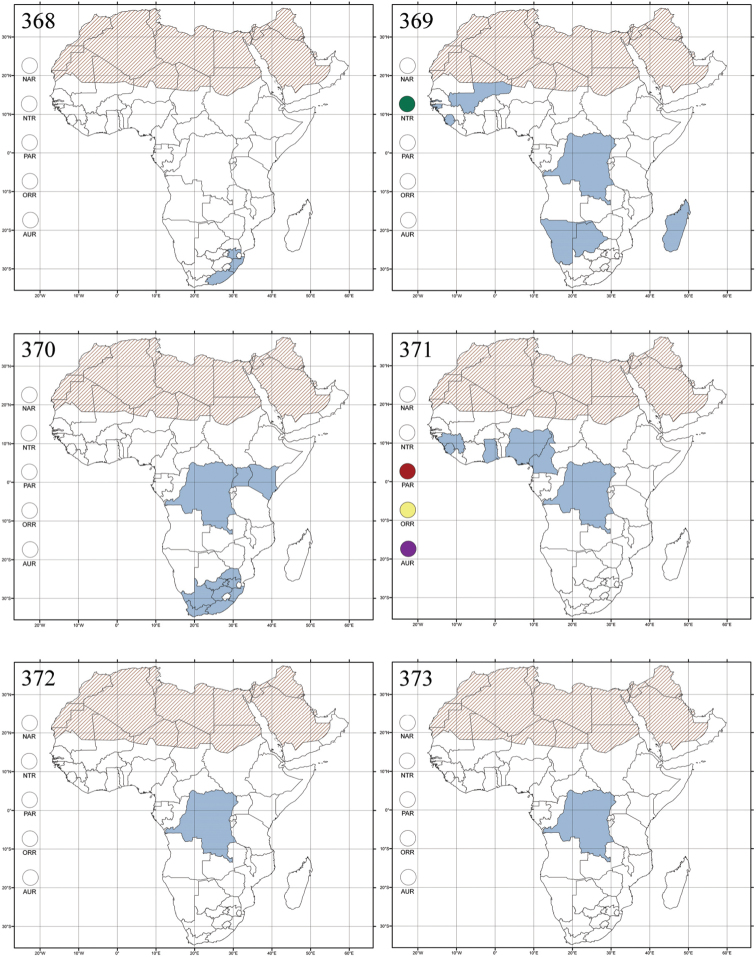
Maps of distribution. **368** *Stuckenbergiana* Scherer **369** *Terpnochlorus* Fairmaire **370** *Toxaria* Weise **371** *Trachytetra* Sharp **372** *Tritonaphthona* Bechyné **373** *Upembaltica* Bechyné.

**Figures 374–376.**
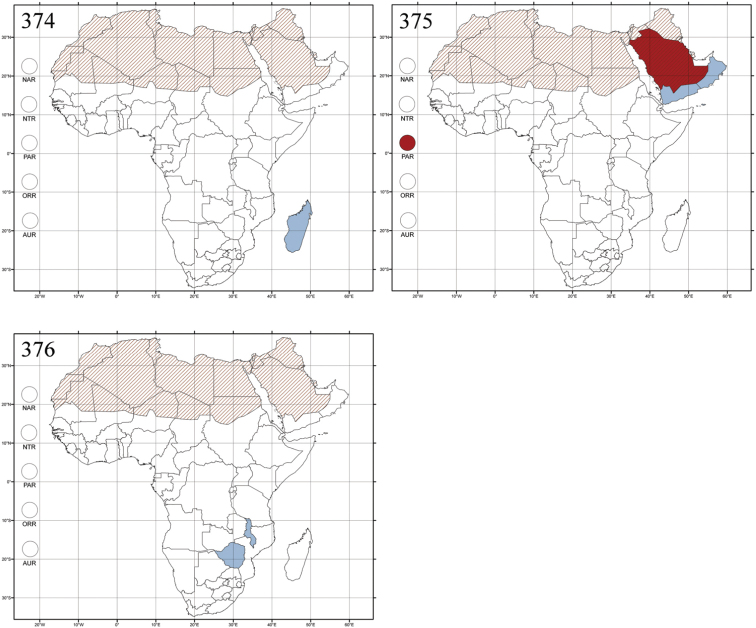
Maps of distribution. **374** *Xanthophysca* Fairmaire **375** *Yemenaltica* Scherer **376 ***Zomba* Bryant.

**Figure 377.**
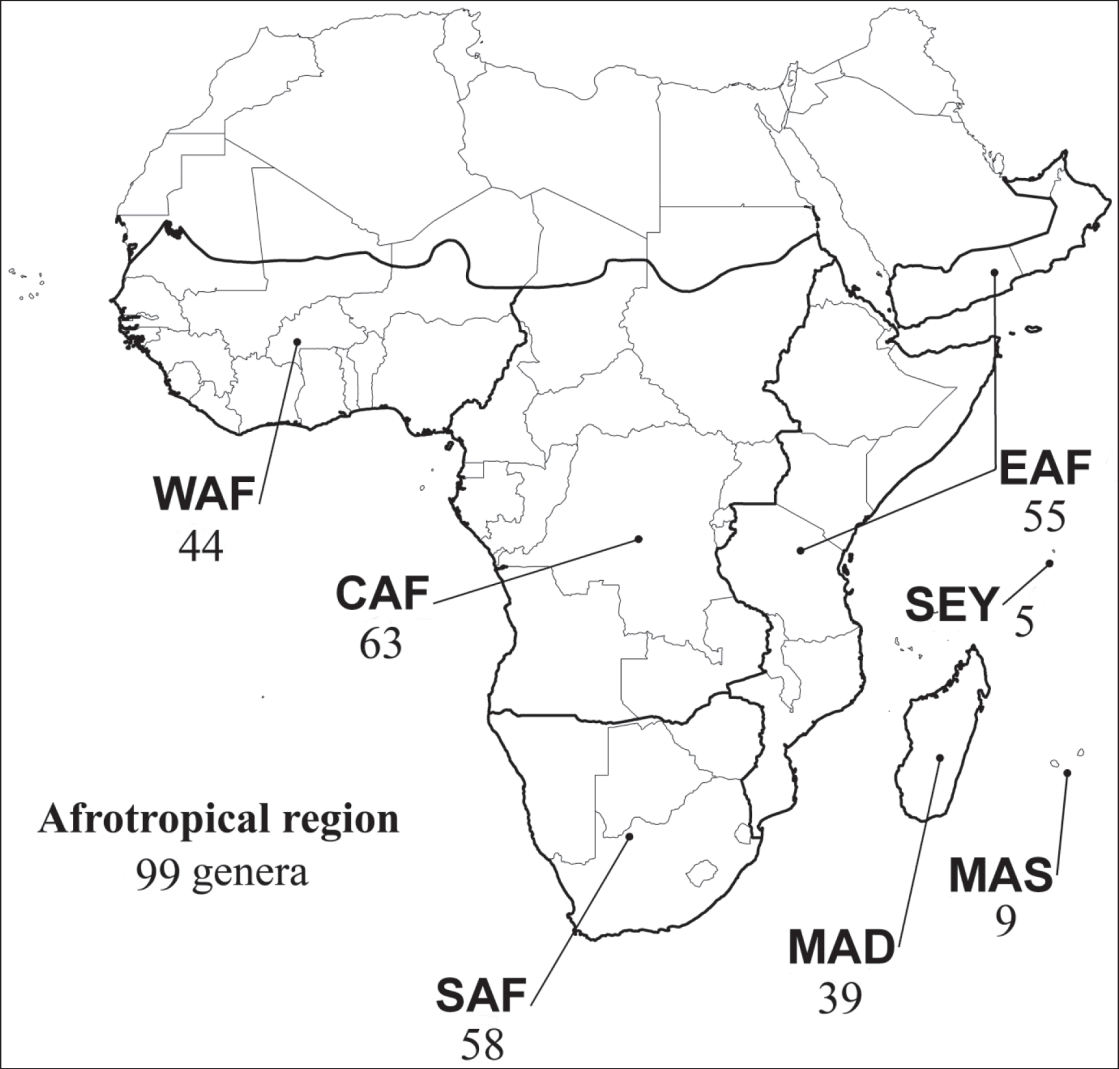
Number of flea beetle genera occuring in the various areas of the Afrotropical region (**CAF**: Central Afrotropical; **EAF**: Eastern Afrotropical; **MAD**: Madagascar; **MAS**: Mascarene Islands; **SAF**: Southern Afrotropical; **SEY**: Seychelles Islands; **WAF**: Western Afrotropical.) (updated from Biondi and D’Alessandro 2010a).

**Figure 378.**
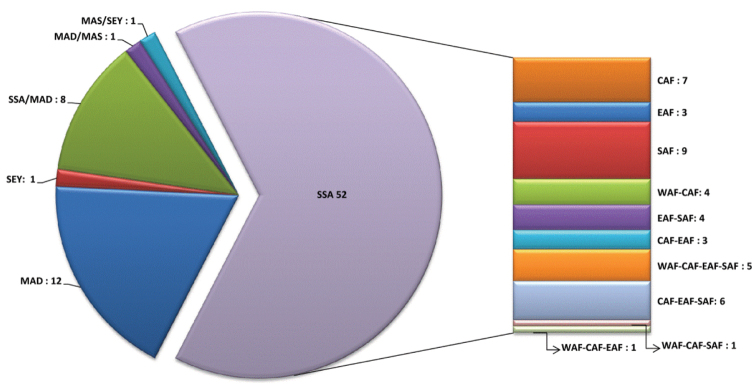
Number of flea beetle genera endemic to the various areas of the Afrotropical region (**CAF**: Central Afrotropical; **EAF**: Eastern Afrotropical; **MAD**: Madagascar; **MAS**: Mascarene Islands; **SAF**: Southern Afrotropical; **SEY**: Seychelles Islands; **SSA**: Sub-Saharan Africa; **WAF**: Western Afrotropical.) (updated from Biondi and D’Alessandro 2010a).

**Figure 379.**
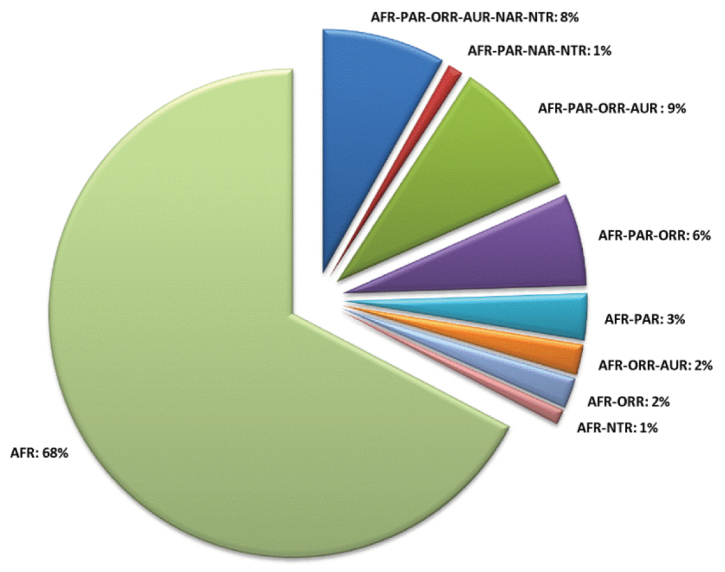
Distribution of Afrotropical flea beetle genera in the different zoogeographical regions (**AFR**: Afrotropical; **AUR**: Australian; **ORR**: Oriental; NAR: Nearctic; **NTR**: Neotropical; **PAR**: Palaearctic) (updated from Biondi and D’Alessandro 2010a).

**Figure 380.**
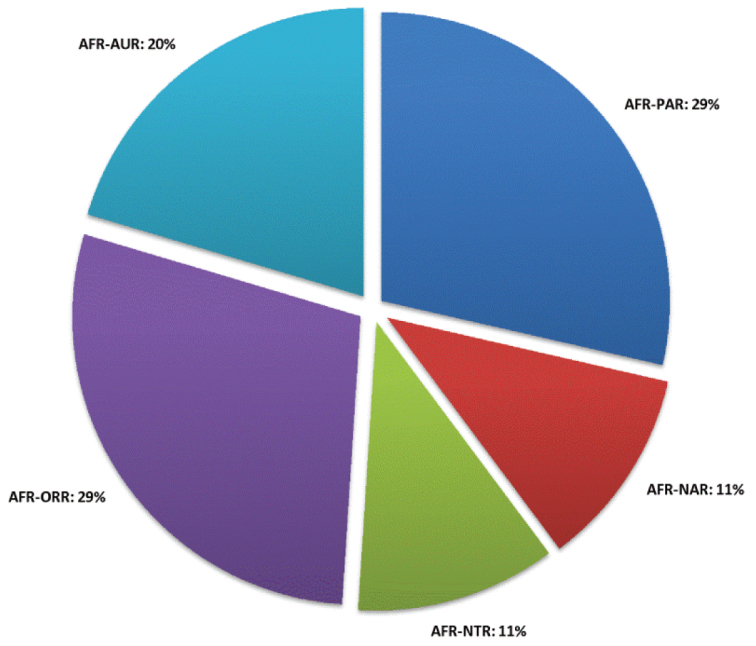
Percentage of flea beetle genera co-occuring in the Afrotropical and another zoogeographical region (**AFR**: Afrotropical; **AUR**: Australian; **ORR**: Oriental; **NAR**: Nearctic; **NTR**: Neotropical; **PAR**: Palaearctic) (updated from Biondi and D’Alessandro 2010a).

**Figure 381.**
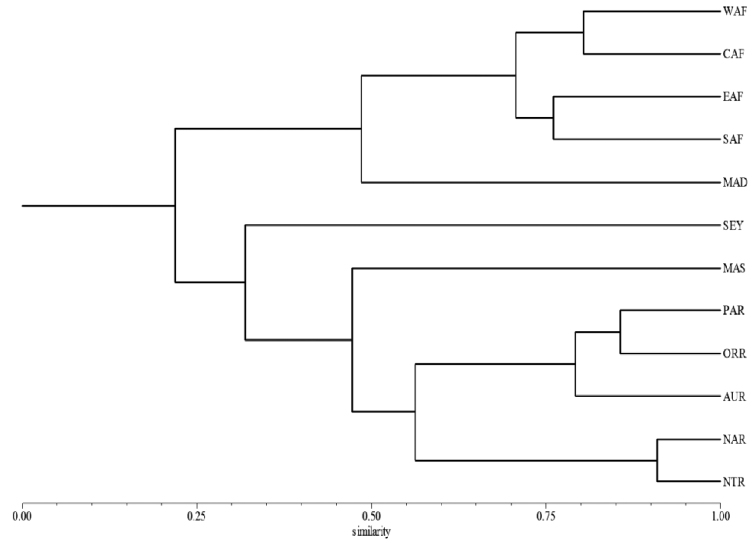
Faunistic similarities of the different areas of the Afrotropical region with those of other zoogeographical regions [Coincidence index and WPGMA clustering method (Weighted Pair Group Method using Arithmetic averaging) (cf. Biondi 2006)] (**AFR**: Afrotropical; **AUR**: Australian; **CAF**: Central Afrotropical; **EAF**: Eastern Afrotropical; **ORR**: Oriental; **MAD**: Madagascar; **MAS**: Mascarene Islands; **NAR**: Nearctic; **NTR**: Neotropical; **PAR**: Palaearctic; **SAF**: Southern Afrotropical; **SEY**: Seychelles Islands; **SSA**: Sub-Saharan Africa; **WAF**: Western Afrotropical) (modified from Biondi and D’Alessandro 2010a).

## Supplementary Material

XML Treatment for Abrarius

XML Treatment for Afroaltica

XML Treatment for Afrocrepis

XML Treatment for Afrorestia

XML Treatment for Alocypha

XML Treatment for Altica

XML Treatment for Amphimela

XML Treatment for Anaxerta

XML Treatment for Angulaphthona

XML Treatment for Antanemora

XML Treatment for Aphthona

XML Treatment for Argopistes

XML Treatment for Argopistoides

XML Treatment for Bangalaltica

XML Treatment for Bechuana

XML Treatment for Bechynella

XML Treatment for Bezdekaltica

XML Treatment for Bikasha

XML Treatment for Biodontocnema

XML Treatment for Blepharida

XML Treatment for Carcharodis

XML Treatment for Celisaltica

XML Treatment for Chabria

XML Treatment for Chaetocnema

XML Treatment for Chaillucola

XML Treatment for Chirodica

XML Treatment for Collartaltica

XML Treatment for Decaria

XML Treatment for Diamphidia

XML Treatment for Dibolia

XML Treatment for Dimonikaea

XML Treatment for Diphaulacosoma

XML Treatment for Djallonia

XML Treatment for Drakensbergianella

XML Treatment for Dunbrodya

XML Treatment for Epitrix

XML Treatment for Eriotica

XML Treatment for Eurylegna

XML Treatment for Eutornus

XML Treatment for Gabonia

XML Treatment for Guilielmia

XML Treatment for Guinerestia

XML Treatment for Halticotropis

XML Treatment for Hemipyxis

XML Treatment for Hespera

XML Treatment for Hildebrandtina

XML Treatment for Homichloda

XML Treatment for Hyphasis

XML Treatment for Jacobyana

XML Treatment for Kanonga

XML Treatment for Kenialtica

XML Treatment for Kimongona

XML Treatment for Lampedona

XML Treatment for Lepialtica

XML Treatment for Longitarsus

XML Treatment for Luperomorpha

XML Treatment for Lypnea

XML Treatment for Malvernia

XML Treatment for Manobia

XML Treatment for Metroserrapha

XML Treatment for Montiaphthona

XML Treatment for Myrcina

XML Treatment for Neodera

XML Treatment for Nisotra

XML Treatment for Notomela

XML Treatment for Ntaolaltica

XML Treatment for Nzerekorena

XML Treatment for Orthocrepis

XML Treatment for Paradibolia

XML Treatment for Perichilona

XML Treatment for Philopona

XML Treatment for Phygasia

XML Treatment for Phyllotreta

XML Treatment for Physodactyla

XML Treatment for Physoma

XML Treatment for Physomandroya

XML Treatment for Physonychis

XML Treatment for Podagrica

XML Treatment for Polyclada

XML Treatment for Pratima

XML Treatment for Pseudadorium

XML Treatment for Pseudophygasia

XML Treatment for Psylliodes

XML Treatment for Pydaristes

XML Treatment for Sanckia

XML Treatment for Serraphula

XML Treatment for Sesquiphaera

XML Treatment for Seychellaltica

XML Treatment for Sjostedtinia

XML Treatment for Sphaeroderma

XML Treatment for Stegnaspea

XML Treatment for Stuckenbergiana

XML Treatment for Terpnochlorus

XML Treatment for Toxaria

XML Treatment for Trachytetra

XML Treatment for Tritonaphthona

XML Treatment for Upembaltica

XML Treatment for Xanthophysca

XML Treatment for Yemenaltica

XML Treatment for Zomba
